# Supplementary material for: PharmacoSTORM nanoscale pharmacology reveals cariprazine binding on Islands of Calleja granule cells
Source: Nat Commun. 2021 Nov 11;12:6505. doi: 10.1038/s41467-021-26757-z (PMC8586358; doi:10.1038/s41467-021-26757-z)
Supplement: Supplementary file 1 — Supplementary Information [file 41467_2021_26757_MOESM1_ESM.pdf]

Supplementary Information

PharmacoSTORM nanoscale pharmacology reveals cariprazine binding on Islands of Calleja granule cells

Susanne Prokop, Péter Ábrányi-Balogh, Benjámin Barti, Márton Vámosi, Miklós Zöldi, László Barna, Gabriella M. Urbán, András Dávid Tóth, Barna Dudok, Attila Egyed, Hui Deng, Gian Marco Leggio, László Hunyady, Mario van der Stelt, György M. Keserű and István Katona

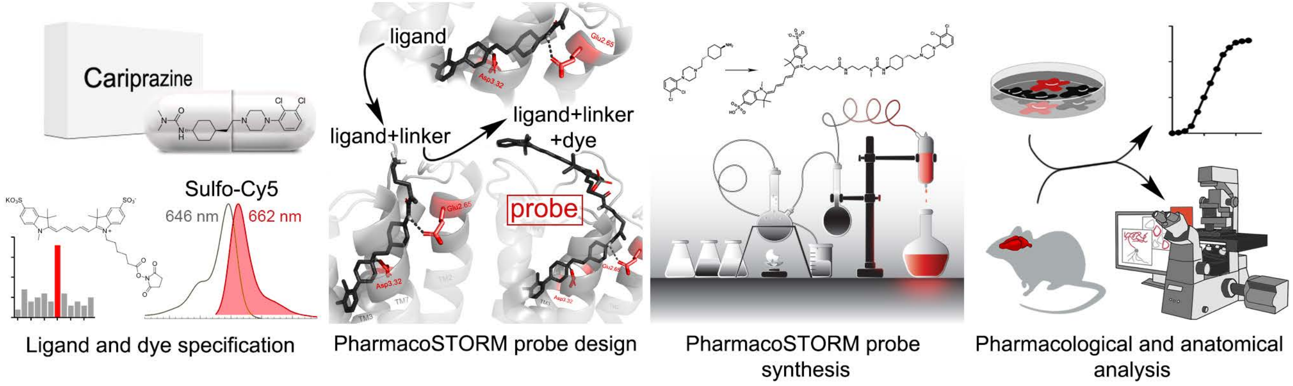

Table of Contents

(A) Supplementary Figures 3

(B) Supplementary Tables 31

(C) General considerations for designing PharmacoSTORM probes 35

Selection of the STORM dye 35

Selection of the ligand 35

Rational design of the PharmacoSTORM probe 36

Characterization of the PharmacoSTORM probe 37

(D) Synthesis of fluorescent pharmacoprobes for PharmacoSTORM experiments 37

Design and synthesis of ABP511 (fluo-cannabinoid), a fluorescent CB_1R agonist (1) for PharmacoSTORM experiments 37

Docking studies 38

Synthesis of ABP511 (1) (Supplementary Fig. S1e) 38

Design and synthesis of ABP535 (fluo-cariprazine), a D_3R partial agonist for PharmacoSTORM experiments (18) 39

Docking studies 40

Synthesis of the fluorescent cariprazine analogue (18) (Supplementary Fig. S4d) 40

Structural basis for the D_3 preference of cariprazine over D_2 receptor (Supplementary Fig. S6).. 40

Design and synthesis of DH-463, a fluorescent inhibitor of MAGL enzyme for PharmacoSTORM experiments 41

Docking studies 41

Synthesis of the fluorescent activity-based probe DH-463 (34) (Supplementary Fig. S3a).......... 41

(E) Supplementary methods 42

General synthetic methods 42

(F) Synthesis of probes 43

Experimental procedures for the synthesis of ABP511, the fluo-cannabinoid (1) 43

Experimental procedures for the synthesis of ABP535, the fluo-cariprazine (18) 50

Experimental procedures for the synthesis of the fluorescent activity-based probe DH-463 (34).... 52

(G) NMR spectra of synthesized compounds 56

(H) Supplementary References 75

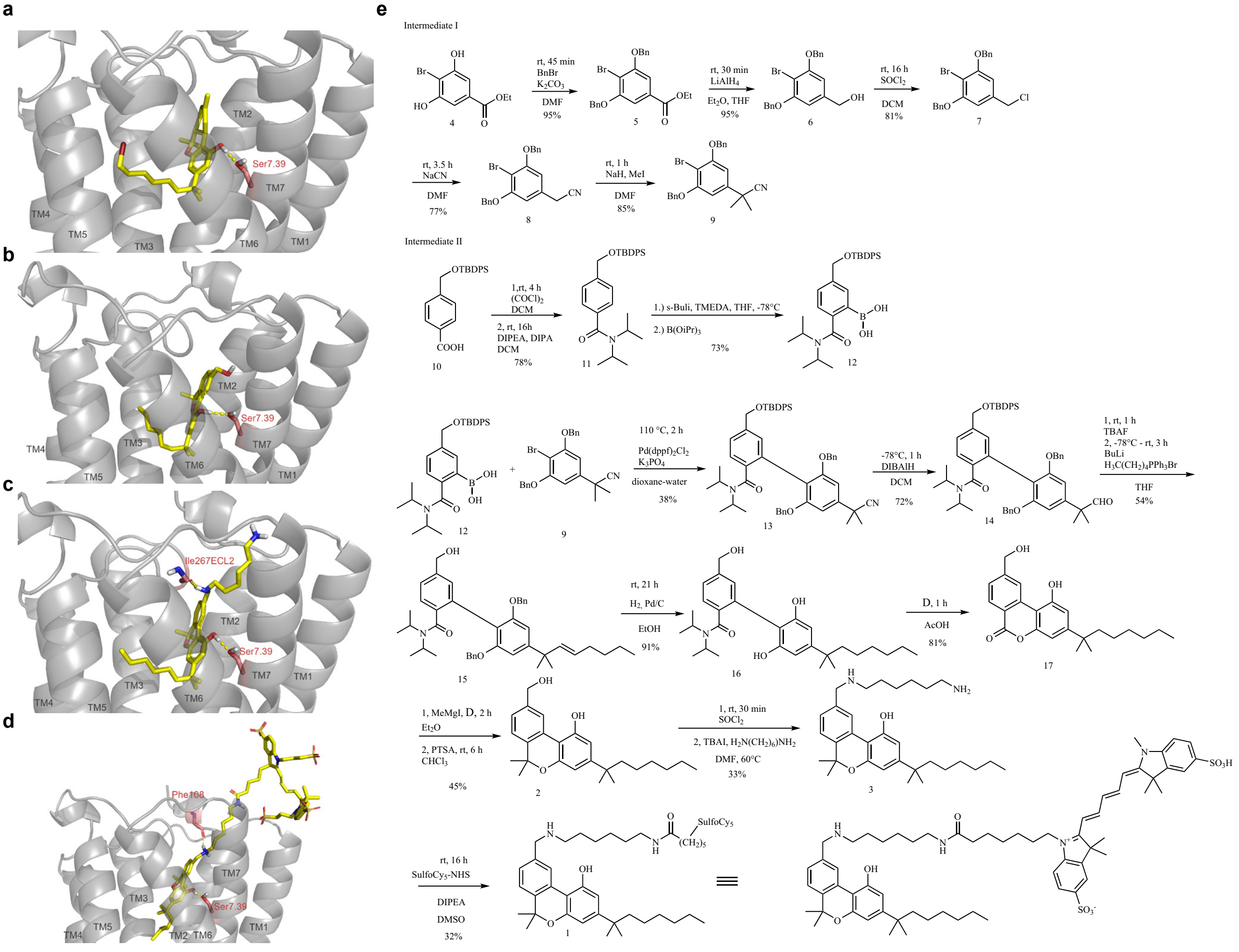

Supplementary Figure S1.

Supplementary Fig. S1. Design and synthesis of a fluorescent CB_1R agonist as a pharmacoprobe for PharmacoSTORM super-resolution imaging.

(a) The experimental binding mode of AM11542. See section (D) in the Supplementary Information for details.

(b,c,d) Docking poses obtained for **(b)** hydroxy-cannabinol (**2**); **(c)** hydroxy-cannabinol equipped with a linker (**3**); and **(d)** Sulfo-Cy5-labeled hydroxy-cannabinol (**1**, fluo-cannabinoid) in the human CB_1R structure. The receptor is represented as gray cartoon (PDB:5XRA); the ligands are yellow; key amino acid residues are red; and the hydrogen bonds with the protein residues are showed as yellow dashed lines.

(e) Synthesis of the fluorescent CB_1 agonist fluo-cannabinoid. See section (D) in the Supplementary Information for details.

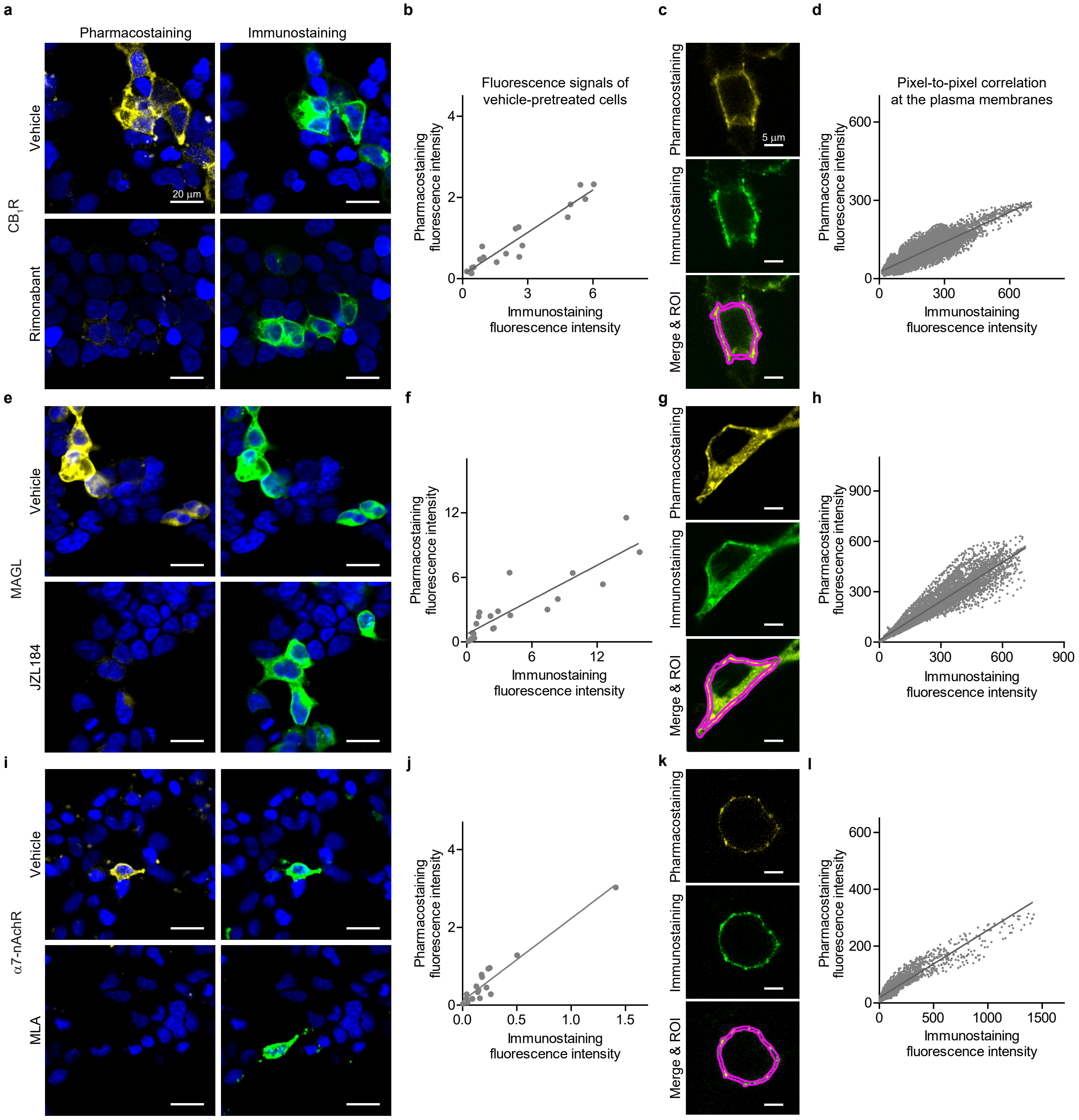

Supplementary Figure S2.

Supplementary Fig. S2. Correlated pharmacoprobe-based pharmacostaining and antibody-based immunostaining demonstrates quantitative power and high pharmacoprobe-labeling specificity for three major protein families.

(a,e,i) Confocal images of HEK 293 cell cultures, expressing molecular target proteins of PharmacoSTORM ligands selected from different protein classes. Proteins were simultaneously visualized by immunostaining against a fusion tag (GFP in the case of CB_1R and MAGL, or HA for α7-nAchR) and pharmacological labeling. Additional nuclear staining was performed with DAPI (blue). The lack of fluorescence signals in non-transfected cells demonstrates the high binding specificity of the pharmacoprobes and antibodies. Moreover, competitive ligand binding by using pretreatment with unlabeled receptor antagonists (rimonabant for CB_1 and MLA for α7-nAchR) or an enzyme inhibitor (JZL184 for MAGL) significantly reduced fluo-pharmacoprobe binding but did not affect antibody binding (lower panels).

(**a**) CB_1R-expressing cells were incubated with 100 nM fluo-cannabinoid after vehicle or 10 μM rimonabant pretreatment. (**e**) MAGL-expressing cells were incubated with 100 nM covalent fluorescent inhibitor (DH-463), with or without 10 μM JZL184 administration. (**i**) α7-nAchR-expressing cells were incubated with 1 ng/μl Alexa647-conjugated α-bungarotoxin with or without its selective antagonist (10 μM MLA). Scale bars are 20 μm for all images.

(b,f,j) Pharmacoprobe-based fluorescence intensity in randomly selected cells from the vehicle-treated groups positively scales with the expression levels of the respective target protein ($n = 21$, $n = 21$, $n = 18$ for CB_1R, MAGL, and α7-nAchR-expressing cells, respectively). The measurements were restricted to the plasma membrane. Linear regression curves were fitted to the data, and the goodness of fit is described by r^2 values: 0.92, 0.79, 0.92 for CB_1R, MAGL, and α7-nAchR-expressing cell cultures, respectively.

(c,g,k) Representative cells, randomly selected from correlated pharmacostaining and immunostaining images. Region-of-interests (ROIs) were restricted to the plasma membrane and are outlined in magenta on the merged images. Scale bars are 5 μm for all images.

(d,h,l) Pixel-to-pixel correlation of the measured fluorescence intensities obtained by different labeling methods from the ROIs. Linear regression curves were fitted to the data, and the goodness of fit is described by r^2 values. The r^2 values are: 0.77, 0.87, 0.87 for CB_1R, MAGL, and α7-nAchR-expressing cells on representative images (c,g,k), whereas median r^2 for all randomly selected membranes are 0.77, 0.81 and 0.77, respectively.

All data are presented in arbitrary units. All images and data are representative of 3 biologically independent experiments.

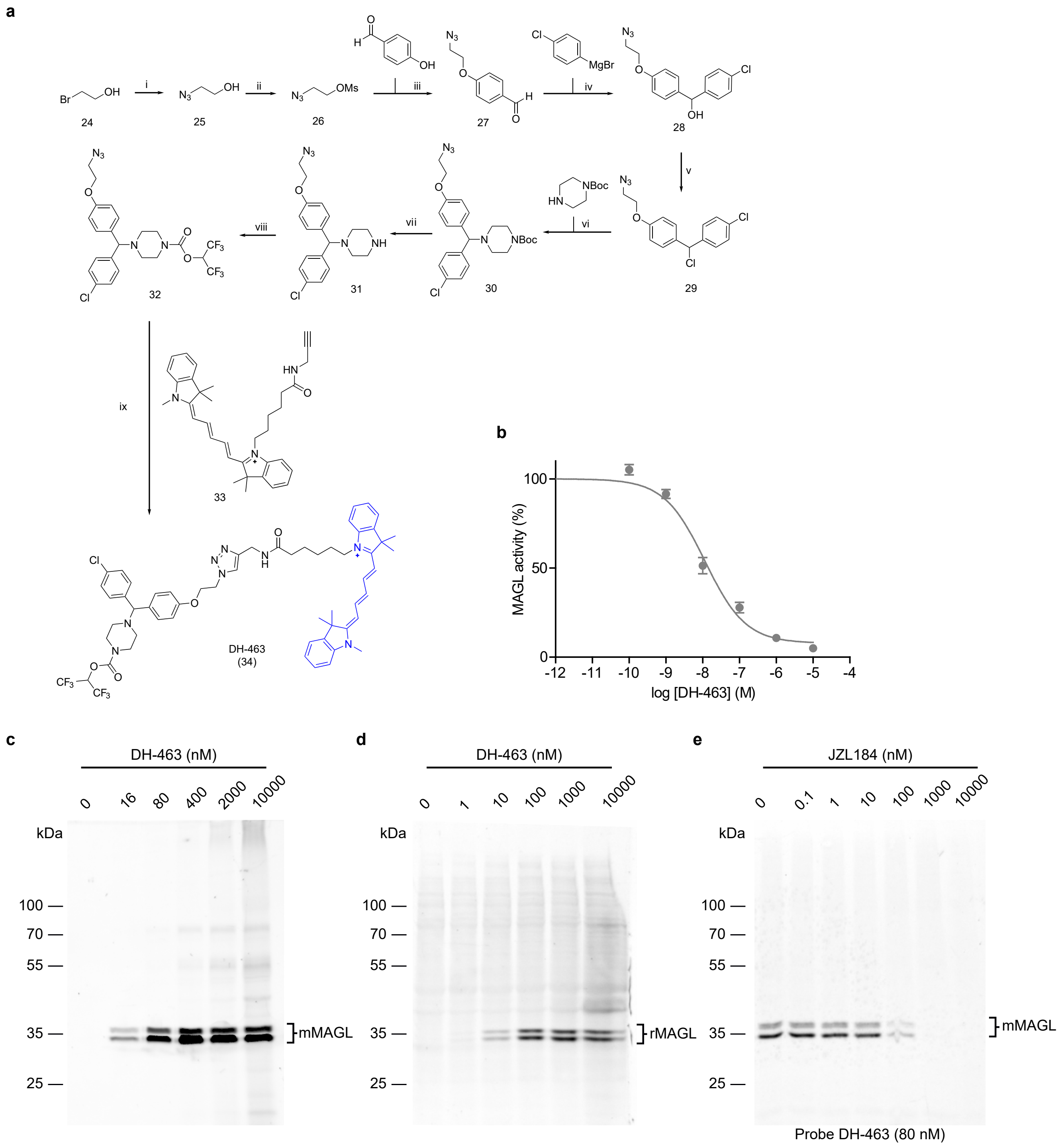

Supplementary Figure S3.

Supplementary Fig. S3. Synthesis and characterization of DH-463, the fluorescent MAGL enzyme inhibitor for PharmacoSTORM super-resolution imaging.

(a) Synthesis of DH-463 **(34)**. See section (D) in the Supplementary Information for details.
(b) Concentration-dependent inhibition curves of DH-463 against human MAGL as determined with 2-arachidonoylglycerol substrate assay. Data are presented as mean ± S.E.M, n = 4 biologically independent experiments.
(c,d) Gel-based activity-based protein profiling displays concentration-dependent labeling of MAGL activity with probe DH-463 (30 min) in mouse **(c)** or rat **(d)** brain membrane proteome.
(e) Competitive activity-based protein profiling with MAGL inhibitor JZL184 (0.1-10000 nM) in mouse brain membrane proteome using DH-463 (80 nM; 30 min). Preincubation with JZL184 prevented MAGL binding by DH-463 in a concentration-dependent manner.
All gel photos are representative for 3 biologically independent experiments.

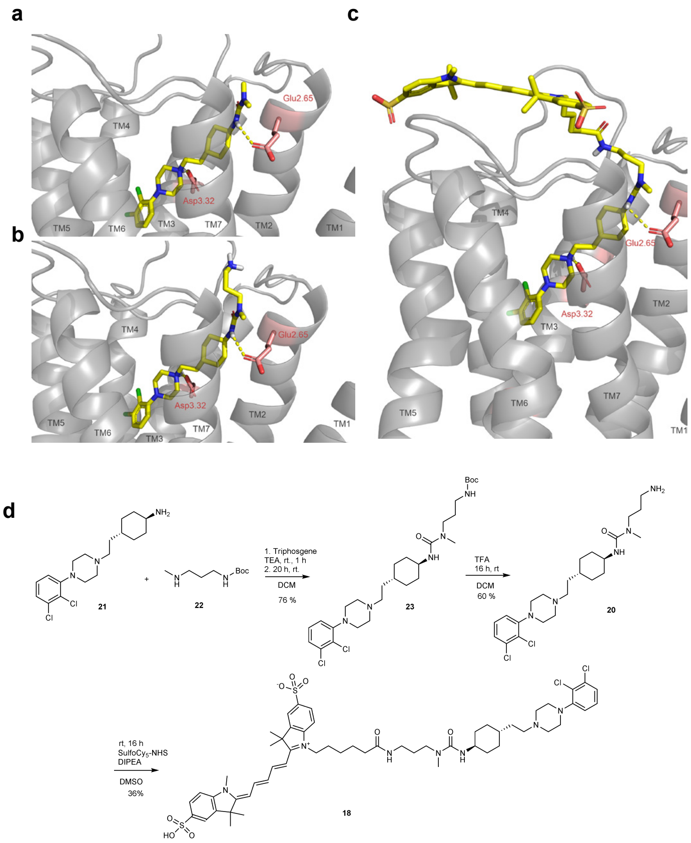

Supplementary Figure S4.

Supplementary Fig. S4. Design and synthesis of the fluorescent cariprazine analogue (18) for PharmacoSTORM experiments.

(a,b,c) Docking poses of cariprazine **(19**, **a),** cariprazine equipped with a linker **(20, b)** and Sulfo-Cy5-labeled fluo-cariprazine **(**Fluo-CAR=**18, c)** in the human D_3R. The receptor is represented as gray cartoon (based on PDB:6G79); the ligands are yellow and the hydrogen bonds with the key protein residues (red) are showed as yellow dashed lines. See section (D) in the Supplementary Information for details.

(d) Synthesis of fluo-cariprazine (**18**). See section (D) in the Supplementary Information for details.

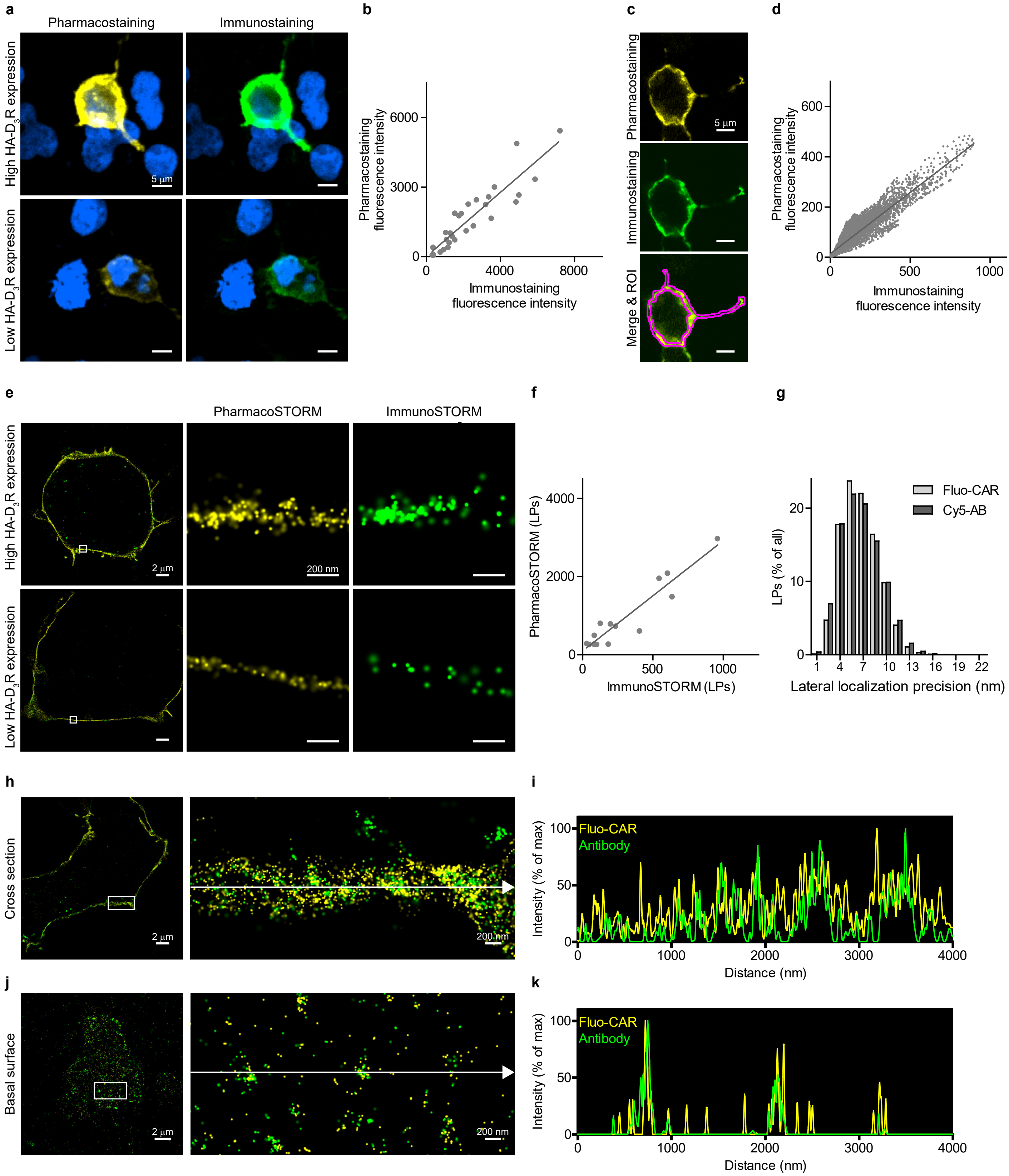

Supplementary Figure S5.

Supplementary Fig. S5. Fluo-cariprazine is an ideal pharmacoprobe for confocal microscopy and PharmacoSTORM super-resolution imaging.

(a) HEK 293 cells transiently transfected with HA-D_3R exhibit variable levels of D_3R expression. Confocal images of correlated anti-HA-based immunostaining (green) and pharmacostaining after incubation with 100 nM Fluo-CAR (yellow). Cell nuclei were visualized with DAPI (blue).

(b) Statistical analysis of the quantitative relationship between pharmacostaining and immunostaining. The respective fluorescence intensities were derived from Fluo-CAR binding and were obtained from the immunostaining of HA-tagged D_3Rs. The measurements were restricted to the plasma membrane and were performed on randomly selected cells (data are presented in arbitrary units, n = 29 cells). Linear regression analysis showed that the goodness of linear curve fit was $r^2 = 0.83$.

(c,d) Overlapping distribution of Fluo-CAR binding sites and HA-D_3Rs localization in the plasma membrane. **(c)** Confocal images of a representative cell and its plasma membrane region of interest (ROI) outlined on the merged image in magenta. **(d)** Pixel-to-pixel correlation of fluorescence intensities from the plasma membrane ROIs of the same representative cell. Goodness of linear curve fit after linear regression analysis is statistically described as $r^2=0.87$ for the representative cell, whereas the median $r^2 =0.82$ for all measured cells. Data are presented in arbitrary units.

(e) High-resolution PharmacoSTORM and ImmunoSTORM images of the plasma membrane segments of HEK 293 cells exhibiting different receptor expression levels. Anti-HA antibody was visualized by using a CF568-conjugated secondary antibody, whereas Fluo-CAR was applied at 100 nM concentration and was visualized by the intrinsic fluorescence of SulfoCy5.

(f) The number of PharmacoSTORM and ImmunoSTORM localization points (LPs) representing Fluo-CAR binding sites and D_3R protein localization by HA-antibody labeling, respectively. Note the high correlation for ROIs restricted to the plasma membrane (n = 14 cells). $R^2 = 0.88$ was obtained for the linear curve fitted on the data points.

(g) Localization precision of Fluo-CAR molecules bound to D_3Rs based on photon number counts. To directly compare the spatial localization precision of pharmacoprobe-based and antibody-based STORM images, HA-D_3R-expressing cells were either treated with 100 nM Fluo-CAR or were immunostained with anti-HA primary and Cy5-conjugated secondary antibodies. Note that Cy5 preserved its excellent photo-chemical properties for single-molecule localization microscopy in its drug-attached, receptor-bound form and the blinking events

yielded equally high photon counts as with Cy5 that was conjugated to an antibody. Median lateral localization precision of Fluo-CAR- and antibody-derived STORM localization points (LPs) were 7.23 nm and 7.19 nm respectively. The number of LPs in the analysis are 157,658 for Fluo-CAR and 109,527 for Cy5-conjugated antibody.

(h-k) Nanoscale colocalization analysis of Fluo-CAR-based PharmacoSTORM imaging and HA-antibody-based ImmunoSTORM imaging on cross sections of the plasma membrane more distant from the coverslip **(h,i)**, and on the basal plasma membrane directly attached to the coverslip **(j,k)**. **(h,j)** Representative dual dSTORM images of the distinct membrane compartments. Boxed areas were used for the STORM signal intensity measurements, the intensity profiles (i,k) were plotted along the white arrows in the insets. **(i,k)** Mean STORM signal intensity along a 4000 nm long, 100 nm wide profile, with 5 nm sampling density. Detected intensities are scaled between the minimum and maximum values along the profile for both channels. Although neither labeling method could saturate the overexpressed receptors, the overlap between the two signals is remarkable.

All images are representative of 3 biologically independent experiments.

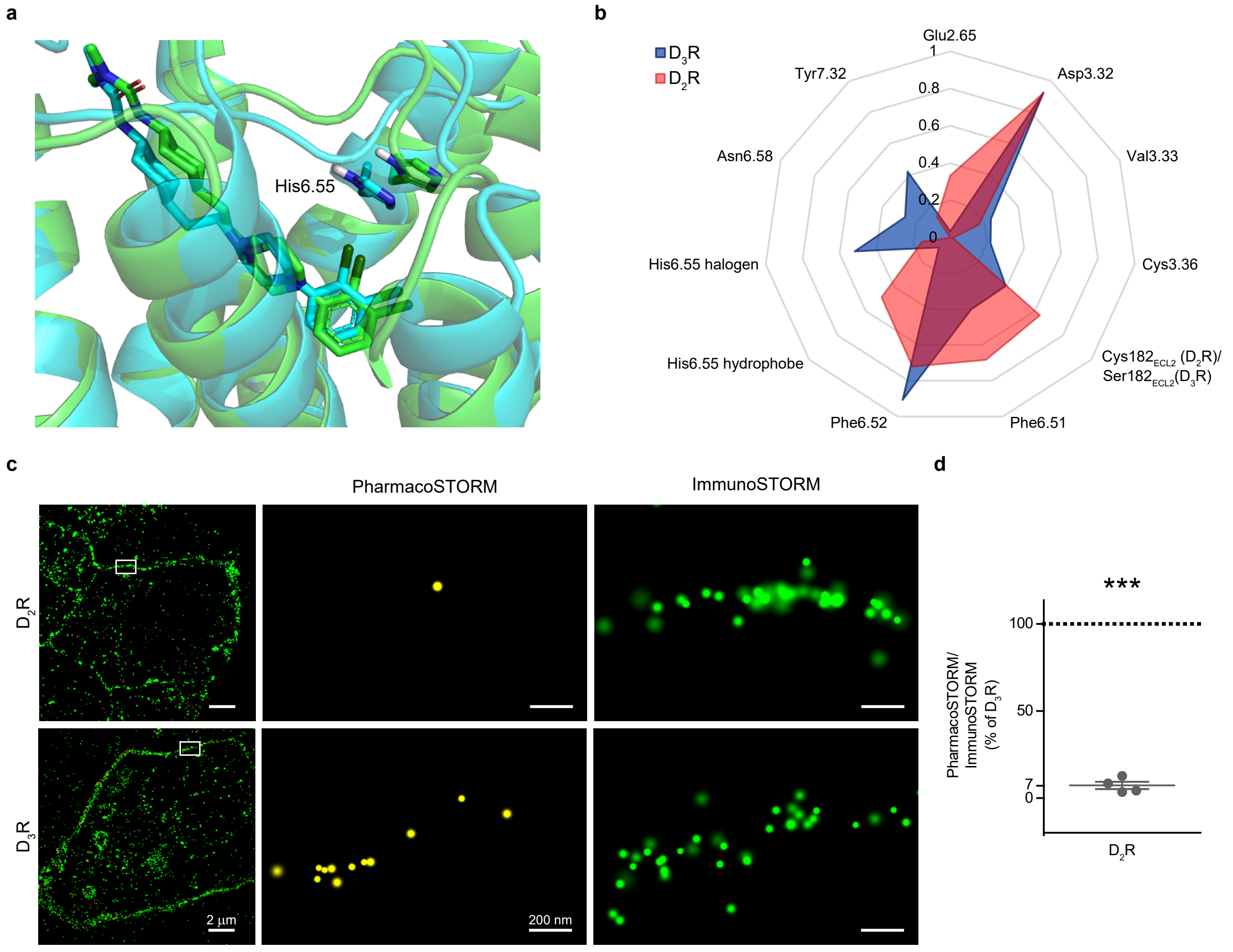

Supplementary Figure S6.

Supplementary Fig. S6. Strong preference of fluo-cariprazine binding to D_3R over D_2R.

(a,b) Important structural differences underlie dopamine receptor selectivity of low concentration cariprazine. The ligand was docked to the recently published active G_i bound human D_2R (PDB ID: 6VMS) and human D_3R (PDB ID: 7CMV) receptor structures. **(a)** Docking pose of cariprazine in the active human D_2R (green) and human D_3R (cyan) receptors with the His6.55 residue highlighted. **(b)** The most abundant interactions found between cariprazine and the D_2 (red) and the D_3 (blue) receptors in corresponding molecular dynamics (MD) simulations. Contacting amino acids are indicated in the radar diagram, the relative occurrence of the interactions is given in the concerted scale between 0 (no MD frame with this interaction) and 1 (the interaction was found in all MD frames). Interactions with His6.55 were halogen bond and hydrophobic contacts. Interactions with all the other residues were H-bonds. See section (D) in the Supplementary Information for details.

(c,d) Quantitative PharmacoSTORM imaging of fluo-cariprazine binding to different dopamine receptor subtypes. C-terminally Venus-tagged D_2 or C-terminally Venus-tagged D_3 receptor-expressing live HEK 293 cells were treated with 1.3 nM Fluo-CAR (the K_i value for D_3R). The tagged receptor proteins were also visualized by anti-Venus immunostaining after fixation. **(c)** Fluo-CAR molecules bound to both receptor subtypes. However, the extent of receptor labeling was substantially stronger for D_3R. **(d)** Relative receptor occupancy was determined as the ratio of PharmacoSTORM and ImmunoSTORM localization points (LPs) in the plasma membrane. Data from 4 biologically independent experiments are plotted as mean ± S.E.M, in % of values of D_3R-expressing cells in each experiment. Relative receptor occupancy in D_2R-expressing plasma membranes was on average 7.2% of D_3R. Two-tailed one sample t-test was performed against theoretical mean of 100 ($P = 4.71 \times 10^{-7}$).

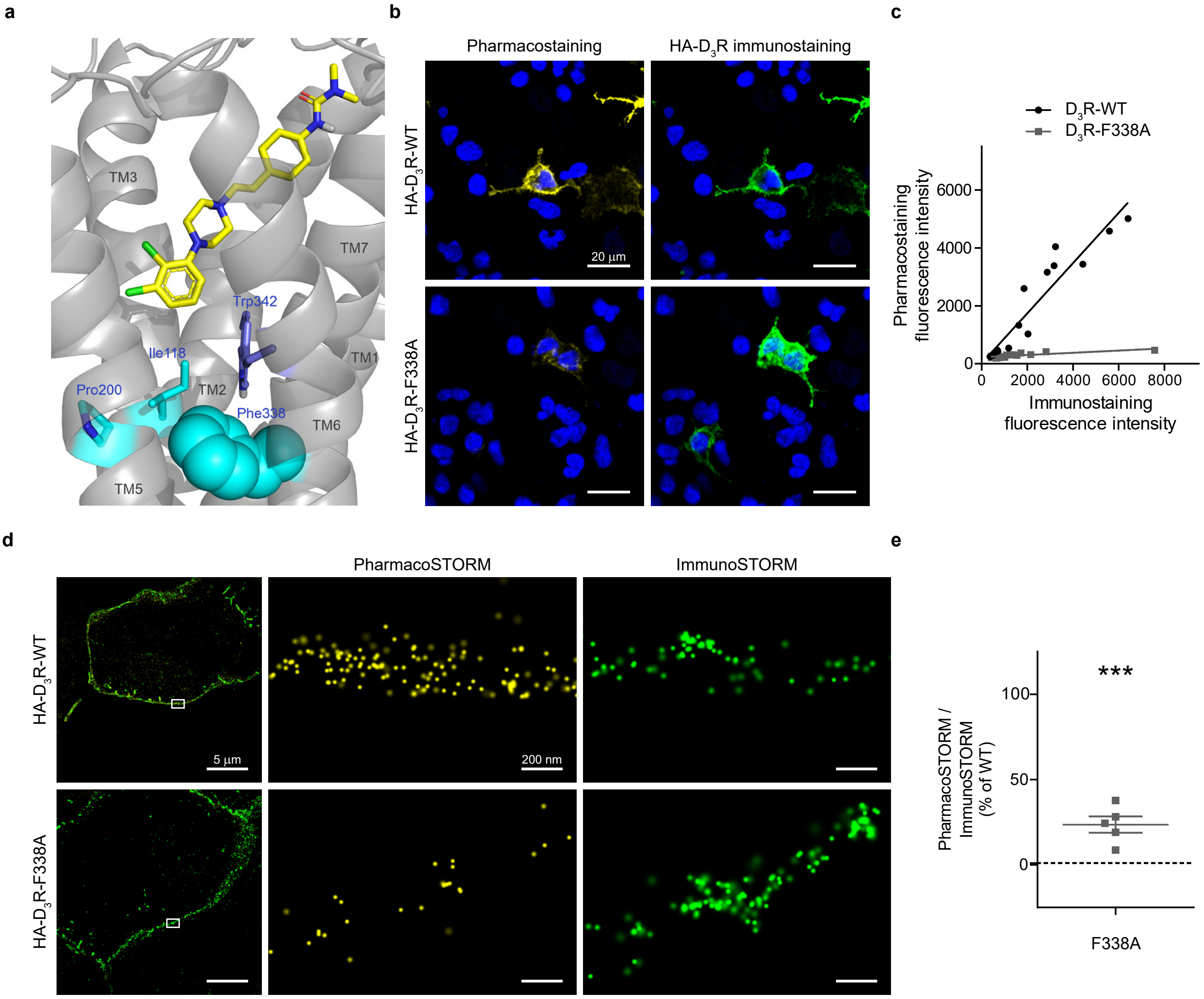

Supplementary Figure S7.

Supplementary Fig. S7. PharmacoSTORM-based evaluation of site-directed mutagenesis of the D_3 dopamine receptor.

(a) The docking pose of fluo-cariprazine (yellow) in human D_3R (gray). The side chains forming the so-called P-I-F binding motif are represented as cyan, whereas the toggle switch is shown in purple. Mutation of F338 (cyan spheres) is expected to trigger the reorientation of the P-I-F motif (P200, I118, F338) as well as the toggle switch (W342) that are both critical in GPCR activation[39,40]. The distorted hydrophobic interactions between the ligand and the receptor protein might lead to sub-optimal fitting of cariprazine.

(b) Confocal images of HEK 293 cells expressing HA-tagged wild-type (WT) or F338A mutant D_3Rs. Cultures were treated with 100 nM Fluo-CAR in parallel to anti-HA immunostaining. Nuclear staining was performed with DAPI (blue).

(c) Correlation of fluorescence intensity values arising from pharmacoprobe- and antibody-based labeling in the plasma membrane of randomly selected cells (n = 17 and n = 20 for WT and mutant receptors, respectively). Data are presented in arbitrary units. Linear regression curves were fitted to the data, and the goodness of fit is described by r^2 values: 0.90 and 0.67 for WT and F338 mutant receptors, respectively. Due to its reduced binding affinity, the curve belonging to the F338A-D_3R is flattened (the slope for F338A mutant curve = 0.04 and the slope for WT curve = 0.87).

(d) Dual direct STORM images of the plasma membranes of HEK 293 cells. Note that F338A mutation significantly reduces the PharmacoSTORM signal.

(e) The ratio of the number of PharmacoSTORM and ImmunoSTORM localization points is plotted to show that the relative receptor occupancy of the mutant D_3R is markedly reduced. The receptor occupancy was normalized to the value of the WT receptor in each experiment. Two-tailed one-sample t-test was performed on the data of the mutant receptor against the theoretical value of 100 (n = 5 biologically independent experiments, $P = 9.27 \times 10^{-5}$; data are presented as mean ± SEM).

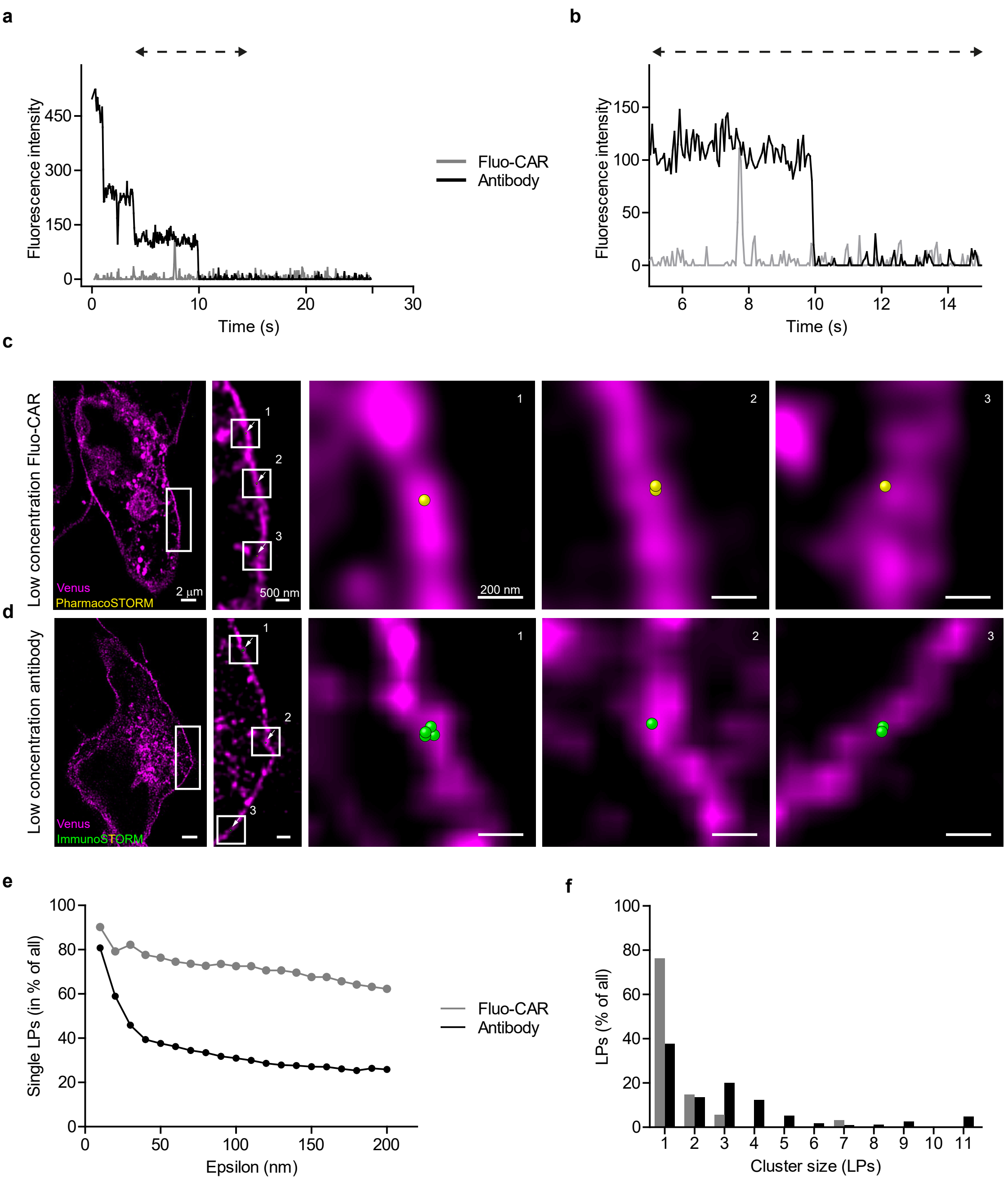

Supplementary Figure S8.

Supplementary Fig. S8. PharmacoSTORM has superior quantitative power over ImmunoSTORM.

(a,b) Representative fluorescence traces of individual probes on coverslip surface. Note the difference between the stepwise photobleaching of the antibody signal and distinct fluorescent peak from the Fluo-CAR molecule. The fluorescence intensity signal of Fluo-CAR is shorter in this experiment because this molecule only briefly adheres to the coverslip. Regular, but variable number of quantal photobleaching steps were observed in case of secondary antibodies suggesting that the number of conjugated fluorophores is not constant, because commercial production of these probes do not optimize for single fluorophore-tagging of the IgG molecule. In contrast, the chemical synthesis approach ensures that a single fluorophore is present on each pharmacoprobe. **(b)** Inset from **(a)** showing a shorter time window, including the time frames when a Fluo-CAR molecule was adhered to the coverslip. Importantly, the intensity value of the fluorescent ligand equaled to the unit intensity, that was measured in case of the secondary antibody. Data are presented in arbitrary units.

(c-f) An independent experimental assay in live cells was also established to further compare the photochemical properties of fluorescent probes. HEK 293 cells were transfected with N-terminally HA- and C-terminally Venus-tagged D_3R. Samples were exposed to low concentrations of either fluo-cariprazine or secondary antibody. This resulted in sparsely distributed "localization point (LP) clusters" in the plasma membrane. STORM blinking events of the different fluorescent probes were compared over a 5000-frame acquisition period. Insets depict three neighboring regions from the plasma membrane, where STORM signal was detected. This approach ensures that localization points that belong to the same "cluster" arise from a single probe with high probability.

(c) To achieve sparse PharmacoSTORM labeling, cells were labeled with 0.1 nM Fluo-CAR. The vast majority of receptor-bound Fluo-CAR molecules were detected as a single localization point in the plasma membrane.

(d) To characterize secondary antibodies in the same manner as Fluo-CAR **(c)**, cells were immunolabeled with low concentration (0.001 μg/ml) Alexa647-conjugated secondary antibody after incubation with anti-HA primary antibody. The number of blinking events were substantially more variable than in the case of the pharmacoprobe labeling.

(e,f) Quantitative analysis of blinking events. (**e)** The proportion of LPs without neighbors within ε distance. **(f)** The proportion of LPs within "clusters" of different sizes (ε = 50 nm).

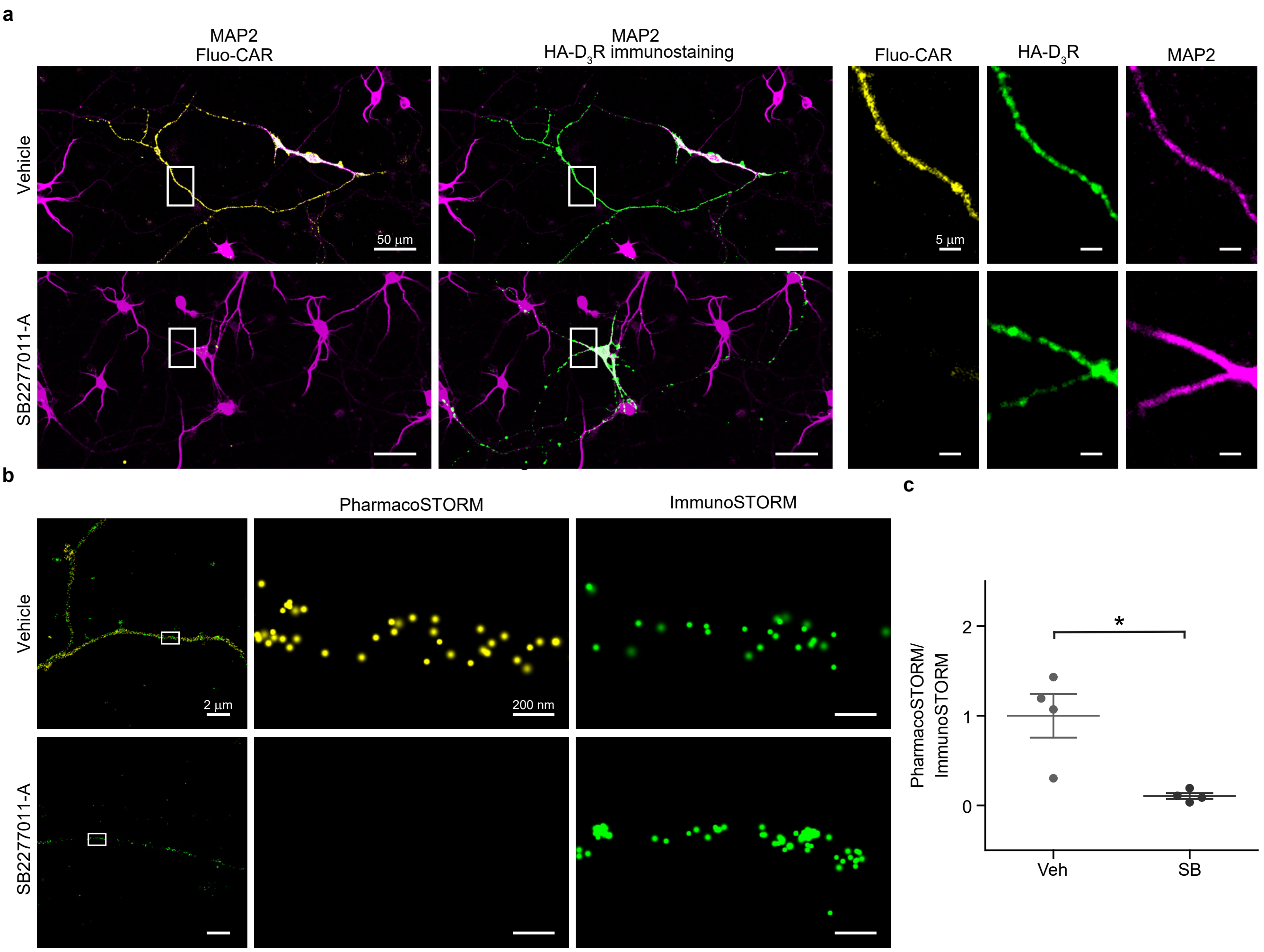

Supplementary Figure S9.

Supplementary Fig. S9. Subcellular domain-restricted visualization of D_3 receptors in primary hippocampal neuron cultures by fluo-cariprazine.

(a) Postnatal mouse primary hippocampal cultures were transiently transfected with HA-tagged D_3Rs. Live neurons were incubated with 100 nM Fluo-CAR and subsequently immunolabeled with anti-HA antibody. Further immunostaining against microtubule-associated protein 2 (MAP2) was performed to visualize the morphology of the dendritic tree of the transfected neurons. Images are representative of 3 biologically independent experiments with similar results.

(b) PharmacoSTORM super-resolution imaging of Fluo-CAR binding on neurites of transfected hippocampal neurons. Competitive ligand binding experiment was performed to validate the specificity of the PharmacoSTORM labeling. Notably, pretreatment with 10 μM SB277011-A, the D_3R antagonist completely prevented 100 nM Fluo-CAR binding to HA-D_3Rs.

(c) The ratio of PharmacoSTORM and ImmunoSTORM localization points was significantly reduced by SB277011-A (SB) pretreatment, compared to vehicle (Veh) pretreated cells. Two-tailed Mann-Whitney test was performed to evaluate statistical significance ($P = 0.0286$, $n = 4$, biologically independent experiments).

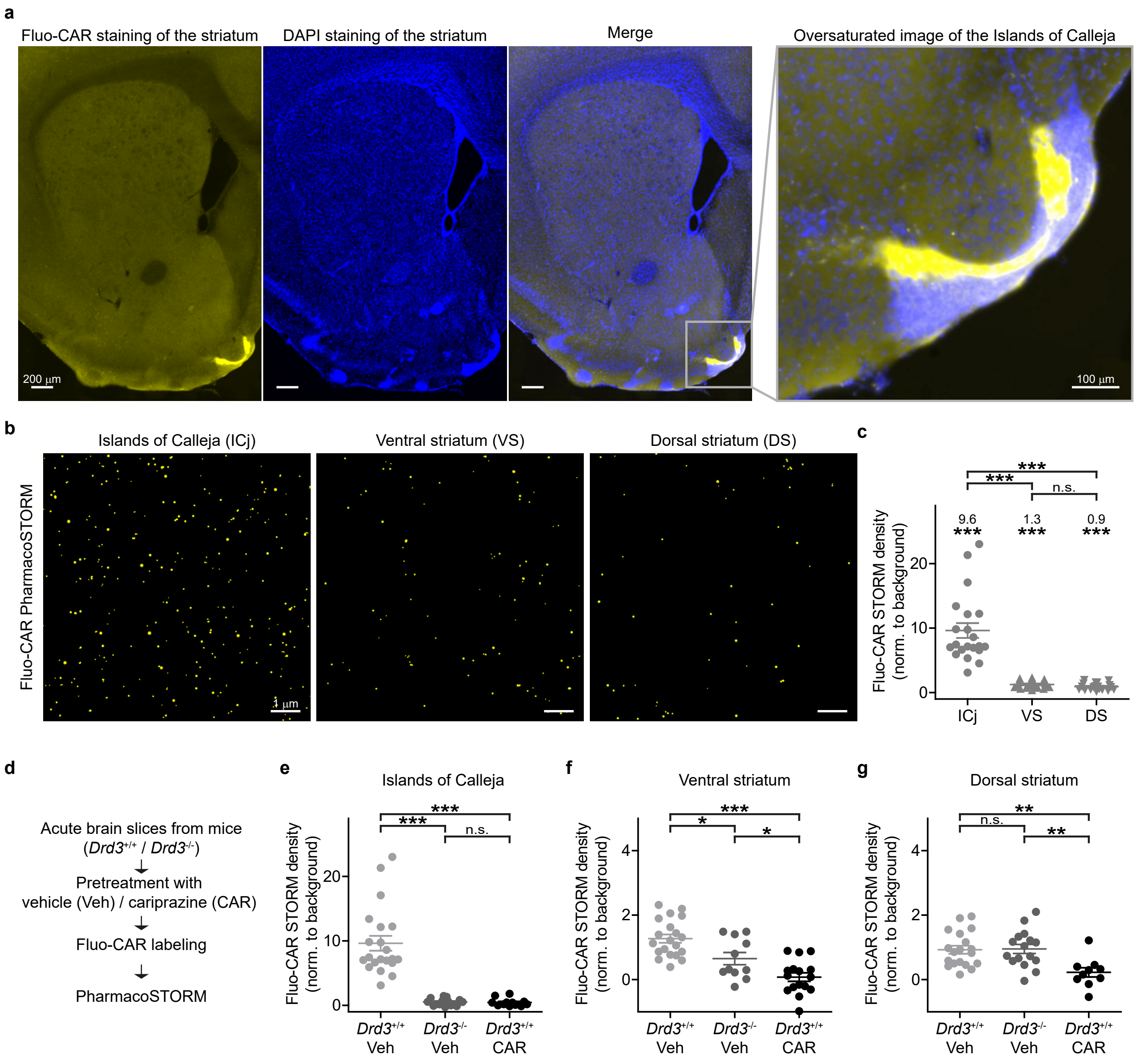

Supplementary Figure S10.

Supplementary Fig. S10. PharmacoSTORM imaging in the striatum reveals subregion-specific fluo-cariprazine binding density.

(**a**) Fluo-CAR (300 nM)-stained coronal brain slice captured by a high-sensitivity epifluorescence slide-scanning system. Image acquisition and processing parameters were set to optimize the visualization of the labeling profile in the low-binding density range. Consequently, Fluo-CAR binding-rich regions are oversaturated. Right panel shows higher magnification image of the ventromedial portion of the Islands of Calleja. Note that the Fluo-CAR signal is intentionally saturated in the Islands of Calleja to be able to illustrate the binding differences between the distinct anatomical areas.

(b) PharmacoSTORM images taken in the Islands of Calleja (ICj) and in the ventral and dorsal striatum (VS and DS). Note the higher number of localization points (LP, yellow) representing the fluo-cariprazine target engagement sites in the ICj compared to the VS and the DS.

(c) Statistical comparison of Fluo-CAR LP density in three different brain areas. Values were normalized to the background signal (assessed in brain slices non-treated with Fluo-CAR), then the background was subtracted. Wilcoxon signed rank tests were performed to test whether PharmacoSTORM LP densities were significantly different from 0 (ICj, n = 21 (from 9 animals), ***, $P = 9.54 \times 10^{-7}$; VS, n = 20 (from 4 animals), ***, $P = 1.91 \times 10^{-6}$; DS, n = 19 (from 4 animals), ***, $P = 3.81 \times 10^{-6}$), mean values of each group are indicated. Kruskal–Wallis test with Dunn's post-hoc test was performed to compare the Fluo-CAR signals (ICj vs. VS, ***, $P = 3.34 \times 10^{-6}$; ICj vs. DS, ***, $P = 3.66 \times 10^{-9}$; VS vs. DS, P = 0.6267).

(**d**) Summary of the experimental procedure. The detailed protocol is described in the Methods section. Competitive ligand binding experiments with cariprazine pretreatment (CAR, 30 μM) were applied to test the specificity of the PharmacoSTORM signal. The fraction of D_3Rs among the molecular targets of Fluo-CAR was assessed with *Drd3*$^{-/-}$ mouse line.

(**e**,**f**,**g**) Quantitative analyses of PharmacoSTORM images. Fluo-CAR signal was significantly reduced by cariprazine pretreatment in the ICj, VS and DS as well, indicating that PharmacoSTORM method can detect cariprazine target sites in brain areas with markedly different binding intensities. In the absence of D_3Rs, Fluo-CAR binding was significantly reduced in the ICj and in the VS. However, a significant amount of cariprazine-sensitive Fluo-CAR signal remained in the VS (nucleus accumbens). This indicates that Fluo-CAR labeled both D_3R and non-D_3R targets in the VS. In contrast, similar low-intensity, but cariprazine-sensitive, PharmacoSTORM signal was found in the DS of *Drd3*$^{+/+}$ and *Drd3*$^{-/-}$ mice (g), suggesting that Fluo-CAR molecules specifically bound to non-D_3R targets in this area.

(**e**) Kruskal–Wallis test with Dunn's post-hoc test was applied (*Drd3*$^{+/+}$ Veh, n = 21 (from 9 animals); *Drd3*$^{-/-}$ Veh, n = 13 (from 7 animals); *Drd3*$^{+/+}$ Car, n = 12 (from 4 animals); *Drd3*$^{+/+}$ Veh vs. *Drd3*$^{-/-}$ Veh, ***, P = 7.72 × 10^{-6}; *Drd3*$^{+/+}$ Veh vs. *Drd3*$^{+/+}$ Car, ***, P = 2.89 × 10^{-6}; *Drd3*$^{-/-}$ Veh vs. *Drd3*$^{+/+}$ Car, P > 0.9999). One-way ANOVA with Bonferroni post-hoc tests were performed in (**f**) (*Drd3*$^{+/+}$ Veh, n = 20 (from 4 animals); *Drd3*$^{-/-}$ Veh, n = 11 (from 4 animals); *Drd3*$^{+/+}$ Car, n = 16 (from 4 animals); *Drd3*$^{+/+}$ Veh vs. *Drd3*$^{-/-}$ Veh, *, P = 0.0178; *Drd3*$^{+/+}$ Veh vs. *Drd3*$^{+/+}$ Car, ***, P = 4.71 × 10^{-7}; *Drd3*$^{-/-}$ Veh vs. *Drd3*$^{+/+}$ Car, *, P = 0.042) and in (**g**) (*Drd3*$^{+/+}$ Veh, n = 19 (from 4 animals); *Drd3*$^{-/-}$ Veh, n = 16 (from 4 animals); *Drd3*$^{+/+}$ Car, n = 10 (from 4 animals); *Drd3*$^{+/+}$ Veh vs. *Drd3*$^{-/-}$ Veh, P > 0.9999; *Drd3*$^{+/+}$ Veh vs. *Drd3*$^{+/+}$ Car, **, P = 0.0048; *Drd3*$^{-/-}$ Veh vs. *Drd3*$^{+/+}$ Car, **, P = 0.0047).

Data are presented as mean ± S.E.M, dots represent the data of individual images in all panels.

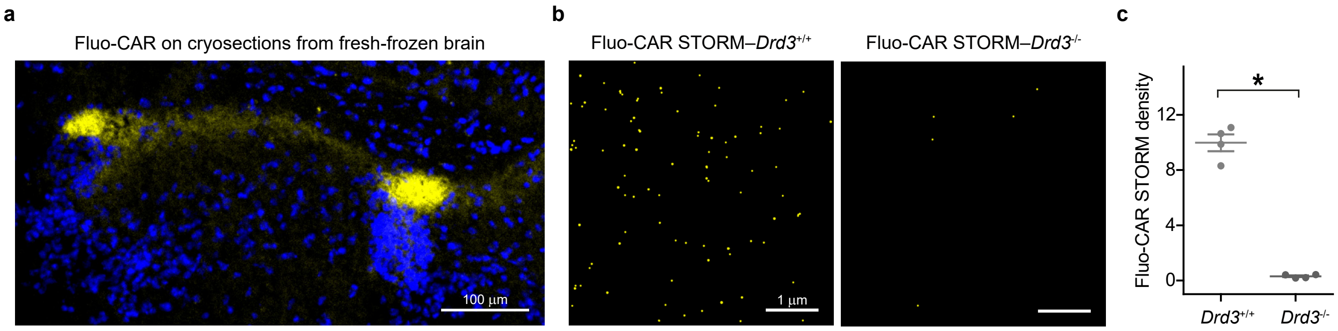

Supplementary Figure S11.

Supplementary Fig. S11. Visualization of fluo-cariprazine binding in fresh-frozen brain tissue by PharmacoSTORM imaging.

(a) Confocal image of a 20 μm-thick cryosection, incubated with 100 nM Fluo-CAR and DAPI. The most prominent pharmacoprobe labeling is located within the hilar subregion of the Islands of Calleja. The labeling results are representative of 3 biologically independent experiments.
(b) PharmacoSTORM super-resolution imaging of the nanoscale distribution of Fluo-CAR binding sites in the Islands of Calleja of *Drd3*$^{+/+}$ and *Drd3*$^{-/-}$ mice on brain tissue preparations obtained in a similar manner as in radioligand binding experiments.
(c) Scatter dot plot shows Fluo-CAR density (number of localization points/μm^2) measured in *Drd3*$^{+/+}$ mice (n = 4) and *Drd3*$^{-/-}$ mice (n = 4). Two-tailed Mann-Whitney test was performed to evaluate statistical significance (P = 0.0286).

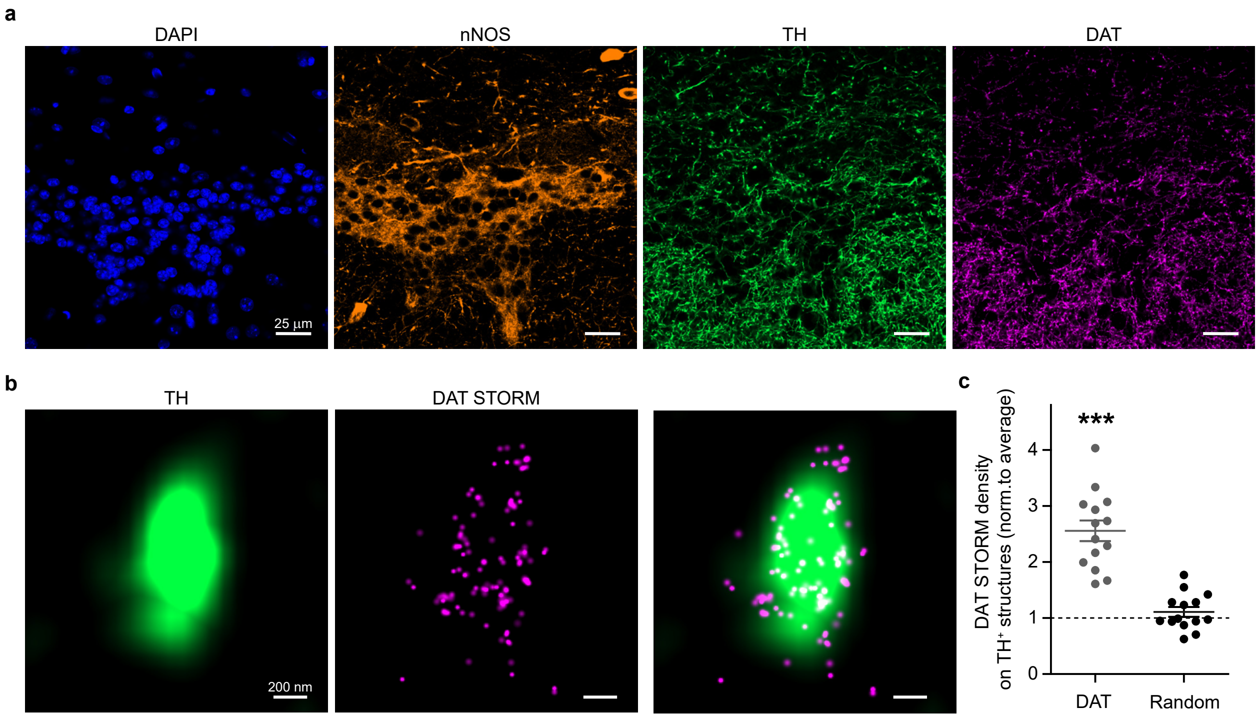

Supplementary Figure S12.

Supplementary Fig. S12. Dopaminergic innervation of the Islands of Calleja.

(a) Combined neuronal nitric oxide synthase (nNOS)-, tyrosine hydroxylase (TH)- and dopamine transporter (DAT)-immunostaining in the mouse ventral forebrain. The strong nNOS-immunopositive neuropil confirms that the cell cluster is part of the Islands of Calleja. The scattered distribution of both TH- and DAT-positive fibers verifies the dopaminergic nature of the monoaminergic innervation of this brain region.
(b) Correlated confocal and ImmunoSTORM imaging of dopaminergic terminals in the Islands of Calleja. Identical color channel combination, imaging parameters and image analysis were used as in the case of PharmacoSTORM imaging of fluo-cariprazine binding. Note that the DAT-ImmunoSTORM signal is restricted to the TH-positive nerve terminals.
(c) In contrast to the fluo-cariprazine binding sites (see Fig. 4), the STORM localization points (LPs), representing the position of dopamine transporters, are associated with TH-containing boutons. The same statistical evaluation was performed as in Fig. 4 and Fig. 5. As expected, DAT STORM LPs are highly abundant in profiles delineated by TH-immunostaining and are absent in the neighboring areas. Two-tailed Wilcoxon signed rank test was performed against the hypothetical mean of 1 ($P = 0.0001$ for DAT LPs and $P = 0.5416$ for randomly distributed LPs).
All images are representative of 3 biologically independent experiments.

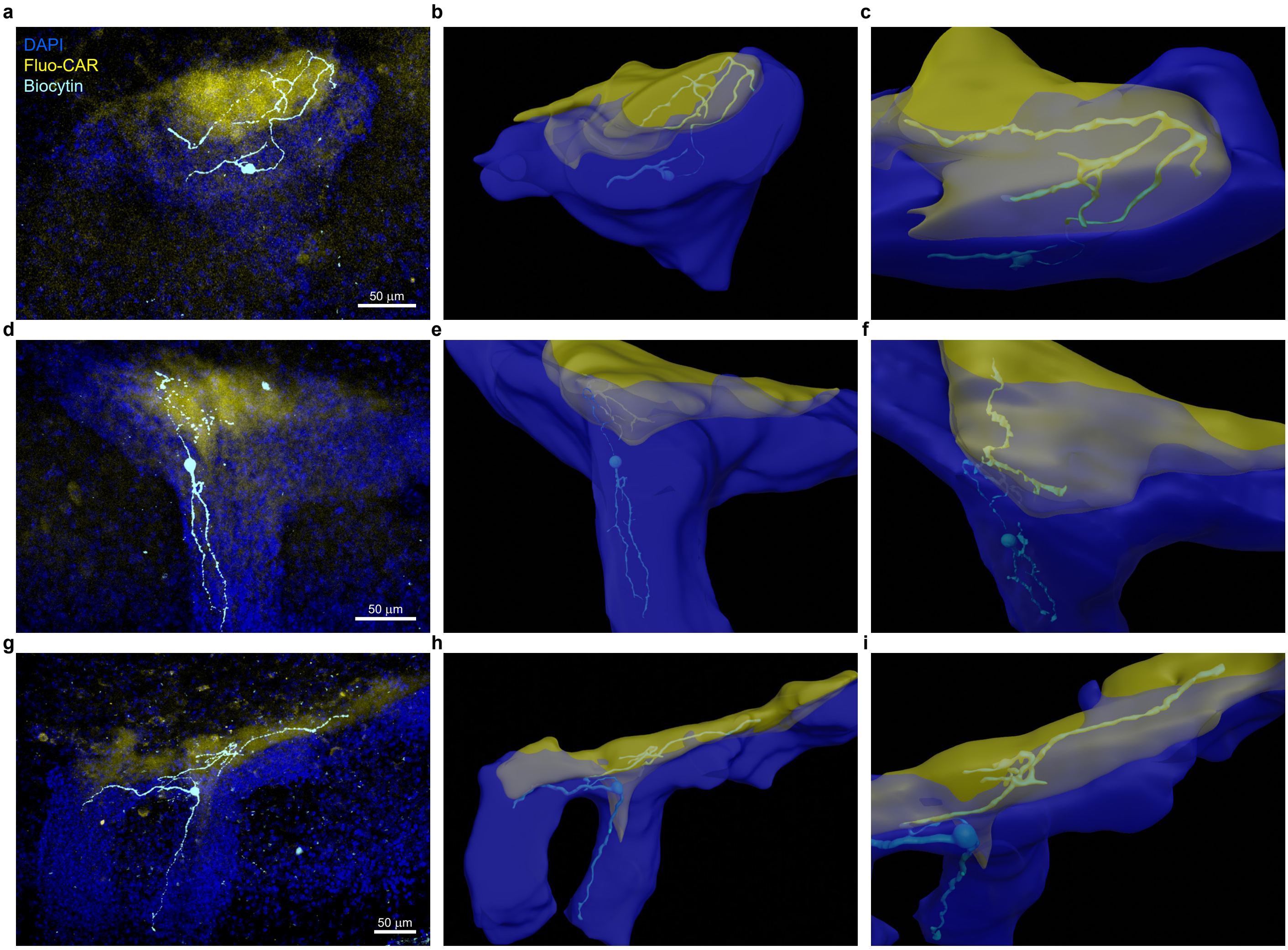

Supplementary Figure S13.

Supplementary Fig. S13. Three-dimensional comparative analysis of fluo-cariprazine binding sites and the neuronal morphology of Islands of Calleja granule cells.

(a,d,g) Granule cells in the Islands of Calleja in acute mouse coronal forebrain slices were filled with biocytin, and the slices were further labeled with Fluo-CAR and DAPI. Maximum intensity projections of confocal microscopic z-stacks are represented. Although the morphology of granule cells is highly variable, it always closely follows the geometrical shape of the Islands of Calleja.

(b,e,h) The 3-D models of granule cells and the Islands of Calleja were stitched and overlaid based on the microscopic stacks from **(a,d,g)** to reveal the exact three-dimensional anatomical relationships. To ease the understanding of the morphological details, the Fluo-CAR-rich hilar subregion (yellow) and the cell dense granular subregion (blue) are represented transparently.

(c,f,i) Higher-magnification top angle view shows the arborization of distinct granule cell processes within the hilus. Note that a single neurite from each granule cell selectively targets the Fluo-CAR-rich hilar subregion. Because this is a general characterizing morphological feature, these neurites were termed as hilar processes and were identified as axons by electron microscopy (see Fig. 6).

Images represent the diverse morphology of granule cells that follow the configuration of the Islands of Calleja. These observations were made in numerous animals (n = 13) during the study.

Supplementary Table S1.

Fluo-cannabinoid (ABP511) binding data (Eurofins)

Receptor	Species	Cell line	Radioligand (nM)	K_i (nM)
CB_1	Human	CHO	[^{3}H]CP 55940 (0.5)	3.5

Fluo-cariprazine (ABP535) binding data (Eurofins)

Receptor	Species	Cell line	Radioligand (nM)	K_i (nM)
D_3	Human	CHO	[^{3}H]Spiperone (0.7)	1.31
D_{2L}	Human	CHO	[^{3}H]Spiperone (0.16)	7.05
5-HT_{1A}	Human	CHO	[^{3}H]8-OH-DPAT (1.5)	27.3
5-HT_{2A}	Human	CHO-K1	[^{3}H]Ketanserine (0.5)	2660
5-HT_6	Human	HeLa	[^{3}H]LSD (1.5)	530
5-HT_7	Human	CHO	[^{3}H]LSD (1.5)	5.08

Supplementary Table S2.

Name	Sequence
hDRD3_fw	TATAAAGCTTGCCACCATGGCATCTCTGAGCCAGCTGAG
hDRD3_rev	TATAGATATCTCAGCAAGACAGGATCTTGAGGAAG
HA-hD3_fw	TATAAAGCTTGCCACCATGTATCCTTATGACGTCCCTGACTATGCCATGGCATCTCTGAGCCAGCTGAGTGG
mMGL_fw	TATAAAGCTTACATGCCTGAGGCAAGTTCACC
mMGL_rev	TATAGGATCCTCAGGGTGGACACCCAGCTC
CHRNA7_HA_fw	TATAGGATCCGCCACCATGCGCTGCTCGCCGGGAGGC
CHRNA7_HA_rev	TATACTCGAGTTAAGCGTAGTCCGGAACGTCGTACGGGTACGCAAAGTCTTTGGACACGGC
F338-fw	ATTGTGCTTGGGGCCGCCATTGTCTGCTGGCTG
F338-rev	CAGCCAGCAGACAATGGCGGCCCCAAGCACAAT
hDRD3_N1_fw	TATAAGATCTGCCACCATGGCATCTCTGAGCCAGCTGAG
hDRD3_N1_rev	TATAACCGGTGGGCAAGACAGGATCTTGAGGAAGG
hDRD2_fw	TATACTCGAGGCCACCATGGATCCACTGAATCTGTCCTGG
hDRD2_N1_rev	TATAACCGGTGGGCAGTGGAGGATCTTCAGGAAGG

Supplementary Table S3.

Target	Producer	Cat. No.	Dilution	Lot
**Primary antibodies**				
DARPP-32	Abcam	ab220808	1:500	GR3175773-1
DAT	Frontier Institute	DAT-GP-Af660	1:500	Not provided
GFP	Abcam	ab5450	1:1000	GR3251233-1, GR3215617-1
HA	Sigma-Aldrich	H3663, clone HA-7	1:500	038M4810V
MAP2	Chemicon/ Millipore	AB5622	1:1000	2795016
nNOS	Abcam	ab1376	1:1000	GR3195323-9, GR3195323-11
TH	Immunostar	22941	1:2500	1552001, 1814001
**Secondary antibodies**				
Alexa Fluor® 488 AffiniPure Donkey Anti-Rabbit IgG	Jackson ImmunoResearch	711-545-152	1:1000	
CF®568-Donkey Anti-Mouse IgG	Biotium	20105	1:800	
CF®568-Donkey Anti-Goat IgG	Biotium	20106	1:800	
Alexa Fluor® 488 AffinaiPure Donkey Anti-Goat IgG	Jackson ImmunoResearch	705-545-147	1:1000	
Cy™5 AffiniPure Donkey Anti-Mouse IgG	Jackson ImmunoResearch	715-175-151	1:1000	
Alexa Fluor® 647 AffiniPure Donkey Anti-Guinea Pig IgG (H+L)	Jackson ImmunoResearch	706-605-148	1:1000	
Alexa Fluor® 647 AffiniPure Donkey Anti-Mouse IgG (H+L)	Jackson ImmunoResearch	715-605-150	1:1000000	
**Other**				
Alexa Fluor® 488 Streptavidin	Jackson ImmunoResearch	016-540-084	1:1000	
DAPI	Calbiochem	508741	1:2000	

Supplementary Table S4.

Name	Producer	Cat.No.	Vehicle
PD 128907 hydrochloride	Tocris	1243	distilled water
SB277011 dihydrochloride	Tocris	4207	DMSO
Rimonabant hydrochloride	Sigma	SML0800	DMSO
JZL 184	Tocris	3836	DMSO
Methyllycaconitine citrate	Tocris	1029	distilled water
Alexa647-α-bungarotoxin	Thermo Fischer	B35450	distilled water
2-Arachidonoyl Glycerol	Cayman Chemicals	62160	DMSO
WIN 55,212-2 mesylate	Tocris	1038	DMSO

Solvent	Producer	Cat.No.
DMSO	Sigma-Aldrich	D8418

(C) General considerations for designing PharmacoSTORM probes

The central idea of the PharmacoSTORM approach is based on the structural modification of already known and characterized ligands of the protein of interest. The objective of this modification is making the ligand detectable by STORM super-resolution imaging while keeping the key pharmacological properties unchanged. The fluorescent probes are capable to provide concentration-dependent high-resolution spatial information on the molecular mechanism of action of the original ligand. To achieve this goal, pharmacoprobe design involves the selection of the appropriate fluorescent dye amenable for STORM microscopy and the chemical strategy to link the dye to the ligand core.[S1–S7]

Selection of the STORM dye

In general, there are three key properties to select the appropriate dye including the excitation wavelength required to emit light, the brightness and *in vitro* and *in vivo* stability of the fluorophore. Specifically, a good STORM dye must meet several parameters such as photon yield per switching cycle, duty-cycle, photostability and number of switching cycles. The low background (autofluorescence) is particularly important in cellular, and especially in tissue imaging, hence longer wavelength excitation is beneficial. Furthermore, a detector with high quantum efficiency in the emission range is also essential. Cy5 and its analog Alexa Fluor 647 meet these requirements, and these dyes are the most widely used markers in STORM imaging.[S8,S9] Cyanine fluorophores are among the brightest fluorophores due to their very high extinction coefficients. Popularity of cyanines is also supported by their red/NIR emission, and by the enhanced water-solubility of derivatives equipped with sulfonyl groups (SulfoCy-type dyes).[S10–S12]

Selection of the ligand

The selection of the ligand depends heavily on the objective of the PharmacoSTORM experiments. Typical applications might involve investigations on the localization and function of the protein of interest as well as mechanism of action studies of the ligand.[S5,S13] In the first scenario, the selected ligand should show significant specificity to the target that requires extensive pharmacological characterization. For this purpose, best ligands show high affinity on the target with significant residence time to provide high target engagement. This feature should be coupled with selectivity against the paralogs of the target, which typically have remarkable sequence homology and/or similar 3D structure and binding pockets. In addition, similar properties are needed for the human and animal orthologs of the target protein in translational studies. Applications aiming *in vivo* studies would also require appropriate absorption, distribution, metabolism, and excretion - toxicity (ADMET) and pharmacokinetic properties.[S3,S14] Although these features are valid for both reversible and irreversible probes, the latter have specific advantages due to the covalent labeling of the protein of interest. Covalent probes might show higher affinity and selectivity towards the target protein, their residence time is

practically infinite and provides unique opportunity for investigating tissues *ex vivo* or *in vivo*.[S15] The other typical application of PharmacoSTORM probes would be the mechanistic investigation of known drugs. These studies should obviously use the unlabeled drug of interest; however, close structural analogs can also be considered as pharmacological tools. In this case, the ligand should undergo similar characterization as described above and its pharmacological profile should be compared to that of the drug of interest.[S16,S17]

Rational design of the PharmacoSTORM probe

The key objective of the design phase is to attach the selected fluorophore to the ligand while keeping the pharmacological profile of the resulting probe similar to that of the original ligand.[S3,S5,S7,S16,S18] Considering the size and shape limits of protein cavities and the specific interaction pattern needed for ligand recognition is an important step. Structural data from X-ray, cryo-EM or NMR studies of the protein-ligand complex are essential to find the best vectors to tag the fluorophore.[S19] In most cases, the selected fluorophores cannot enter into the active site that implicates the use of linker motifs between the ligand and the dye. A suitable linker helps to minimize the impact of the dye on the molecular recognition of the ligand.[S20] Potential growing vectors can be identified by analyzing the binding mode and the corresponding protein interactions of the ligand. The relative orientation of the ligand and the growing vector suggest the attachment point and the nature of the linker that connects the ligand with the fluorophore. The linker keeps the fluorophore out from the binding pocket – typically above the protein surface – that reduces its influence on the pharmacological profile of the ligand.[S20] In the absence of structural information on the complex formed between the selected ligand and the protein target of interest, computational studies might support the design process.[S20] Having the 3D structure of the protein co-crystallized with similar or other ligands (available in the PDB database, http://www.rcsb.org), docking calculations can be used predicting the binding mode of the ligand (see the design protocol of the CB_1 and MAGL probes below). Computational models of the target protein such as homology models might also be used for docking studies (see the design protocol of the D_3 probe below). These computational investigations can be performed using several available software and online analysis platforms including Schrödinger tools (e.g. Protein Preparation Wizard, LigPrep, Glide); or other databases and engines such as GOLD, ACEMD, OPM, CHARRM-GUI.[S21–S27] The availability of these platforms has significantly expanded in the last decade. Carefully analyzing the predicted binding mode and the interaction pattern with the binding site residues would provide feasible growing vectors for attaching the linker. These constructions can be re-docked to the binding site to validate the position and the required length of the linker. Finally, the linker positioned best to the ligand core is attached to the selected fluorophore to provide the PharmacoSTORM probe. For example, a linker length of seven atoms ($-(CH_2)_6-NH_2$) was necessary to reach above the protein surface in order to assure that the fluorophore won't clash sterically with the protein in case of the fluo-cannabinoid PharmacoSTORM probe CB_1 cannabinoid receptors.

Characterization of the PharmacoSTORM probe

Attaching the linker and the fluorophore to the ligand might substantially change the physicochemical properties of the original ligand. Since PharmacoSTORM applications need appropriate solubility, this property must be measured. If limited solubility found in this test, then the pharmacoprobe must be further improved by optimizing the linker or the dye. In case of the CB_1 and D_3 pharmacoprobes, the Cy5 fluorophore alone did not assure appropriate solubility. Therefore, the Sulfo-Cy5 dye had to be used. Next, the candidate pharmacoprobes should undergo a detailed pharmacological characterization that includes the investigation of their binding and functional properties. Finally, the most appropriate pharmacoprobe must be compared to the original ligand to validate its similar pharmacological profiles before using in PharmacoSTORM experiments.

(D) Synthesis of fluorescent pharmacoprobes for PharmacoSTORM experiments

Design and synthesis of ABP511 (fluo-cannabinoid), a fluorescent CB_1R agonist (1) for PharmacoSTORM experiments

Based on the recently published active-state crystal structure of the human CB_1 receptor[S28] (PDB ID: 5XRA, resolution: 2.8 Å) co-crystallized with the AM11542 full agonist (K_i = 0.11 nM) (Supplementary Fig. S1a) the hydroxymethyl substituent of the aromatic ring was identified as available for fluorescent labeling. This group is located at the entrance of the intrahelical site of the transmembrane bundle and points towards the extracellular space. Since our synthesis strategy was based on the selective modification of this position over the phenolic hydroxyl group, we selected hydroxy-cannabinol **2** having similarly high affinity (K_i = 0.1 nM)[S29] but offering a synthetically more tractable benzylic position for labeling. The corresponding hydroxy-cannabinol was first docked to the crystal structure (PDB ID: 5XRA). As expected, the aromatic character of the A ring did not change the binding mode significantly. The **2** hydroxy-cannabinol occupied the orthosteric binding site in an L-shape conformation similarly to AM11542 (Supplementary Fig. S1b) forming mainly hydrophobic and aromatic interactions with the receptor. The tricyclic core ring-system is stacked by aromatic π-π interactions with phenylalanine side chains (residues in light green; eg. $Phe268^{ECL2}$, $Phe379^{7.35}$, $Phe189^{3.25}$ and $Phe177^{2.64}$). The phenolic hydroxyl group forms hydrogen bond to $Ser383^{7.39}$. Similar to the A11542 structure, the long aliphatic sidechain of the hydroxy-cannabinol was accommodated by a hydrophobic channel defined located between helices III, V and VI (residues in light blue; eg. $Leu193^{3.29}$, $Val196^{3.32}$, $Tyr275^{5.39}$, $Leu276^{5.40}$, $Leu359^{6.51}$, $Met363^{6.55}$). In order to assure that the fluorophore reaches out of the membrane to the extracellular space, the cannabinol was equipped with a seven-membered linker. The attachment of the linker was presumed not to affect the binding mode (Supplementary Fig. S1c), whereas it enabled to get towards the extracellular entrance of the receptor between N-terminal loop and helix VII. Previous experience suggested cyanine fluorophores for STORM imaging.[S9] Based on preliminary solubility assessments Sulfo-Cy5 was chosen for labeling the linker coupled to the hydroxy-cannabinol. Our docking calculations confirmed that the binding mode of

the labeled compound was basically unchanged (Supplementary Fig. S1d) and the dye reached the extracellular space without interacting with the receptor.

Docking studies

The X-Ray structure of the active state human cannabinoid CB_1 receptor (PDB ID: 5XRA[S28]) was prepared with Schrödinger's Protein Preparation Wizard[S21] by adding missing side-chains, assignment of protonation states and minimization of the structure. The possible tautomers, conformations and protonation states of the ligand studies (**1**,**2**,**3**) were prepared with Schrödinger's LigPrep[S30] program. Ligands were docked into the binding site by Glide[S23,S31] in Single Precision mode using a 36x36x36 Å grid box, scaling of 0.50 Å VdW radii, and applying post-dock minimization. The hydrogen bond to Ser383$^{7.39}$ was constrained during all docking calculations.

Synthesis of ABP511 (1) (Supplementary Fig. S1e)

A convergent synthetic pathway was chosen for the synthesis of Sulfo-Cy5-labeled hydroxy-cannabinol 1-(7-((6-(((1-hydroxy-6,6-dimethyl-3-(2-methyloctan-2-yl)-6H-benzo[c]chromen-9-yl)methyl)amino)hexyl)amino)-7-oxoheptyl)-3,3-dimethyl-2-((1E,3E)-5-((E)-1,3,3-trimethyl-5-sulfoindolin-2-ylidene)penta-1,3-dien-1-yl)-3*H*-indol-1-ium-5-sulfonate (**1**) based on the Suzuki coupling of a bromobenzene and a benzeneboronic acid derivative.

At first, 4-bromo-3,5-dihydroxybenzoic acid was esterified in a Fischer-esterification in ethanol in the presence of cc. H_2SO_4 resulting in ester **4**.[S32] The phenolic hydroxy groups were protected by alkylation with benzyl bromide, and then **5** ester was reduced by $LiAlH_4$ resulting in the corresponding benzylalcohol (**5**) in excellent yield. The alcohol (**6**) was firstly turned to **7** benzylchloride in a nucleophilic substitution by thionyl-chloride, followed by the second analogous transformation by NaCN furnishing **8** benzyl cyanide in good yield. Double alkylation of the methylene group was accomplished using MeI forming 2-(3,5-bis(benzyloxy)-4-bromophenyl)-2-methylpropanenitrile (**9**).

The synthesis of the boronic acid intermediate was started from 4-hydroxymethylbenzoic acid. The free hydroxy group was sylilated by *tert*-butyldiphenylsilyl chloride,[S33] and the protected carboxylic acid (**10**) was transformed by oxalyl-chloride and DMF. The acyl chloride was not isolated, but reacted with diisopropylamine in the presence of DIPEA, resulting in **11** amide in good yield. The latter compound (**11**) was lithiated by *s*-BuLi and equipped with the boronic acid functional group by applying triisopropyl-borate. The intermediate (5-(((*t*-butyldiphenylsilyl)oxy)methyl)-2-(diisopropylcarbamoyl)phenyl)boronic acid (**12**) was isolated in a yield of 73%.

The two intermediates (**9** and **12**) were coupled in a Suzuki reaction in the presence of $Pd(dppf)_2Cl_2$ in a moderate yield. The nitrile group of **13** was turned to the corresponding aldehyde (**14**) using DIBAlH, followed by deprotection by tetrabutylammonium fluoride and a Wittig reaction with pentyltriphenylphosphonium bromide. The formed double bond and the benzylic protecting groups of **15** have been removed together via hydrogenation with Selcat Q6 Pd/C catalyst resulting in **16** resorcinol

derivative in an excellent yield. The tricyclic skeleton of **17** was formed by an intramolecular acylation in cc. AcOH. The **17** lactone was transformed to the **2** ether by ring-opening with MeMgI and the subsequent cyclization catalysed by 4-toluenesulfonic acid resulting in the hydroxy-cannabinol **2**. The benzylic alcohol group was transformed to chloride by thionyl-chloride, followed by the single alkylation of hexane-1,6-diamine linker in an acceptable yield for the two steps. The free amino group of the linker was coupled to the fluorescent dye applying Sulfo-Cy5-NHS ester.

Design and synthesis of ABP535 (fluo-cariprazine), a D_3R partial agonist for PharmacoSTORM experiments (18)

Cariprazine is a third-generation antipsychotic, marketed under the brand names Vraylar in the United States and Reagila in Europe. It acts primarily as a D_3 receptor and D_2 receptor partial agonist, with strong preference for the D_3 receptor. Our intention was to design a fluorescent labeled analogue with minimal structural modification that provides a comparable pharmacology profile to that of the marketed drug. The design of the appropriate cariprazine analogue needs the prediction of its binding mode in the D_3 receptor. Since cariprazine was found to be a D_3 preferring partial agonist,[S34] prediction of a reliable binding mode requires an active or active like conformation of the D_3 receptor. Unfortunately, however, the only available X-ray structure of the D_3 receptor is in inactive conformation co-crystallized with the antagonist eticlopride.[S35] Furthermore, other D_2-like receptors (D2[S36] and D4[S37]) are all in inactive form with bound antagonists (risperidone and nemonapride, respectively). The prediction of the cariprazine binding mode was therefore based on molecular docking into an active state homology model, which was built following the chimeric homology modelling approach used by GPCRdb.[S38] The binding mode was similar to the one we previously obtained in the inactive D_3 structure with molecular dynamics.[S39]

Cariprazine is well anchored in the binding site of the human dopamine D_3 receptor by two hydrogen bonds with Asp110$^{3.32}$ and Glu90$^{2.65}$ (Supplementary Fig. S4a), while further hydrophobic interactions (eg. with Val111$^{3.33}$, Leu89$^{2.64}$, Val86$^{2.61}$, Trp342$^{6.48}$, Phe345$^{6.51}$, Phe346$^{6.52}$ and Thr369$^{7.39}$) also contribute to the binding. The *N*,*N*-dimethylurea secondary binding motif of cariprazine is positioned towards the extracellular entrance of the receptor, however direct attachment of the dye at this point would likely interfere with the binding due to steric clashes with the top part of TM2 and TM7 as well as the extracellular loops. Therefore, we designed a linker to enlarge cariprazine towards the extracellular surface. Based on the obtained binding mode, the free terminal amine of the linker attains the surface of the receptor (Supplementary Fig. S4b), therefore the linker length is optimal to the attachment of the dye. This hypothesis is confirmed by the binding mode of the complex (Supplementary Fig. S4c). The dye is positioned above the receptor surface, while the cariprazine part maintains its original binding mode with the two anchoring hydrogen bonds to Asp110$^{3.32}$ and Glu90$^{2.65}$ suggesting this complex is suitable for imaging.

Docking studies

5HT$_{1B}$ – G$_{o1}$ structure complex (PDB ID : 6G79)[S40] was selected as the template for the active state D$_3$ homology model, and the D$_3$ loops from the inactive X-Ray structure were swapped in place of the 5HT1B loops in the main template.[S41] MODELLER[S41] was used to model residues without coordinates and the regions with steric clashes were remodeled with Schrödinger's Prime[S42]. Schrödinger's Protein Preparation Wizard[S21] was applied to add the missing hydrogen atoms and assign the protonation states. The ligands were prepared with Schrödinger's LigPrep[S30] program to generate stable protomer states and conformers. Next, the ligands were docked into the binding site applying Glide[S23,S31] single precision (SP) protocol using a 61x61x61 Å outer and 25x25x25 Å inner box size for the grid. The hydrogen bond formation with the conserved Asp110$^{3.32}$ was constrained during all docking and post-docking minimization was carried out for all poses.

Synthesis of the fluorescent cariprazine analogue (18) (Supplementary Fig. S4d)

The synthesis of the starting (1R,4R)-4-(2-(4-(2,3-dichlorophenyl)piperazin-1-yl)ethyl)cyclohexan-1-amine (**21**) was followed by the patent of Gedeon Richter Plc.[S43] Afterwards **21** was coupled to *tert*-butyl (3-(methylamino)propyl)carbamate (**22**) linker with triphosgene, and **23** was obtained in good yield. This was followed by removing the Boc protecting group by trifluoroacetic acid resulting in **20** amine. The free amino group of the linker was coupled to the fluorescent dye applying Sulfo-Cy5-NHS ester.

Structural basis for the D$_3$ preference of cariprazine over D$_2$ receptor (Supplementary Fig. S6)

We analyzed the structural differences that can contribute to the higher affinity of cariprazine (and potentially fluo-cariprazine (**18**)) towards D$_3$ over D$_2$ dopamine receptor subtype. We docked the ligand to the recently published active-state human D$_2$ (PDB ID: 6VMS) and D$_3$ (PDB ID: 7CMV) receptor structures. The stability of the binding modes was validated by 100 ns molecular dynamics simulations for each complex.[S44] Stable binding modes were then used to investigate the interaction pattern of cariprazine in both D$_2$R and D$_3$Rs. Overall, cariprazine binding modes are similar both in the D$_2$ and D$_3$ receptors, the ligand is anchored to the protein with strong and stable hydrogen bond with the conserved Asp3.32. Even though during docking the ligand was stabilized by a hydrogen bond with Glu2.65 in the D$_3$R, that interaction was lost during the molecular dynamics simulation. Instead, mainly water-mediated hydrogen bonds were formed with Ser182 in extracellular loop 2 (ECL2) and with Asn6.52. In the D$_2$R, the docking pose did not reveal any hydrogen bond in the extracellular vestibule, however during the simulation a new hydrogen bond was formed with Cys182$_{ECL2}$, and to a small extent Glu2.65 through a water bridge. Hydrophobic interactions with Phe6.51 and Phe6.52 add to the stabilization of both ligands. In the two active receptor structures, the top part of TM6 and especially His6.55 does not overlap well suggesting this residue as a potential selectivity driver. To detect potential interactions with His6.55 we measured the distance between the nitrogen atom of the histidine and the 2-chlorine atom of cariprazine that might form halogen bond. In the D$_2$R, only in 15% of the frames was the potential

halogen bond detectable (defined as smaller than 4 Å distance). Contrary, a halogen bond might stabilize the orthosteric binding motif in more than 50 % of the frames in the D_3R. In the D_2R, only hydrophobic interactions were observed suggesting a different orientation of the histidine residue compared to the dichlorophenyl piperazine binding motif of cariprazine. Although the experimental results are likely explained by a more complex phenomenon, this structural difference in the orthosteric binding pocket might play an important role in driving the D_3R selectivity of cariprazine.

Design and synthesis of DH-463, a fluorescent inhibitor of MAGL enzyme for PharmacoSTORM experiments

Docking studies

Molecular structure of DH-463 was drawn with Chemdraw19 and imported to ICM 3.8 (Molsoft). The crystal structure of MAGL was download form RCSB PDB (PDB ID: 6BQ0 http://doi.org/10.2210/pdb6BQ0/pdb) with a resolution of 2 Å and prepared with ICM 3.8 by deleting water molecules contained in the crystal structure, optimizing the hydrogens and other amino acids such as histidine, proline, glycine, cysteine etc. The active site of MAGL was defined using icmPocketFinder and the standard ICM VLS procedure was used to dock the covalent binding of inhibitor DH-463 to the active site Ser122. The poses with the lowest docking scores were manually examined and most reasonable pose for DH-463 was selected.

Synthesis of the fluorescent activity-based probe DH-463 (34) (Supplementary Fig. S3a)

As the first step of the synthesis, 2-bromoethane-1-ol (**24**) was transformed to **25** azide with sodium azide, followed by the mesylation of the hydroxy group leading to mesylate **26**. Alkylating 4-hydroxybenzaldehyde with **26** led to 4-(2-azidoethoxy)benzaldehyde (**27**) that was substituted in a Grignard reaction to obtain (4-(2-azidoethoxy)phenyl)(4-chlorophenyl)methanol (**28**) with a good yield. The free hydroxy group was changed to chlorine using $SOCl_2$, followed by the *N*-alkylation of *N*-Boc-piperazine. The Boc protecting group of **30** was removed by HCl in dioxane leading to **31** that was acylated by triphosgene introducing the warhead for covalent labelling. **DH-463** (**34**) was produced in the click reaction of **32** azide and Cy5 alkyne (**33**).

(E) Supplementary methods

General synthetic methods

All reactions were performed using oven- or flame-dried glassware and dry solvents. Reagents were purchased from Sigma Aldrich, Acros and Merck, and used without further purification unless noted otherwise. All moisture-sensitive reactions were performed under an argon atmosphere. Traces of water were removed from starting compounds by co-evaporation with toluene. ^{1}H- ^{13}C- and APT NMR spectra were recorded in DMSO-d_6, $CDCl_3$ or CD_3OD solution at room temperature, on a Varian Unity Inova 500 spectrometer (500 and 125 MHz for ^{1}H and ^{13}C or APT NMR spectra, respectively). Otherwise, a Bruker AV 400 MHz spectrometer at 400 (^{1}H) and 101 (^{13}C) MHz, or on a Bruker DMX-600 spectrometer 600 (^{1}H) and 150 (^{13}C) MHz using $CDCl_3$, or CD_3OD solvent was used. Deuterium signal of the solvent was set as the lock and TMS as the internal standard. Chemical shifts (δ) and coupling constants (*J*) are given in ppm and Hz, respectively. Data are reported as follows: chemical shifts (δ), multiplicity (s = singlet, d = doublet, dd = double doublet, td = triple doublet, t = triplet, m = multiplet, br = broad), coupling constants J (Hz), and integration. HPLC-MS measurements were performed using (A) a Shimadzu LCMS-2020 device equipped with a Reprospher 100 C18 (5 µm; 100x3 mm) column and positive-negative double ion source (DUIS±) with a quadrupole MS analysator in a range of 50-1500 m/z. The samples were eluted with gradient elution using eluent A (0.1% formic acid in water) and eluent B (0.1% formic acid in acetonitrile). Flow rate was set to 1.5 mL/min. The initial condition was 5% B eluent, followed by a linear gradient to 100% B eluent by 1.5 min, from 1.5 to 4.0 min 100% B eluent was retained; and from 4.0 to 4.5 min back to initial condition with 5% B eluent and retained to 5 min. The column temperature was kept at room temperature, and the injection volume was 1-10 µL. HPLC-MS measurements were performed using (B) a Finnigan Surveyor HPLC system with a Gemmi C18 50x4.60 mm column (detection at 200-600 nm), coupled to a Finnigan LCQ Adantage Max mass spectrometer with ESI. The applied buffers were H2O, MeCN and 1.0% TFA in H2O (0.1% TFA end concentration). High-resolution mass spectra (HRMS) were recorded by direct injection (2 µL of a 2 µM solution in water/acetonitrile 50/50 (v/v) and 0.1% formic acid) on a mass spectrometer (Thermo Finnigan LTQ orbitrap) equipped with an electrospray ion source in positive mode (source voltage 3.5 kV, sheath gas flow 10, capillary temperature 250 °C) with resolution R = 60000 at m/z 400 (mass range m/z = 150-2000) and dioctylphthalate (m/z = 391.28428) as a "lock mass". The high-resolution mass spectrometer was calibrated prior to measurements with a calibration mixture (Thermo Finnigan). Purity of compounds was assessed by HPLC with UV detection at 215 and 254 nm and by NMR; all starting compounds are known, purchased or synthetically feasible and >95% pure. Flash chromatography was performed using SiliCycle silica gel type SilicaFlash P60 (230 – 400 mesh). TLC analysis was performed on Merck silica gel 60/Kieselguhr F254, 0.25 mm. Compounds were visualized using either Seebach's reagent (a mixture of phosphomolybdic acid (25 g), cerium (IV) sulfate (7.5 g), H_2O (500 mL) and H_2SO_4 (25 mL)) or a $KMnO_4$ stain (K_2CO_3 (40 g), $KMnO_4$ (6 g), H_2O (600 mL) and 10% NaOH (5 mL)).

(F) Synthesis of probes

Experimental procedures for the synthesis of ABP511, the fluo-cannabinoid (1)

Synthesis of 3,5-bis(benzyloxy)-4-bromobenzoate (5)

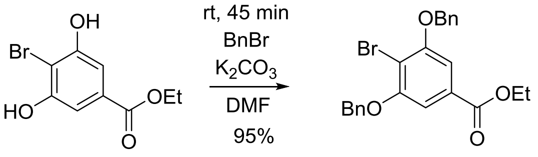

4 **5**

In a round-bottom flask, ethyl 4-bromo-3,5-dihydroxybenzoate (1.00 g, 3.83 mmol) was dissolved in 40 mL DMF, followed by the addition of K_2CO_3 (2.65 g, 5 equiv., 19.15 mmol) and benzyl-bromide (1.5 mL, 3.3 equiv., 12.60 mmol). The reaction mixture was stirred at room temperature for 45 min. Ethyl acetate (100 mL) was added, and the mixture was washed with water (3x30 mL) and brine (30 mL). The organic layer was separated and dried over $MgSO_4$. The crude residue was purified by flash chromatography on silica with *n*-hexane and ethyl acetate as eluents to give ethyl 3,5-bis(benzyloxy)-4-bromobenzoate as a white solid (1.60 g, 3.64 mmol, 95%).

^{1}H NMR (500 MHz, $CDCl_3$) δ 7.52 (d, *J* = 7.5 Hz, 4H), 7.40 (t, *J* = 7.5 Hz, 4H), 7.36-7.31 (m, 4H), 5.22 (s, 1H), 4.38 (q, *J* = 7.1 Hz, 2H), 1.40 (t, *J* = 7.1 Hz, 3H) ppm. ^{13}C NMR (125 MHz, $CDCl_3$) δ 165.8 (C=O), 156.3 (C), 136.2 (C), 130.4 (C), 128.6 (CH), 128.0 (CH), 127.1 (CH), 108.3 (C), 107.4 (CH), 71.1 (CH2), 61.4 (CH2), 14.3 (CH3) ppm. HRMS (ESI): $(M+H)^+$ calcd. for $C_{23}H_{22}BrO_4$, 441.0695; found, 441.0700.

Synthesis of (((2-bromo-5-(chloromethyl)-1,3-phenylene)bis(oxy))bis(methylene))dibenzene (7)[S45]

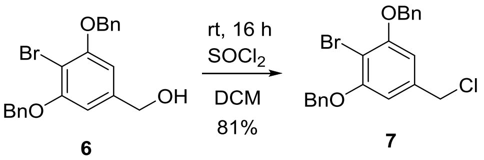

6 **7**

In a round bottom flask, (3,5-bis(benzyloxy)-4-bromophenyl)methanol (1.19 g, 2.98 mmol) was dissolved in 30 mL dry dichloromethane and thionyl chloride (0.33 mL, 4.47 mmol, 1.5 equiv.) was added in 20 mL DCM dropwise at 0 °C. The reaction mixture was stirred at room temperature overnight. The solvent and the excess of the reagent was removed, and the crude product was evaporated to silica, and purified by flash chromatography with *n*-hexane and ethyl acetate as eluents. The pure product was a white crystal (1.00 g, 81%).

^{1}H NMR (500 MHz, DMSO-d_6) δ 7.49 (d, *J* = 7.32 Hz, 4H), 7.41 (t, *J* = 7.32 Hz, 4H), 7.34 (t, *J* = 7.32 Hz, 2H), 6.96 (s, 2H), 5.19 (s, 4H), 4.69 (s, 2H) ppm. ^{13}C NMR (125 MHz, $CDCl_3$) δ 152.5 (C), 133.8 (C), 132.4 (C), 124.6 (CH), 124.0 (CH), 123.09 (CH), 123.06 (CH), 103.0 (C), 67.1 (CH_2), 42.2 (CH_2) ppm. HRMS (ESI): $(M+H)^+$ calcd. for $C_{21}H_{19}BrClO_2$, 417.0251; found, 417.0296.

*Synthesis of 2-(3,5-bis(benzyloxy)-4-bromophenyl)acetonitrile (**8**)*

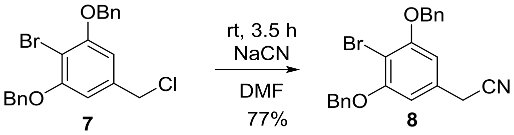

In a round bottom flask, (((2-bromo-5-(chloromethyl)-1,3-phenylene)bis(oxy))bis(methylene)) dibenzene (0.80 g, 1.92 mmol) was dissolved in 20 mL DMF, and NaCN (0.19 g, 3.84 mmol, 2 equiv.) was added. The reaction mixture was stirred at room temperature for 3.5 h, quenched with 30 ml water, and washed with ethyl acetate (2x30 mL). The organic phase was dried over $MgSO_4$, and the solvent was evaporated. The crude product was purified by flash chromatography with *n*-hexane and ethyl acetate as eluents resulting in 0.60 g white crystals (77%).

^{1}H NMR (500 MHz, $CDCl_3$) δ 7.49 (d, *J* = 7.5 Hz, 4H), 7.40 (t, *J* = 7.5 Hz, 4H), 7.33 (t, *J* = 7.3 Hz, 2H), 6.57 (s, 2H), 5.17 (s, 4H), 3.65 (s, 2H) ppm. ^{13}C NMR (125 MHz, $CDCl_3$) δ 156.8 (CN), 136.1 (C), 130.2 (C), 128.6 (CH), 128.1 (CH), 127.0 (CH), 117.2 (C), 106.3 (CH), 102.4 (C), 71.1 (CH_2), 23.8 (CH_2) ppm. HRMS (ESI): $(M+H)^+$ calcd. for $C_{22}H_{19}BrNO_2$, 408.0593; found, 417.0592.

*Synthesis of 2-(3,5-bis(benzyloxy)-4-bromophenyl)-2-methylpropanenitrile (**9**)*

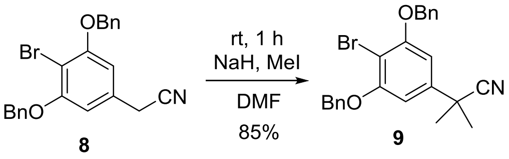

In a round bottom flask, NaH (0.823 g, 20.58 mmol, 3 equiv.) was suspended in 40 mL DMF, and the solution of 2-(3,5-bis(benzyloxy)-4-bromophenyl)acetonitrile (2.8 g, 6.86 mmol) and MeI (1.28 mL, 20.58 mmol, 3 equiv.) in 30 mL DMF was added dropwise. The reaction was stirred at room temperature for 1 h, then quenched with 80 mL concd. NH_4Cl and 40 mL water. The mixture was extracted with ethyl acetate (100 mL), the organic phase was separated and dried over Na_2SO_4. The crude yellowish crystals were triturated with 100 mL diethyl ether, filtered, and washed with 100 mL diethyl ether resulting in the product as white crystals (2.42 g). The mother liquor was purified by flash chromatography with n-hexane and ethyl acetate as eluents giving 0.54 g product.

^{1}H NMR (500 MHz, $CDCl_3$) δ 7.50 (d, *J* = 7.5 Hz, 4H), 7.40 (t, *J* = 7.5 Hz, 4H), 7.33 (t, *J* = 7.3 Hz, 4H), 6.71 (s, 2H), 5.19 (s, 4H), 1.64 (s, 6H) ppm. ^{13}C NMR (125 MHz, $CDCl_3$) δ 156.5 (CN), 141.9 (C), 136.2 (C), 128.6 (CH), 128.1 (CH), 127.2 (CH), 123.0 (C), 104.2 (CH), 102.4 (C), 71.3 (CH_2), 37.4 (C), 29.0 (CH_3) ppm. HRMS (ESI): $(M+H)^+$ calcd. for $C_{24}H_{23}BrNO_2$, 436.0906; found, 436.0917.

*Synthesis of 4-(((t-butyldiphenylsilyl)oxy)methyl)-N,N-diisopropylbenzamide (**11**)*

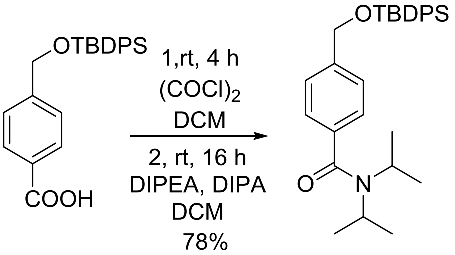

10 **11**

In a round bottom flask, 4-(((*t*-butyldiphenylsilyl)oxy)methyl)benzoic acid (23.0 g, 59.0 mmol) was dissolved in 350 mL DCM followed by the dropwise addition of oxalyl chloride (15 mL, 177.0 mmol, 3.0 equiv.). The reaction mixture was stirred for 4 h at room temperature. The solvent and the excess of the reagent was evaporated, and the residue was dissolved in 200 mL DCM. The solution of ethyl-diisopropylamine (108 mmol, 2.0 equiv) and diisopropylamine (108 mmol, 2.0 equiv.) in 200 mL DCM was added dropwise and was stirred overnight. The reaction was quenched and washed with water (2x250 mL) the organic phase was dried over $MgSO_4$, and the crude product was purified by flash chromatography using *n*-hexane and dichloromethane as eluents resulting in 21.80 g (78%) of the product as a yellow oil.

^{1}H NMR (500 MHz, $CDCl_3$) δ 7.70-7.68 (m, 4H), 7.43-7.41 (m, 2H), 7.39-7.34 (m, 6H), 7.29-7.26 (m, 2H), 4.79 (s, 2H), 1.46-1.34 (m, 2H), 1.29-1.25 (m, 9H), 1.11 (s, 12H) ppm.^{13}C NMR (125 MHz, $CDCl_3$) δ 171.1 (CO), 141.6 (C), 137.5 (C), 135.5 (CH), 134.8 (CH), 133.4 (C), 129.7 (CH), 129.5 (CH), 127.7 (CH), 127.6 (CH), 125.9 (CH), 125.6 (CH), 65.24 (CH_2), 26.8 (CH_3), 26.6 (CH), 20.8 (CH_3), 19.30 (C) ppm. HRMS (ESI): $(M+H)^+$ calcd. for $C_{30}H_{40}NO_2Si$, 474.2822; found, 474.2813.

*Synthesis of (5-(((t-butyldiphenylsilyl)oxy)methyl)-2-(diisopropylcarbamoyl)phenyl)boronic acid (**12**)*

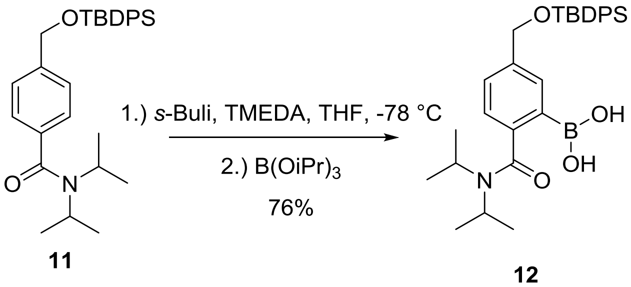

11 **12**

In a round bottom flask, flushed with Ar, 80 mL dry THF was added together with 2.84 mL TMEDA (19 mmol, 2 equiv.), followed by the addition of *s*-BuLi (19 mmol, 2 equiv., 13.5 mL of 1.4 M in hexane) at -78 °C. The mixture was stirred for 30 min, and 4-(((*t*-butyldiphenylsilyl)oxy)methyl)-N,N-diisopropylbenzamide (4.5 g, 9.5 mmol) was added dropwise in 40 mL dry THF. After 30 min stirring at -78 °C triisopropyl borate (6.58 mL, 28.5 mmol, 3 equiv.) was added dropwise in 40 mL THF. The

reaction mixture was stirred overnight at room temperature, then 25 mL 5% HCl solution was added at 0 °C and the pH was set of 6.5-7. The mixture was extracted with 250 mL DCM, washed with 100 mL water and 100 mL brine together with 100 mL water. The organic phase was separated, dried over $MgSO_4$, and evaporated to silica. Flash chromatography was performed using DCM and methanol as eluents resulting in 3.74 g (76%) of the product (two rotamers) as a yellow oil.

^{1}H NMR (500 MHz, $CDCl_3$) δ 7.85-7.78 (m, 1H), 7.73-7.65 (m, 8H), 7.50 (d, *J* = 7.4 Hz, 1H), 7.48-7.44 (m, 1H), 7.42 (d, *J* = 7.1 Hz, 2H), 7.40-7.35 (m, 6H), 7.33 (d, *J* = 7.2 Hz, 3H), 7.32-7.29 (m, 1H), 7.19-7.12 (m, 1H), 4.81 (s, 2H), 4.79 (s, 2H), 4.15-4.03 (m, 1H), 3.89-3.77 (m, 1H), 3.74-3.63 (m, 1H), 3.60-3.49 (m, 1H), 3.40-3.31 (m, 1H), 1.63-1.54 (m, 6H), 1.32-1.24 (m, 7H), 1.24-1.16 (m, 4H), 1.12 (s, 15H), 1.06 (s, 11H), 1.00-0.92 (m, 2H), 0.92-0.82 (m, 3H) ppm. ^{13}C NMR (125 MHz, $CDCl_3$) δ 171.4 (CO), 135.6 (CH), 135.5 (CH), 133.5 (C), 133.4 (C), 129.7 (CH), 129.6 (CH), 127.71 (CH), 127.66 (CH), 127.0 (CH), 126.7 (CH), 124.9 (CH), 124.4 (CH), 65.4 (CH_2), 65.2 (CH_2), 29.7 (C), 26.9 (CH_3), 20.5 (CH_3), 20.4 (CH), 20.2 (CH), 19.32 (C), 19.27 (C) ppm. HRMS (ESI): $(M+H)^+$ calcd. for $C_{30}H_{41}BNO_4Si$, 518.2892; found, 518.2887.

*Synthesis of 2',6'-bis(benzyloxy)-5-(((t-butyldiphenylsilyl)oxy)methyl)-4'-(2-cyanopropan-2-yl)-N,N-diisopropyl-[1,1'-biphenyl]-2-carboxamide (**13**)*

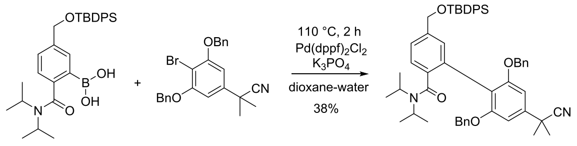

In a microwave vial, (5-(((*t*-butyldiphenylsilyl)oxy)methyl)-2-(diisopropylcarbamoyl)phenyl)boronic acid (1 g, 1.93 mmol, 1.3 equiv.) and 2-(3,5-bis(benzyloxy)-4-bromophenyl)-2-methylpropanenitrile (0.65 g, 1.48 mmol) was dissolved in a mixture of 15 mL dioxane and 2 mL water, followed by the addition of [1,1′-bis(diphenylphosphino)ferrocene]dichloropalladium(II) (110 mg, 0.15 mmol, 0.1 equiv.) and K_3PO_4 (0.94 g, 4.44 mmol, 3 equiv.). The reaction mixture was placed to a MW reactor and held at 110 °C for 1 h. Then another 110 mg of Pd(dppf)$_2$Cl$_2$ was added, and the reaction was heated in the MW reactor for an additional 1 h at 110 °C. The mixture was evaporated to silica and was purified by flash chromatography using *n*-hexane and ethyl acetate as eluents resulting in 1.48 g (44%) of the product as a yellowish solid.

^{1}H NMR (500 MHz, $CDCl_3$) δ7.70 (d, *J* = 7.32 Hz**,** 3H), 7.44-7.39 (m, 3H), 7.35-7.32 (m, 6H), 7.23-7.19 (m, 6H), 7.10 (bs, 1H), 6.76 (s, 1H), 6.62 (s, 1H), 5.03 (s, 1H), 5.00 (s, 2H), 4.81 (s, 2H), 3.77-3.71 (m, 1H), 3.26-3.22 (m, 1H), 1.62 (s, 7H), 1.50-1.44 (m, 2H), 1.33-1.24 (m, 4H), 1.09 (s, 12H), 0.91-0.85 (m, 6H), 0.66-0.57 (m, 2H) ppm. ^{13}C NMR (125 MHz, $CDCl_3$) δ 173.9 (CO), 156.6 (C), 155.2 (C), 142.7 (C), 140.6 (C), 138.6 (C), 135.6 (C), 134.8 (C), 130.8 (C), 129.7 (C), 129.6 (C), 129.0 (CH), 128.33 (CH), 128.26 (CH), 127.7 (CH), 126.5 (CH), 125.0 (CH), 124.3 (CN), 70.4 (CH_2), 65.3 (CH_2), 50.2

(CH), 37.5 (C), 29.1 (CH_3), 26.9 (CH_3), 26.5 (CH_3), 19.3 (CH_3) ppm. HRMS (ESI): $(M+H)^+$ calcd. for $C_{54}H_{61}N_2O_4Si$, 830.4395; found, 830.4374.

*Synthesis of (E)-2',6'-bis(benzyloxy)-5-(hydroxymethyl)-N,N-diisopropyl-4'-(2-methyloct-3-en-2-yl)-[1,1'-biphenyl]-2-carboxamide (**15**)*

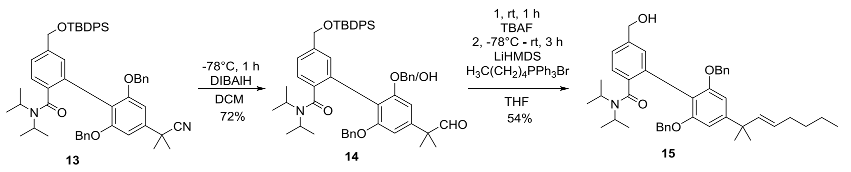

In a dry round bottom flask, 2.1 g 2',6'-bis(benzyloxy)-5-(((*t*-butyldiphenylsilyl)oxy)methyl)-4'-(2-cyanopropan-2-yl)-N,N-diisopropyl-[1,1'-biphenyl]-2-carboxamide (2.5 mmol) was dissolved in 20 mL DCM distilled from CaH_2, and 3.75 mL DIBALH (1M in DCM, 3.75 mmol, 1.5 equiv.) was added dropwise at -78 °C. Additional reagent (3.75 mL and 1.9 mL) was added after 40 min and 80 min. The reaction mixture was quenched with 20 mL 10% aqueous solution of Seignette-salt, and let the temperature to room temperature. The suspension was filtered through celite, washed with 50 mL DCM and 30 mL ethyl acetate. The organic phase was separated, dried over $MgSO_4$, and the solvent was evaporated resulting in the mixture of the product (73%) and a debenzylated derivative (20%). The mixture was not separated but taken to the next reaction step without purification. In a round bottom flask 1.89 g 2',6'-bis(benzyloxy)-5-(((*t*-butyldiphenylsilyl)oxy)methyl)-N,N-diisopropyl-4'-(2-methyl-1-oxopropan-2-yl)-[1,1'-biphenyl]-2-carboxamide and it's debenzylated derivative (2.32 mmol) was dissolved in 50 mL THF, and 1.46 g TBAF.3H_2O (4.64 mmol, 2 equiv.) was added. After 1 h the mixture was evaporated to silica and let through a short silica column using *n*-hexane, dichloromethane and methanol in order to separate the aldehydes from the residue of the protecting group. For the next reaction step pentyltriphenylphosphonium bromide (1.87 g, 4.53 mmol, 3 equiv.) was suspended in 15 mL THF under nitrogen at 0 °C, and LiHMDS (4.68 mL, 1M in THF, 4.68 mmol, 3.1 equiv.) was added dropwise. After stirring for 1 h, it was cooled to -78 °C, and the aldehyde mixture was added dropwise in 15 mL THF. The reaction was stirred at room temperature for 3 h, then quenched with 30 mL cc NH_4Cl, and extracted with 2x50 mL diethyl ether. The organic phase was separated, dried over Na_2SO_4, the solvent was evaporated, and the crude was purified by flash chromatography using *n*-hexane and ethyl acetate as the eluents.

^{1}H NMR (500 MHz, $CDCl_3$) δ 7.36-7.30 (m, 4H), 7.29-7.20 (m, 6H), 7.17 (d, *J* = 7.2 Hz, 1H), 7.14 (d, *J* = 7.1 Hz, 2H), 6.63 (d, *J* = 7.0 Hz, 2H), 5.60-5.54 (m, 1H), 5.29-5.22 (m, 1H), 5.20-5.14 (m, 1H), 5.01-4.94 (m, 3H), 4.66 (s, 2H), 3.77-3.67 (m, 1H), 3.28-3.19 (m, 1H), 1.55 (dd, *J* = 13.2, 6.1 Hz, 2H), 1.49 (d, *J* = 6.6 Hz, 3H), 1.32 (d, *J* = 3.1 Hz, 7H), 1.15 (d, *J* = 6.6 Hz, 3H), 1.14-1.10 (m, 4H), 0.85 (d, *J* = 6.5 Hz, 3H), 0.79 (t, *J* = 6.9 Hz, 3H), 0.58 (d, *J* = 6.4 Hz, 3H) ppm. ^{13}C NMR (125 MHz, $CDCl_3$) δ 170.2 (CO), 152.5 (C), 140.3(C), 138.6 (CH), 138.5 (C), 131.8 (CH), 131.7 (C), 130.5 (C), 128.3 (CH),

128.1 (CH), 127.5 (C), 127.1 (CH), 127.0 (C), 126.6 (CH), 125.7 (CH), 115.8 (CH), 70.8 (CH_2), 69.9 (CH_2), 65.1 (CH_2), 50.1 (CH), 45.3 (C), 40.4 (CH_2), 31.6 (CH_3), 28.3 (CH_3), 22.3 (CH_2), 20.7 (CH_2), 20.14 (CH_3), 20.10 (CH_3), 14.0 (CH_3) ppm. HRMS (ESI): $(M+H)^+$ calcd. for $C_{43}H_{54}NO_4$, 648.4047; found, 648.4046.

*Synthesis of 2',6'-dihydroxy-5-(hydroxymethyl)-N,N-diisopropyl-4'-(2-methyloctan-2-yl)-[1,1'-biphenyl]-2-carboxamide (**16**)*

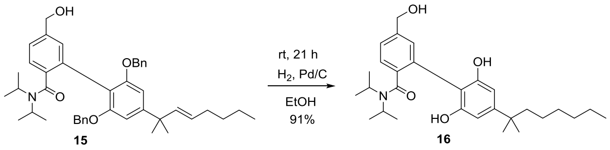

In an autoclave, 240 mg (E)-2',6'-bis(benzyloxy)-5-(hydroxymethyl)-N,N-diisopropyl-4'-(2-methyloct-3-en-2-yl)-[1,1'-biphenyl]-2-carboxamide (0.37 mmol) was dissolved in 15 mL EtOH, and 100 mg Selcat Q6 Pd/C was added. The reactor was flushed with nitrogen and hydrogen, and filled with 10 bar H_2, and stirred vigorously overnight. The suspension was filtered through celite, and the crude was purified by flash chromatography using *n*-hexane and ethyl acetate as eluents resulting in 157 mg (91%) of the product as a white solid.

^{1}H NMR (500 MHz, CD_3OD) δ 7.39 (d, J = 7.7 Hz, 1H), 7.28 (s, 1H), 7.25 (d, J = 7.8 Hz, 1H), 6.47 (s, 1H), 6.42 (s, 1H), 4.79 (s, 2H), 4.65 (s, 2H), 3.85-3.76 (m, 1H), 3.43-3.35 (m, 1H), 1.60-1.53 (m, 2H), 1.46 (d, J = 6.7 Hz, 3H), 1.31-1.18 (m, 13H), 1.12 (d, J = 6.7 Hz, 3H), 1.08 (s, 2H), 1.02 (d, J = 6.6 Hz, 3H), 0.87 (t, J = 7.0 Hz, 3H), 0.83 (d, J = 6.5 Hz, 3H) ppm. ^{13}C NMR (125 MHz, $CDCl_3$) δ 172.5 (C), 154.9 (C), 154.6 (C), 151.3 (C), 141.6 (C), 137.8 (C), 131.4 (C), 130.8 (CH), 125.7 (CH), 125.0 (CH), 113.2 (C), 106.6 (CH), 105.2 (CH), 63.5 (C), 51.0 (CH), 45.5 (CH_3), 44.3 (CH_2), 37.1 (CH_2), 31.6 (CH_2), 29.8 (CH_2), 28.2 (CH_3), 28.1 (CH_3), 24.5 (CH_2), 22.3 (CH_2), 19.5 (CH_3), 19.1 (CH_3), 18.9 (CH_3), 13.0 (CH_3) ppm. HRMS (ESI): $(M+H)^+$ calcd. for $C_{29}H_{44}NO_4$, 470.3264; found, 470.3271.

*Synthesis of 1-hydroxy-9-(hydroxymethyl)-3-(2-methyloctan-2-yl)-6H-benzo[c]chromen-6-one (**17**)*[S46]

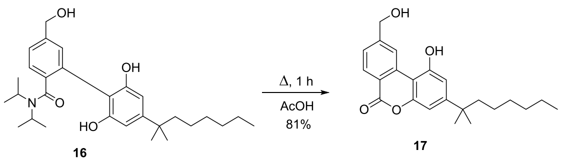

In a round bottom flask, 2',6'-dihydroxy-5-(hydroxymethyl)-N,N-diisopropyl-4'-(2-methyloctan-2-yl)-[1,1'-biphenyl]-2-carboxamide (180 mg, 0.38 mmol) was dissolved in 10 mL cc. AcOH, and refluxed for 1 h. At 0 °C 15 mL water was added, and extracted with 25 mL diethyl ether. The organic phase was washed with 10 mL water, 10 mL cc $NaHCO_3$, 10 mL brine, dried over $MgSO_4$, evaporated, and the crude was purified by flash chromatography using *n*-hexane and ethyl acetate as the eluents resulting in 114 mg (81%) of the product, as a white solid.

^{1}H NMR (500 MHz, $CDCl_3$) δ 9.00 (s, 1H), 8.21 (d, *J* = 8.1 Hz, 1H), 7.34 (d, *J* = 7.9 Hz, 1H), 6.81 (d, *J* = 2.5 Hz, 2H), 4.84 (s, 2H), 1.57-1.51 (m, 2H), 1.25 (s, 6H), 1.21-1.10 (m, 7H), 1.08-1.00 (m, 2H), 0.79 (t, *J* = 7.0 Hz, 3H) ppm. ^{13}C NMR (125 MHz, $CDCl_3$) δ 162.4 (CO), 154.9 (C), 153.2 (C), 152.3 (C), 147.6 (CH), 135.3 (C), 130.1 (CH), 125.4 (CH), 124.8 (C), 119.2 (C), 110.2 (CH), 107.1 (CH), 104.4 (C), 64.9 (CH_2), 44.3 (CH_2), 37.9 (CH), 31.7 (CH_2), 29.9 (CH_3), 28.5 (CH_2), 24.7 (CH_2), 22.6 (CH_2), 14.0 (CH_3) ppm. HRMS (ESI): $(M+H)^+$ calcd. for $C_{23}H_{29}O_4$, 369.2060; found, 369.2059.

*Synthesis of 1-(7-((6-(((1-hydroxy-6,6-dimethyl-3-(2-methyloctan-2-yl)-6H-benzo[c]chromen-9-yl)methyl)amino)hexyl)amino)-7-oxoheptyl)-3,3-dimethyl-2-((1E,3E)-5-((E)-1,3,3-trimethyl-5-sulfoindolin-2-ylidene)penta-1,3-dien-1-yl)-3H-indol-1-ium-5-sulfonate (**1**)*

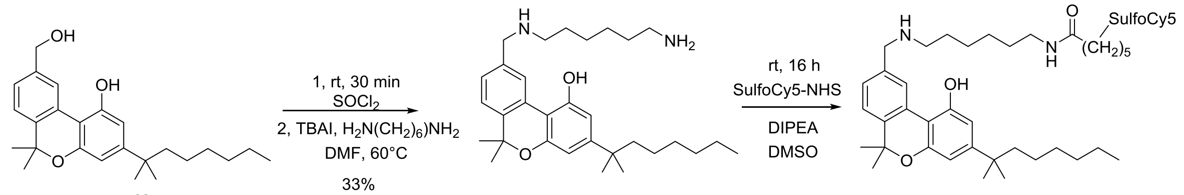

In a round bottom flask 9-(hydroxymethyl)-6,6-dimethyl-3-(2-methyloctan-2-yl)-6H-benzo[c]chromen-1-ol (17 mg, 44 umol) was dissolved in 2 mL dry DCM, and thionyl chloride (6.4 uL, 88 umol, 2 equiv.) was added dropwise in 1 mL DCM at room temperature. The mixture was stirred for 30 min, the solvent and the excess of the reagent was evaporated. The crude was dissolved in 1 mL DMF, and potassium carbonate (9.1 mg, 66 umol, 1.5 equiv.) was added together with 1,6-diaminohexane (15.3 mg, 132 umol, 3 equiv.), and stirred overnight. The reaction mixture was purified by preparative HPLC, resulting in the product as a colourless film (7 mg, 33%) that was dissolved in 1 mL DMSO, followed by the addition of ethyl-diisopropylamine (3.3 uL, 19.2 umol, 1.3 equiv.) and Sulfo-Cy5 NHS ester (5 mg, 6.4 umol, 0.44 equiv.). The reaction mixture was stirred in dark at room temperature overnight, and was purified by preparative HPLC resulting the product as a blue solid (5 mg, 31%).

^{1}H NMR (500 MHz, CD_3OD) δ 8.92-8.55 (m, 1H), 8.46-8.17 (m, 2H), 7.99-7.79 (m, 4H), 7.65-7.18 (m, 4H), 6.89-6.51 (m, 2H), 6.49-6.41 (m, 1H), 6.41-6.11 (m, 1H), 4.48-3.94 (m, 3H), 3.80-3.51 (m, 4H), 3.34 (s, 3H), 3.19-2.95 (m, 3H), 2.36-1.95 (m, 3H), 1.84 (s, 1H), 1.74 (s, 15H), 1.57 (s, 9H), 1.48-1.03 (m, 27H), 0.83 (t, *J* = 6.4 Hz, 3H) ppm. ^{13}C NMR (125 MHz, CD_3OD) δ 174.8, 154.8, 154.0, 150.9, 144.1, 143.4, 141.8, 141.1, 136.6, 128.0, 126.8, 126.7, 126.63, 126.57, 126.3, 121.9, 119.90, 119.85, 118.7, 110.3, 110.2, 108.0, 107.1, 106.7 104.1, 103.7, 76.7, 56.8, 53.0, 50.6, 49.2, 49.1, 44.1, 43.4, 39.1, 37.0, 35.9, 35.0, 33.1, 31.8, 31.5, 30.4, 29.7, 27.9, 26.8, 26.7, 26.5, 26.3, 26.1, 25.8, 25.7, 25.0, 24.4, 22.2, 20.2, 12.9 ppm. HRMS (ESI): $(M+2H)^{2+}$ calcd. for $C_{63}H_{85}N_4O_9S_2$, 553.2912; found 553.2887.

Experimental procedures for the synthesis of ABP535, the fluo-cariprazine (18)

*Synthesis of tert-butyl N-{3-[methyl({[(1R,4R)-4-{2-[4-(2,3-dichlorophenyl)piperazin-1-yl]ethyl}cyclohexyl]carbamoyl})amino]propyl}carbamate (**23**)*

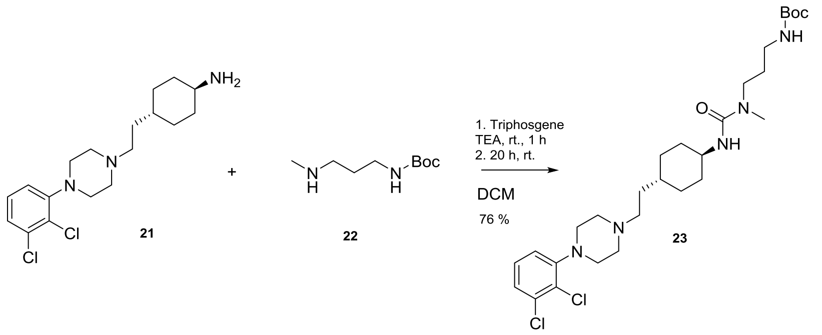

In a round bottom flask, (1R,4R)-4-{2-[4-(2,3-dichlorophenyl)piperazin-1-yl]ethyl}cyclohexane-1-amine (103 mg, 0.29 mmol) was dissolved in 15 ml of DCM. TEA (167 µl, 1.2 mmol) was added to the reaction mixture. Triphosgene (30 mg, 0.10 mmol, 0.83 equiv.) was dissolved in 2 ml of DCM and dropped to the previous mixture. The reaction mixture was stirred at room temperature for 1 hour. On the next step tert-butyl N-[3-(methylamino)propyl]carbamate (225 mg, 1.2 mmol, 1.0 equiv.) and TEA (167 µl, 1.2 mmol, 1.0 equiv.) were added. The reaction was continued for 20 hours at room temperature. The reaction mixture was filtered, and the filtrate was washed with water. The phases were separated, and the organic phase was dried over Na_2SO_4. The crude material was purified by flash chromatography, eluting with DCM: MeOH (0-5%). 125 mg (76 %) white solid was obtained. HRMS (ESI) $(M+H)^+$ calcd for $C_{28}H_{46}N_5O_3Cl_2$, 570.2972; found 570,2974.

^{1}H NMR (500 MHz, DMSO-d_6) δ 7.32 – 7.26 (m, 2H), 7.13 (dd, J = 7.1, 2.5 Hz, 1H), 6.70 (s, 1H), 5.76 (d, J = 7.9 Hz, 1H), 3.40 – 3.32 (m, 1H), 3.15 (t, J = 7.2 Hz, 2H), 2.97 (s, 4H), 2.87 (q, J = 6.5 Hz, 2H), 2.73 (s, 3H), 2.60 – 2.50 (m, 4H), 2.36 (s, 2H), 1.74 (t, J = 13.0 Hz, 4H), 1.51 (p, J = 7.0 Hz, 2H), 1.40 – 1.33 (m, 11H), 1.25 – 1.14 (m, 3H), 1.00 – 0.90 (m, 2H) ppm.^{13}C NMR (125 MHz, DMSO-d_6) δ 157.60, 155.92, 151.64, 133.05, 128.84, 126.44, 124.73, 119.95, 77.89, 56.11, 53.31, 51.39, 49.89, 45.76, 37.89, 35.31, 34.24, 33.39, 32.41, 28.71, 28.20 ppm.

*Synthesis of tert-butyl 3-(3-aminopropyl)-3-methyl-1-[(1R,4R)-4-{2-[4-(2,3-dichlorophenyl)piperazin-1-yl]ethyl}cyclohexyl]urea (**20**)*

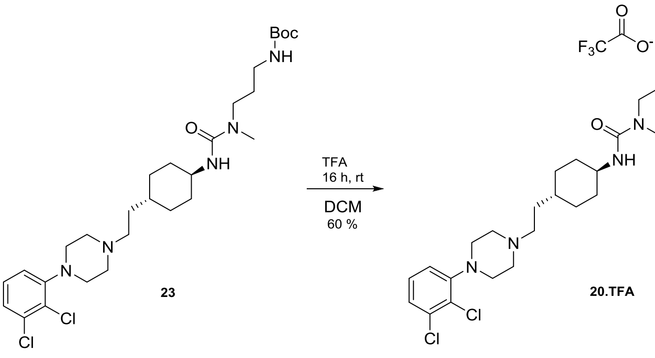

In a round bottom flask, *t*-butyl N-{3-[methyl({[(1R,4R)-4-{2-[4-(2,3-dichlorophenyl)piperazin-1-yl]ethyl}cyclohexyl]carbamoyl})amino]propyl}carbamate (100 mg, 0.17 mmol) was dissolved in 3 ml DCM and 1 ml TFA was added to the solution. The reaction mixture was stirred at room temperature for 16 h. On the next step, the pH was set 10 with aqueous NH_3 solution (25%). The mixture was washed with water and extracted with DCM. The organic phase was dried over Na_2SO_4 and evaporated. The crude product was purified by flash chromatography, eluting with DCM:MeOH (0-10%). 50 mg (60%) white solid was obtained. HRMS (ESI) $(M+H)^+$ calcd. for $C_{23}H_{38}N_5OCl_2$, 470.2447; found 470,2446.

^{1}H NMR (500 MHz, CD_3OD) δ 7.32 – 7.29 (m, 2H), 7.18 (dd, *J* = 5.7, 3.6 Hz, 1H), 3.70 (d, *J* = 10.5 Hz, 2H), 3.52 (d, *J* = 12.0 Hz, 3H), 3.44 – 3.38 (m, 1H), 3.37 – 3.18 (m, 9H), 3.16 – 3.11 (m, 2H), 3.11 – 3.05 (m, 2H), 2.74 (s, 3H), 2.15 – 2.05 (m, 3H), 1.99 – 1.82 (m, 4H), 1.77 (s, 2H), 1.44 (dt, *J* = 25.5, 11.3 Hz, 2H), 1.24 – 1.10 (m, 2H) ppm. ^{13}C NMR (126 MHz, CD_3OD) δ 149.35, 133.69, 127.95, 127.23, 125.65, 119.24, 52.11, 52.08, 42.05, 36.50, 35.78, 34.65, 34.01, 32.53, 32.34, 31.54, 31.40, 30.15, 30.06, 29.96, 23.84, 22.28.

*Synthesis of 1-(6-((3-(3-((1R,4R)-4-(2-(4-(2,3-dichlorophenyl)piperazin-1-yl)ethyl)cyclohexyl)-1-methylureido)propyl)amino)-6-oxohexyl)-3,3-dimethyl-2-((1E,3E)-5-((Z)-1,3,3-trimethyl-5-sulfoindolin-2-ylidene)penta-1,3-dien-1-yl)-3H-indol-1-ium-5-sulfonate (**18**)*

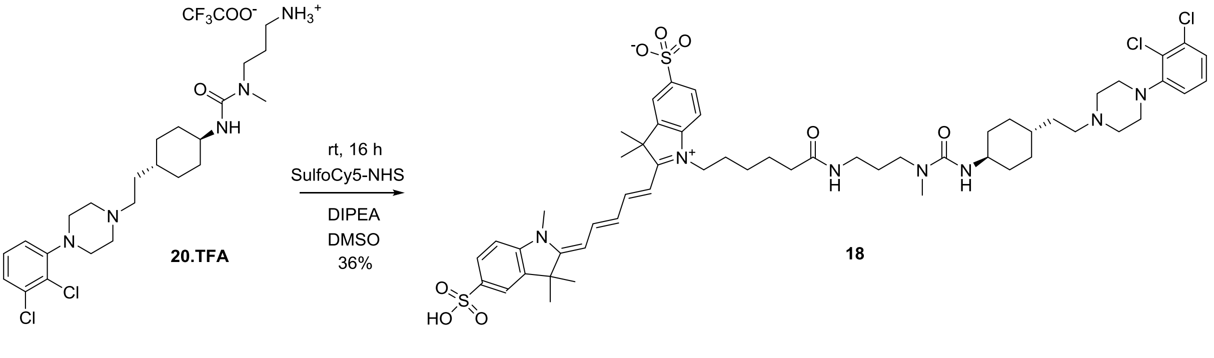

In a round bottom flask, 1-(3-aminopropyl)-3-(4-(2-(4-(2,3-dichlorophenyl)piperazin-1-yl)ethyl)cyclohexyl)-1-methylurea (10 mg, 21.3 μmol) was dissolved in 1 mL DMSO, followed by the addition of ethyl-diisopropylamine (5.0 μL, 63.9 μmol, 3 equiv.) and Sulfo-Cy5 NHS ester (12 mg, 15.4

μmol, 0.72 equiv.). The reaction mixture was stirred in dark at room temperature overnight, and was purified by preparative HPLC resulting the product as a blue solid (6 mg, 36%).

^{1}H NMR (500 MHz, CD_3OD) δ 8.37-8.23 (m, 1H), 7.93-7.84 (m, 2H), 7.38-7.31 (m, 1H), 7.31-7.27 (m, 1H), 7.23-7.15 (m, 1H), 6.67 (td, J = 12.4, 5.1 Hz, 1H), 6.38-6.28 (m, 1H), 4.51 (s, 1H), 4.19-4.10 (m, 1H), 3.68-3.62 (m, 1H), 3.46-3.32 (m, 2H), 3.24-3.16 (m, 1H), 3.12 (t, J = 6.4 Hz, 1H), 3.08-3.01 (m, 1H), 2.82 (s, 1H), 2.65 (s, 1H), 2.20 (t, J = 7.1 Hz, 1H), 2.15-2.09 (m, 1H), 1.90-1.79 (m, 1H), 1.77-1.69 (m, 7H), 1.69-1.40 (m, 4H), 1.39-1.16 (m, 3H), 1.00-0.86 (m, 1H), 0.85-0.73 (m, 1H). ^{13}C NMR (125 MHz, CD_3OD) δ 175.1, 173.3, 158.6, 149.7, 144.1, 142.3, 141.6, 141.2, 141.1, 141.0, 133.7, 127.9, 126.6, 125.5, 119.8, 119.2, 119.1, 110.2, 109.3, 104.2, 103.8, 55.4, 54.8, 52.3, 52.3, 49.2, 49.0, 48.8, 48.4, 48.2, 48.0, 47.9, 36.2, 35.4, 35.1, 34.7, 33.5, 33.0, 32.6, 31.6, 31.6, 31.1, 30.7, 30.4, 27.1, 26.7, 26.5, 26.3, 26.3, 25.8, 25.6, 25.4, 25.2, 25.0 ppm. HRMS (ESI) $(M+2H)^{2+}$ calcd. for $C_{55}H_{75}N_7O_8Cl_2S_2$, 547.7242; found 547.7225.

Experimental procedures for the synthesis of the fluorescent activity-based probe DH-463 (34)

Reagents and conditions of Supplementary Fig. S3: i) NaN3, H2O, 80 oC, 96%; ii) MsCl, TEA, DCM; iii) 4-hydroxybenzaldehyde, K2CO3, DMF, 80 oC, 86%; iv) 4-chlorophenyl magnesium bromide, THF, -78 oC, 89%; v) SOCl2, DCM, 40 oC; vi) tert-butyl piperazine-1-carboxylate, K2CO3, DCM, 40 oC, 89%; vii) HCl in 1,4-dioxane, ethyl acetate; viii) HFIP, triphosgene, DIPEA, DCM, 87%. ix) CuSO4, sodium ascorbate, DCM/H2O, r.t., 24 h, 38%.

*Synthesis of (4-(2-Azidoethoxy)phenyl)(4-chlorophenyl)methanol (**28**).*

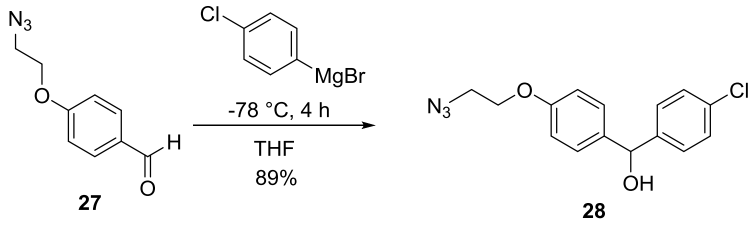

To a stirring solution of **27** (1.2 g, 6.28 mmol) in dry THF (25 mL) at −78 °C under argon atmosphere was added (4-chlorophenyl)magnesium bromide (12.55 mL, 12.55 mmol) (1.0 M in diethyl ether). After 4 h, the reaction was quenched with saturated aqueous $NaHCO_3$ (5 mL) and the aqueous layer extracted with DCM (3x80 mL). The combined organic layers were dried over $MgSO_4$ and concentrated under reduced pressure. The residue was purified by flash chromatography (5−15% EtOAc/pentane) to give product **5** (1.7 g, 5.60 mmol, 89% yield). LC/MS calculated for $[C_{15}H_{14}ClN_3O_2]^+$: 303.75, found: 304.12; ^{1}H NMR (400 MHz, $CDCl_3$) δ 7.30 (s, 4H), 7.28 – 7.22 (m, 2H), 6.89 (d, J = 8.7 Hz, 2H), 5.77 (s, 1H), 4.13 (t, J = 5.0 Hz, 2H), 3.58 (t, J = 4.9 Hz, 2H), 2.22 (br, 1H). ^{13}C NMR (101 MHz, $CDCl_3$) δ 157.64, 142.40, 136.35, 132.88, 128.39, 127.87, 127.71, 114.50, 77.16, 74.79, 66.90, 49.98.

*Synthesis of tert-Butyl 4-((4-(2-azidoethoxy)phenyl)(4-chlorophenyl)methyl)piperazine-1-carboxylate (**30**).*

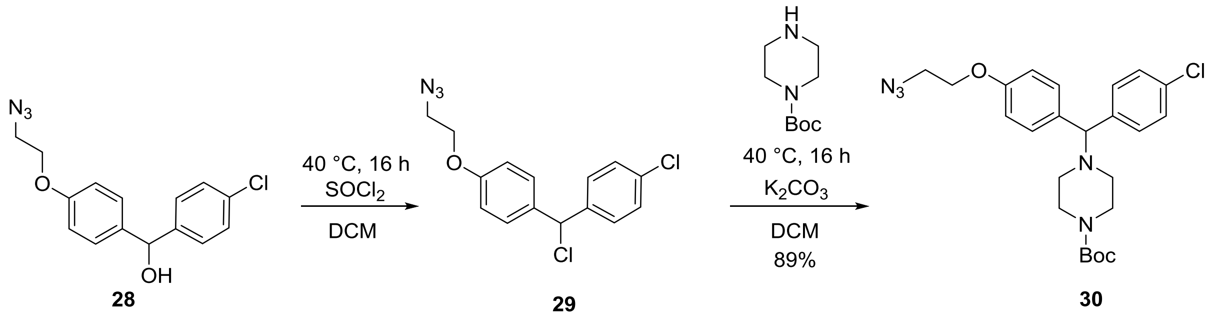

To a stirring solution of **28** (800 mg, 2.63 mmol) in dry DCM (5 mL) at room temperature under argon atmosphere was added thionyl chloride (2.1 mL, 29 mmol). After stirred at 40 °C for overnight, the reaction was quenched with saturated aqueous $NaHCO_3$ (20 mL) and the aqueous layer extracted with DCM (3 × 10 mL). The combined organic layers were dried over $MgSO_4$, filtered and concentrated under reduced pressure to give crude product **29**, which was used for next step without further purification. To a solution of **29** (850 mg, 2.64 mmol) in DCM (12 mL) was added *tert*-butyl piperazine-1-carboxylate (1.5 g, 7.9 mmol) and K_2CO_3 (1.9 g, 13.7 mmol) at room temperature. After stirred at 40 °C for overnight, the reaction was quenched with saturated aqueous $NaHCO_3$ (20 mL) and the aqueous layer extracted with CH_2Cl_2 (3x10 mL). The combined organic layers were dried over Na_2SO_4 and concentrated under reduced pressure. The mixture was purified by flash chromatography (5−15% EtOAc/pentane) to give title compound **7** (1.2 g, 2.4 mmol, 89% yield). LC/MS calculated for $[C_{24}H_{30}ClN_5O_3]^+$: 471.99, found: 472.52; ^{1}H NMR (400 MHz, $CDCl_3$) δ 7.33 (d, *J* = 8.5 Hz, 2H), 7.29 – 7.21 (m, 4H), 6.84 (d, *J* = 8.7 Hz, 2H), 4.17 (s, 1H), 4.10 (t, *J* = 4.0 Hz, 2H) 3.56 (t, *J* = 5.0 Hz, 2H), 3.44 – 3.36 (m, 4H), 2.31 (br, 4H), 1.43 (s, 9H); ^{13}C NMR (101 MHz, $CDCl_3$) δ 157.51, 154.90, 141.39, 134.77, 132.71, 129.13, 129.09, 128.86, 114.81, 79.68, 74.69, 67.08, 51.72, 50.23, 44.38, 28.54.

*Synthesis of 1-((4-(2-Azidoethoxy)phenyl)(4-chlorophenyl)methyl)piperazine (**31**).*

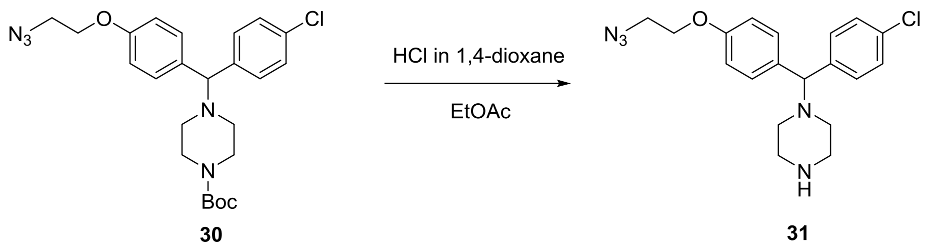

To a solution of compound **30** (300 mg, 0.64 mmol) in ethyl acetate (2 mL) was added HCl (0.8 mL, 3.2 mmol) in 1,4-dioxane. After TLC analysis indicated complete consumption of starting material, the mixture was poured into a saturated solution of $NaHCO_3$ and the product was extracted with DCM (3x20 mL). The combined organic layers were dried over $MgSO_4$, filtered and concentrated under reduced pressure to provide crude product **31**, which was used in the next step without further purification.

LC/MS calculated for $[C_{19}H_{22}ClN_5O]^+$: 371.87, found: 372.64; ^{1}H NMR (400 MHz, $CDCl_3$) δ 7.63 – 7.09 (m, 6H), 6.84 (d, *J* = 8.6 Hz, 2H), 4.61 (br, 1H), 4.20 (s, 1H), 4.10 (t, *J* = 5.0 Hz, 2H), 3.55 (t, *J* = 8.0 Hz, 2H), 2.97 (t, *J* = 4.5 Hz, 4H), 2.44 (br, 4H); ^{13}C NMR (101 MHz, $CDCl_3$) δ 173.00, 157.42, 141.20, 134.55, 132.61, 129.04, 128.98, 128.76, 114.74, 74.87, 66.98, 51.85, 50.12, 45.53.

*Synthesis of 1,1,1,3,3,3-Hexafluoropropan-2-yl 4-((4-(2-azidoethoxy)phenyl)(4-chlorophenyl)methyl) piperazine-1-carboxylate (**32**).*

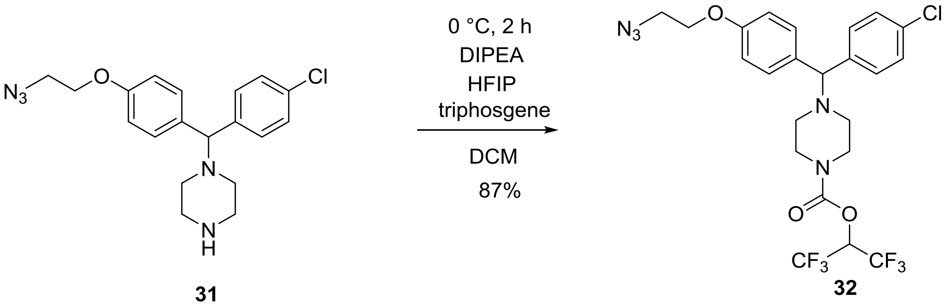

To a stirring solution of triphosgene (80 mg, 0.3 mmol) in CH_2Cl_2 (4 mL) at 0 °C was added the 1,1,1,3,3,3-hexafluoropropan-2-ol (0.062 mL, 0.59 mmol) followed by DIPEA (0.28 mL, 1.6 mmol). After stirring for 2h at room temperature, **31** (200 mg, 0.54 mmol) was added as a solution in CH_2Cl_2 (1 mL) and stirred for another 2 h. The mixture was concentrated under reduced pressure and purified by flash chromatography on silica gel (1−20% EtOAc/pentane) to provide the title compound **9** (165 mg, 0.29 mmol, 54% yield). HRMS calculated for $[C_{23}H_{22}ClF_6N_5O_3]^+$: 566.13881, found: 566.13875; ^{1}H NMR (400 MHz, $CDCl_3$) δ 7.33 (d, *J* = 8.4 Hz, 2H), 7.30 – 7.20 (m, 4H), 6.84 (d, *J* = 8.6 Hz, 2H), 5.74 (septet, *J* = 6.4 Hz, 1H), 4.20 (s, 1H), 4.09 (t, *J* = 4.9 Hz, 2H), 3.59 – 3.48 (m, 6H), 2.38 (dt, *J* = 14.2, 4.1 Hz, 4H); ^{13}C NMR (101 MHz, $CDCl_3$) δ 157.64, 151.44, 140.95, 134.27, 132.91, 129.02, 129.00, 128.95, 114.90, 74.46, 68.87 (q, *J* = 228 Hz), 68.08 (p, *J* = 35.4 Hz), 67.08, 51.36, 51.20, 50.18, 44.92, 44.56.

*Synthesis of 1-(6-(((1-(2-(4-((4-Chlorophenyl)(4-(((1,1,1,3,3,3-hexafluoropropan-2-yl)oxy)carbonyl)piperazin-1-yl)methyl)phenoxy)ethyl)-1H-1,2,3-triazol-4-yl)methyl)amino)-6-oxohexyl)-3,3-dimethyl-2-((1E,3E)-5-((E)-1,3,3-trimethylindolin-2-ylidene)penta-1,3-dien-1-yl)-3H-indol-1-ium chloride (**DH-463**) (**34**).*

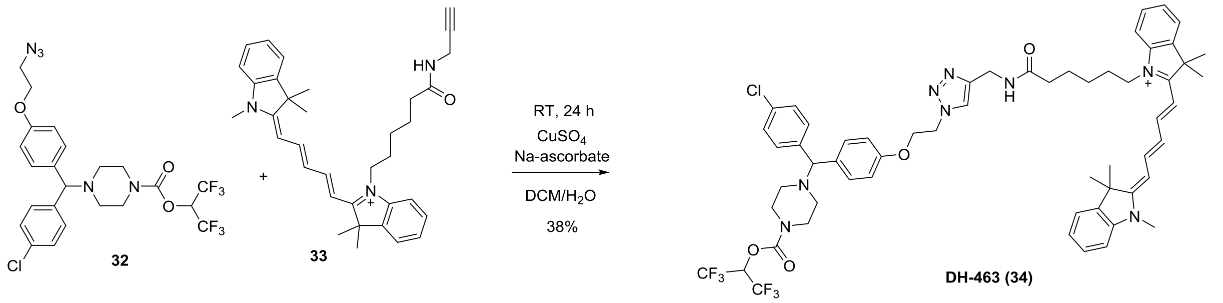

Compound **32** (10 mg, 0.018 mmol) and **33** (9.8 mg, 0.018 mmol) were dissolved in degassed DCM/H_2O (2 mL, 1:1, v/v) and aqueous solutions of sodium ascorbate (4.20 mg, 0.021 mmol) and copper(II) sulfate pentahydrate (2.2 mg, 8.8 μmol) were added. The resulting mixture was stirred vigorously for 2 h, after which TLC indicated completed conversion of the reaction. The solvents were evaporated in vacuo, and the residue was taken up in DCM and purified by silica column chromatography (1−5% DCM/MeOH) to give the desired product **DH-463** (**34**) (7.6 mg, 0.007 mmol, 38% yield). HRMS calculated for $[C_{58}H_{64}ClF_6N_8O_4]^+$ 1085.46378, found: 1085.46335; ^{1}H NMR (600 MHz, $CDCl_3$) δ 7.86 (d, *J* = 10.1 Hz, 2H), 7.42 – 7.32 (m, 4H), 7.26 (s, 8H), 7.10 – 7.08 (m, 2H), 6.99 – 6.72 (m, 4H), 6.38 (br d, *J* = 45.0 Hz, 2H), 5.76 – 5.65 (m, 1H), 4.76 (br, 4H), 4.34 (br, 3H), 4.04 (s, 3H), 3.78 – 3.44 (m, 6H), 2.53 – 2.23 (m, 4H), 1.83 – 1.54 (m, 18H), 1.25 (s, 2H). ^{13}C NMR (151 MHz, $CDCl_3$) δ 173.02, 158.94, 153.36, 152.92, 151.35, 142.86, 142.03, 141.16, 140.88, 128.96, 128.85, 125.48, 125.24, 122.32, 122.23, 110.96, 110.48, 70.29 (q, *J* = 285.4 Hz), 68.13 (p, *J* = 36.2 Hz), 67.92, 66.38, 64.35, 51.47, 51.34, 49.44, 49.20, 45.11, 29.85, 28.30, 28.25, 27.35.

(G) NMR spectra of synthesized compounds

3,5-bis(benzyloxy)-4-bromobenzoate ***(5)***

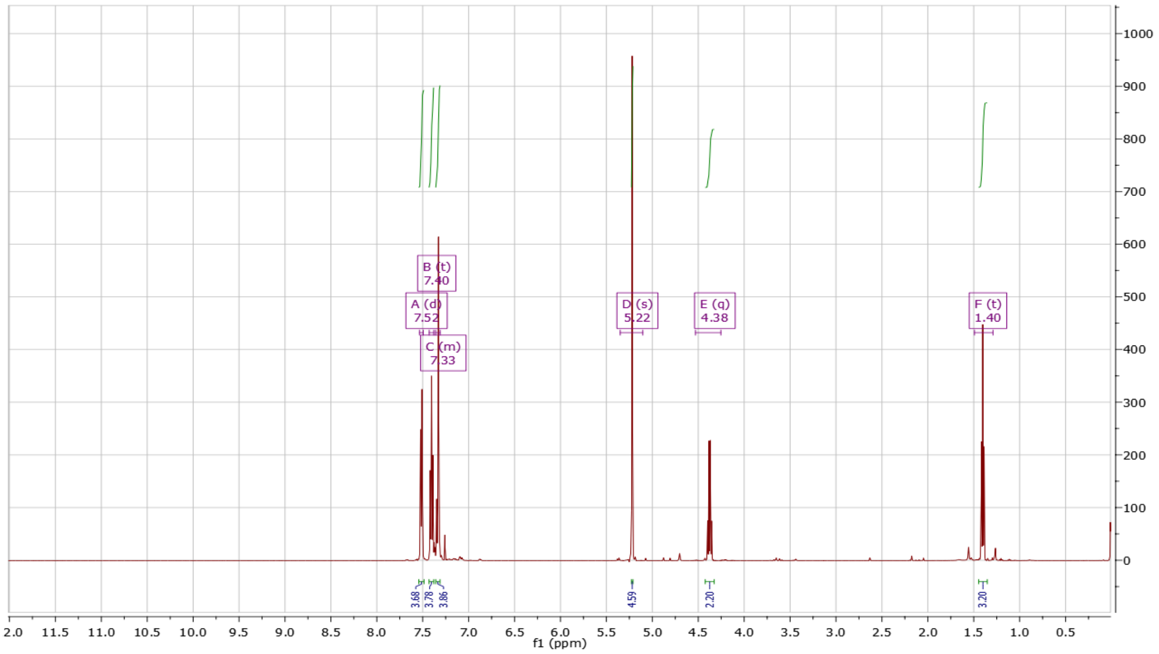

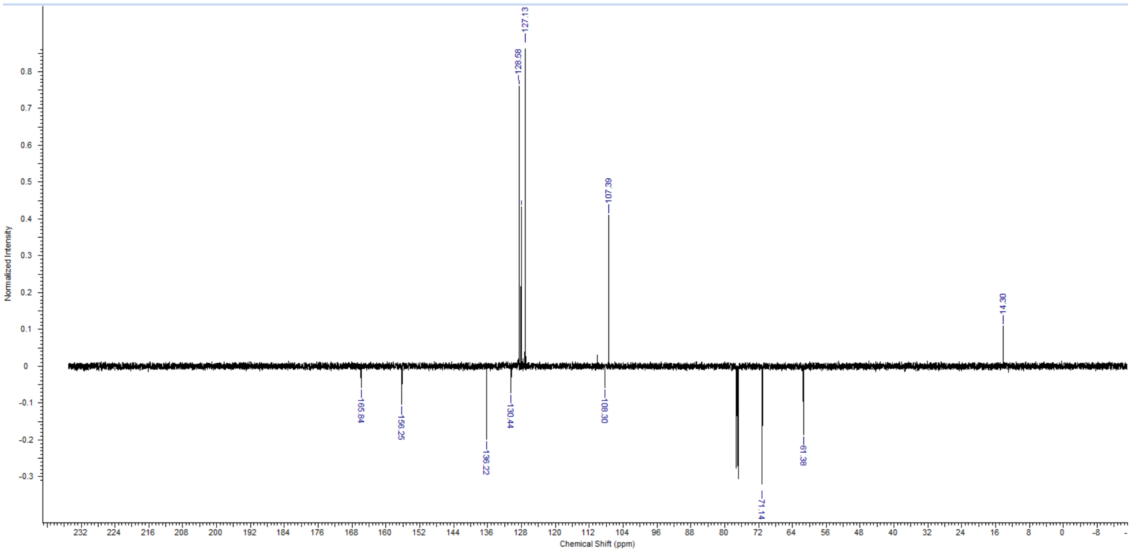

(((2-bromo-5-(chloromethyl)-1,3-phenylene)bis(oxy))bis(methylene))dibenzene ***(7)***

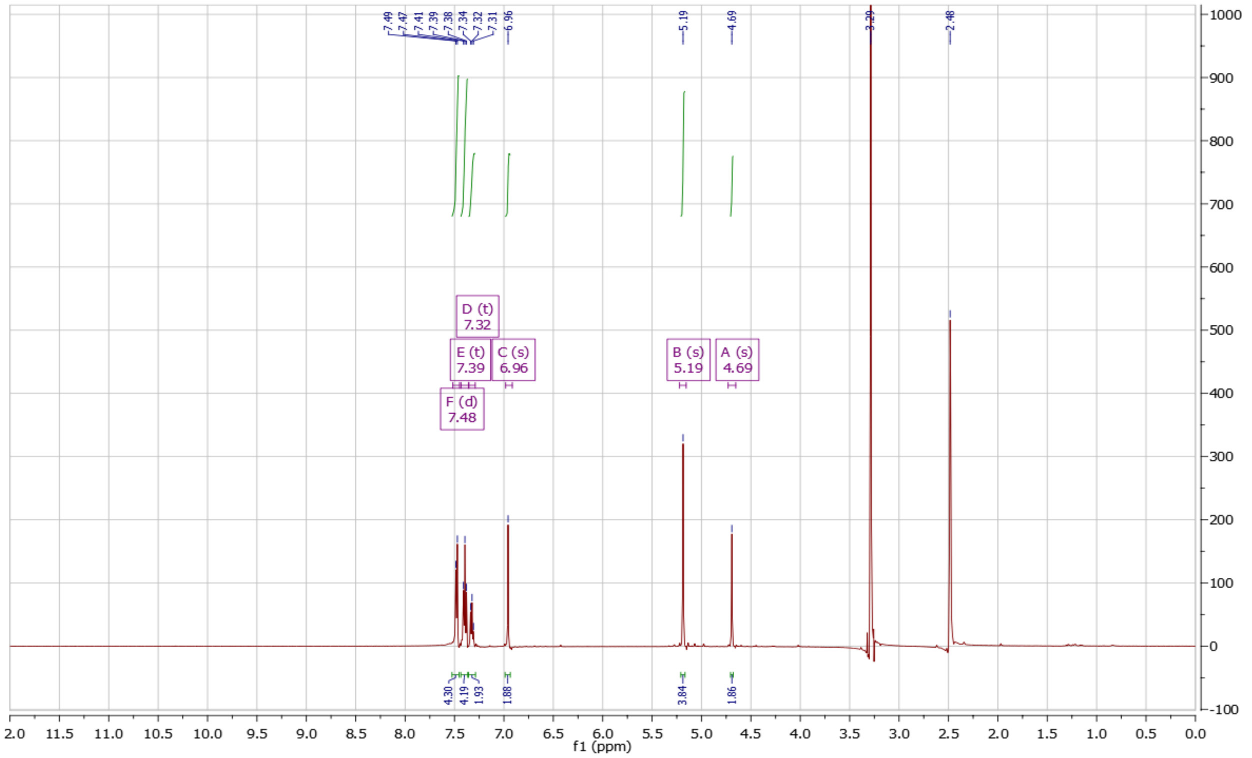

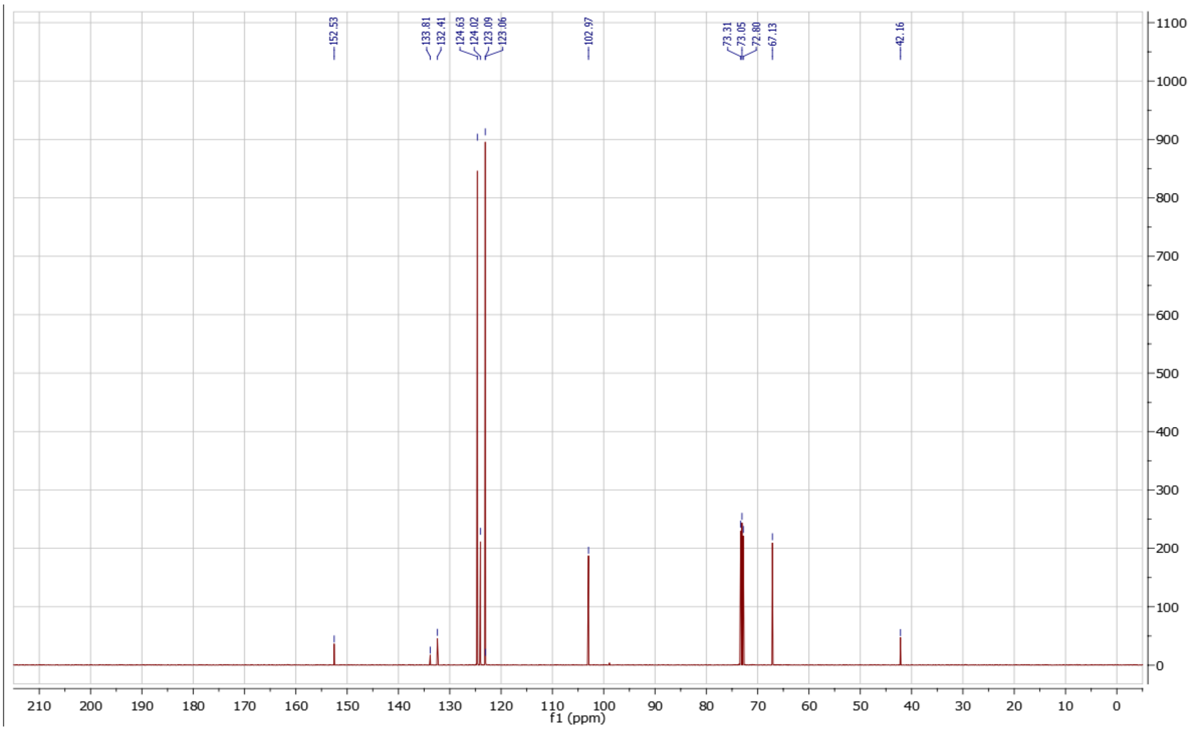

2-(3,5-bis(benzyloxy)-4-bromophenyl)acetonitrile ***(8)***

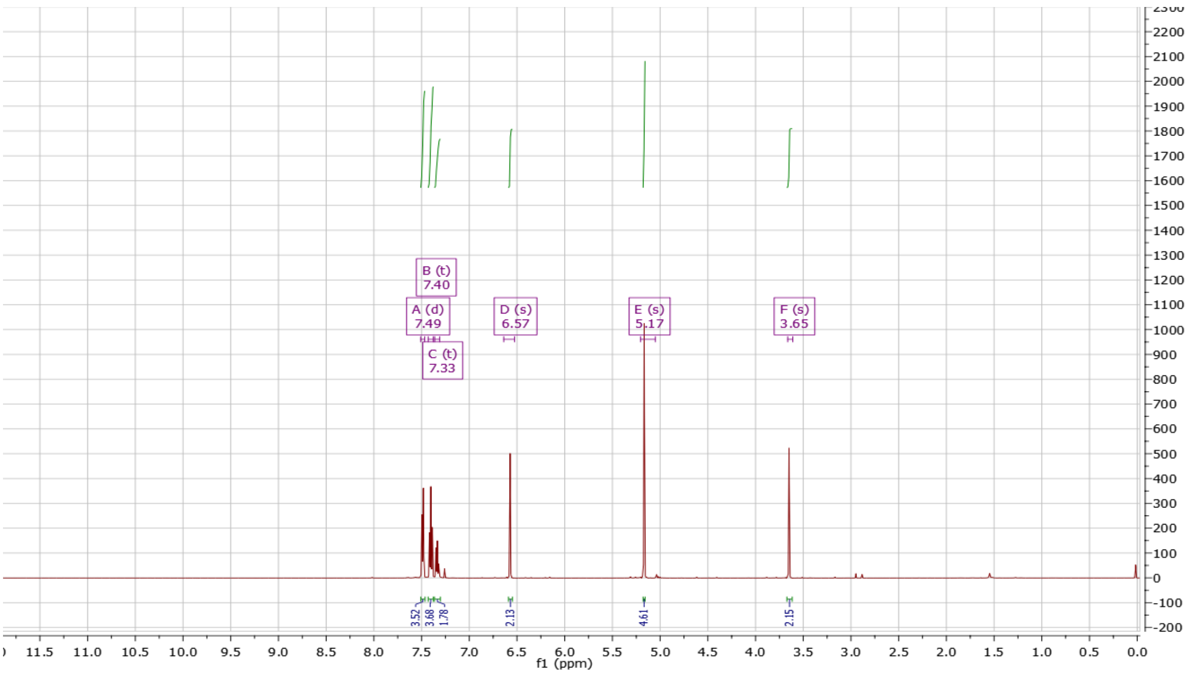

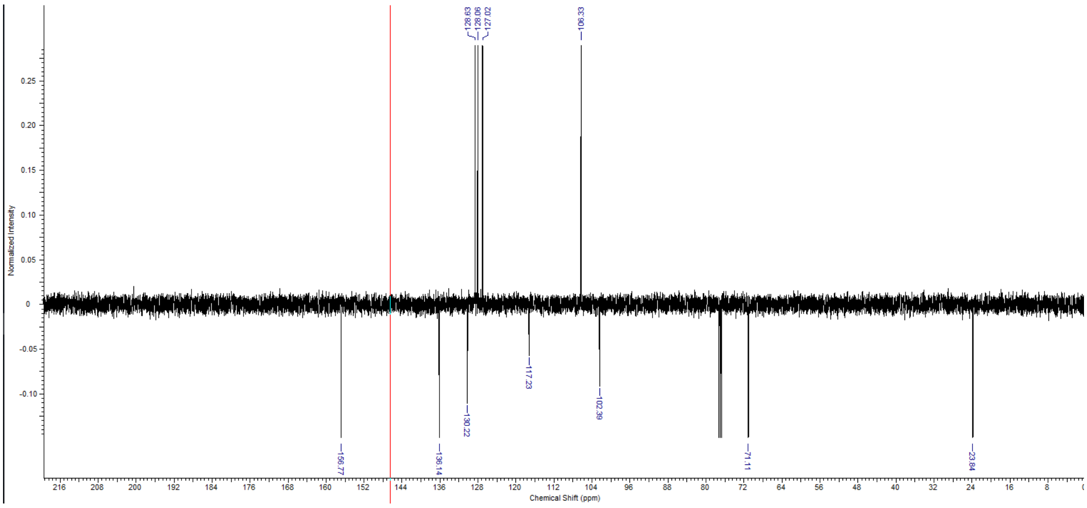

2-(3,5-bis(benzyloxy)-4-bromophenyl)-2-methylpropanenitrile ***(9)***

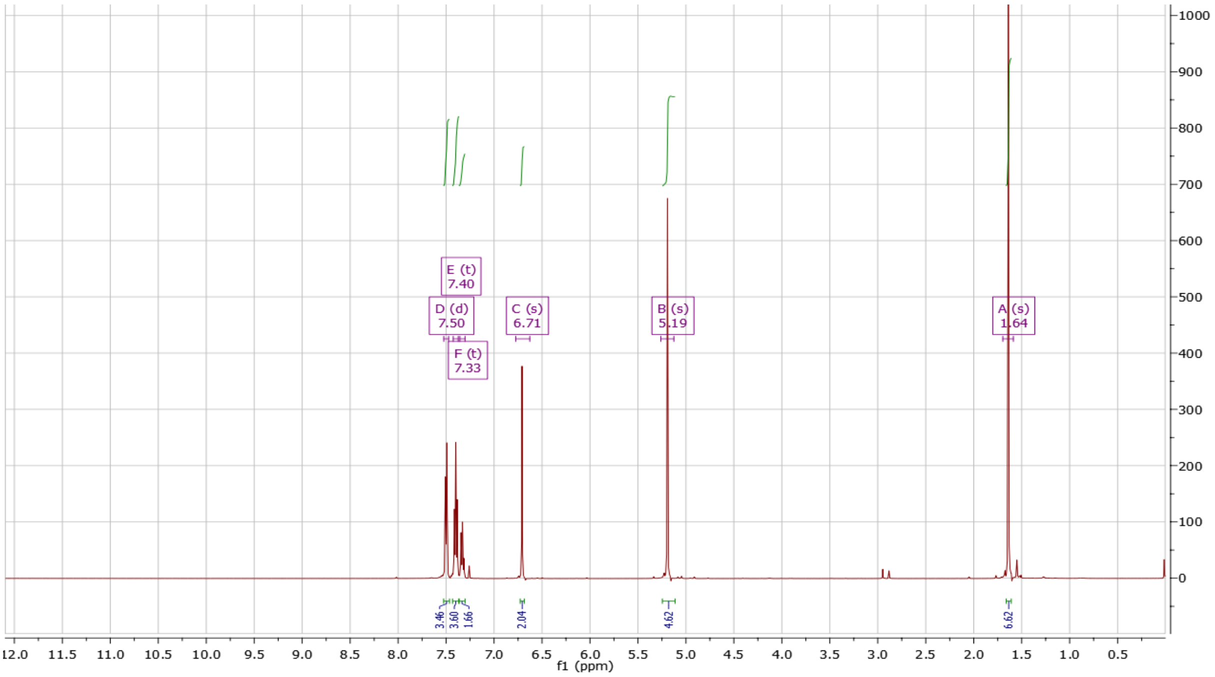

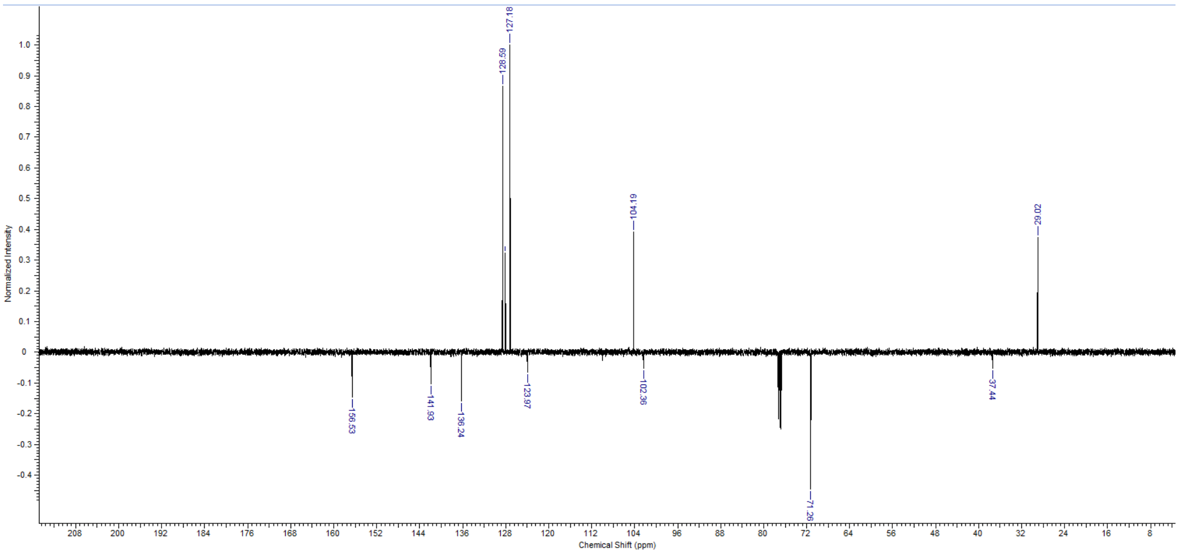

4-(((t-butyldiphenylsilyl)oxy)methyl)-N,N-diisopropylbenzamide ***(11)***

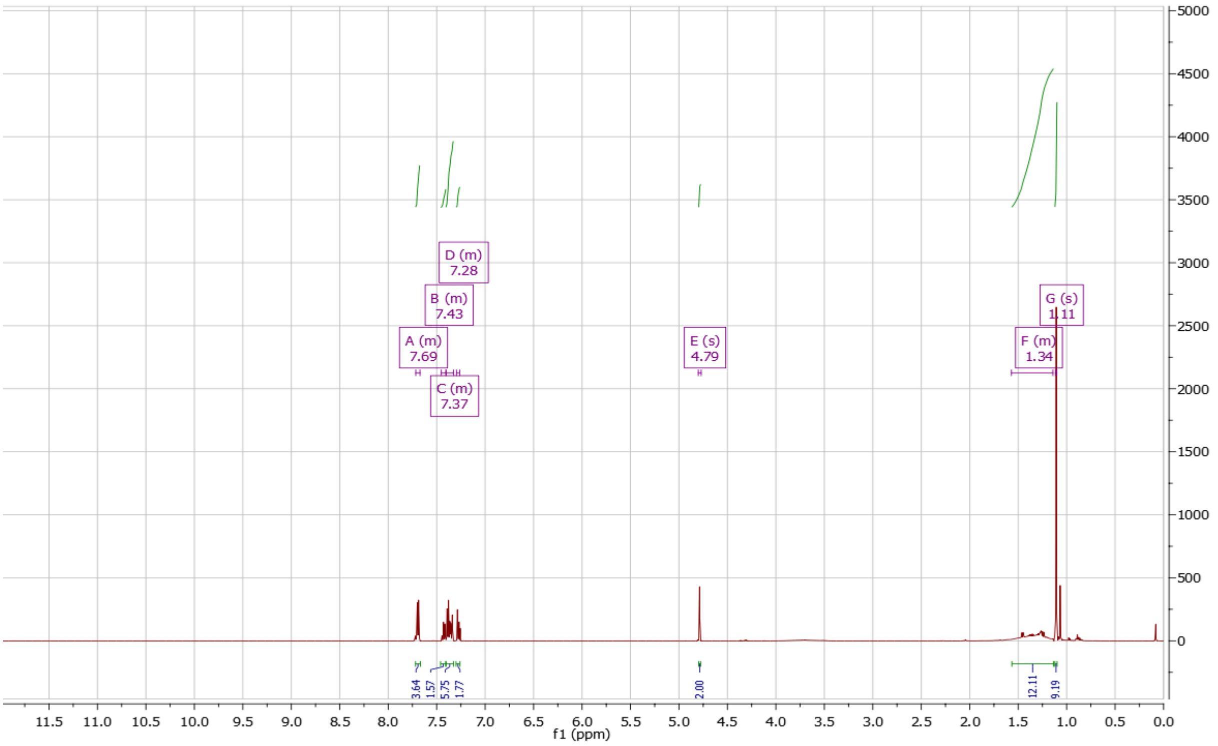

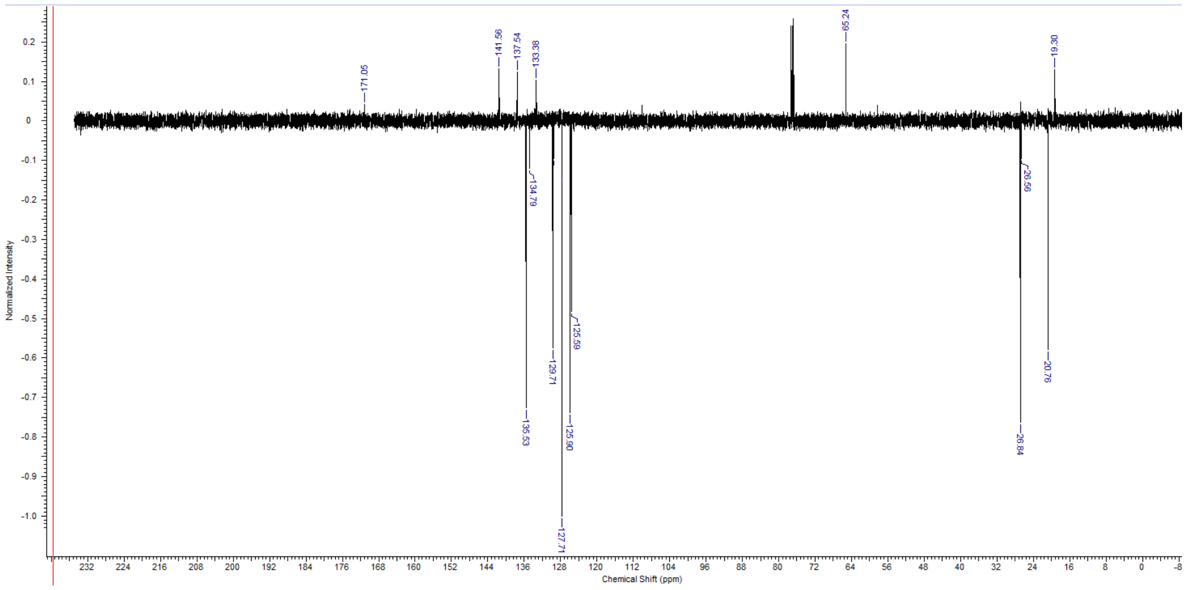

(5-(((t-butyldiphenylsilyl)oxy)methyl)-2-(diisopropylcarbamoyl)phenyl)boronic acid ***(12)***

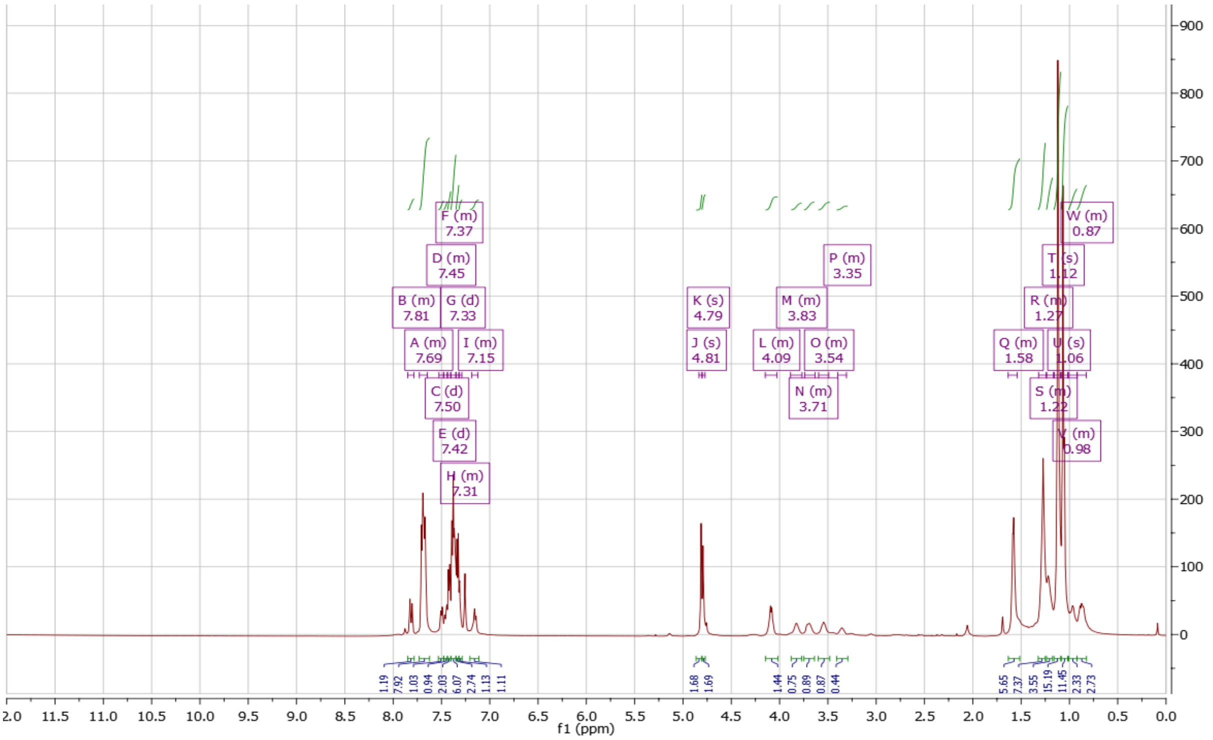

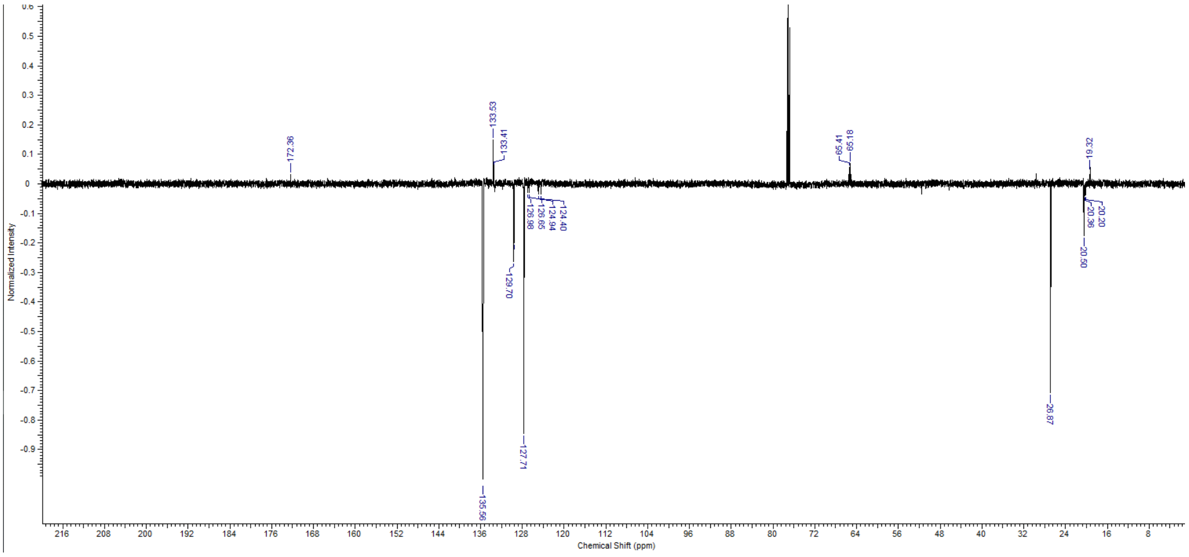

*2',6'-bis(benzyloxy)-5-(((t-butyldiphenylsilyl)oxy)methyl)-4'-(2-cyanopropan-2-yl)-N,N-diisopropyl-[1,1'-biphenyl]-2-carboxamide (**13**)*

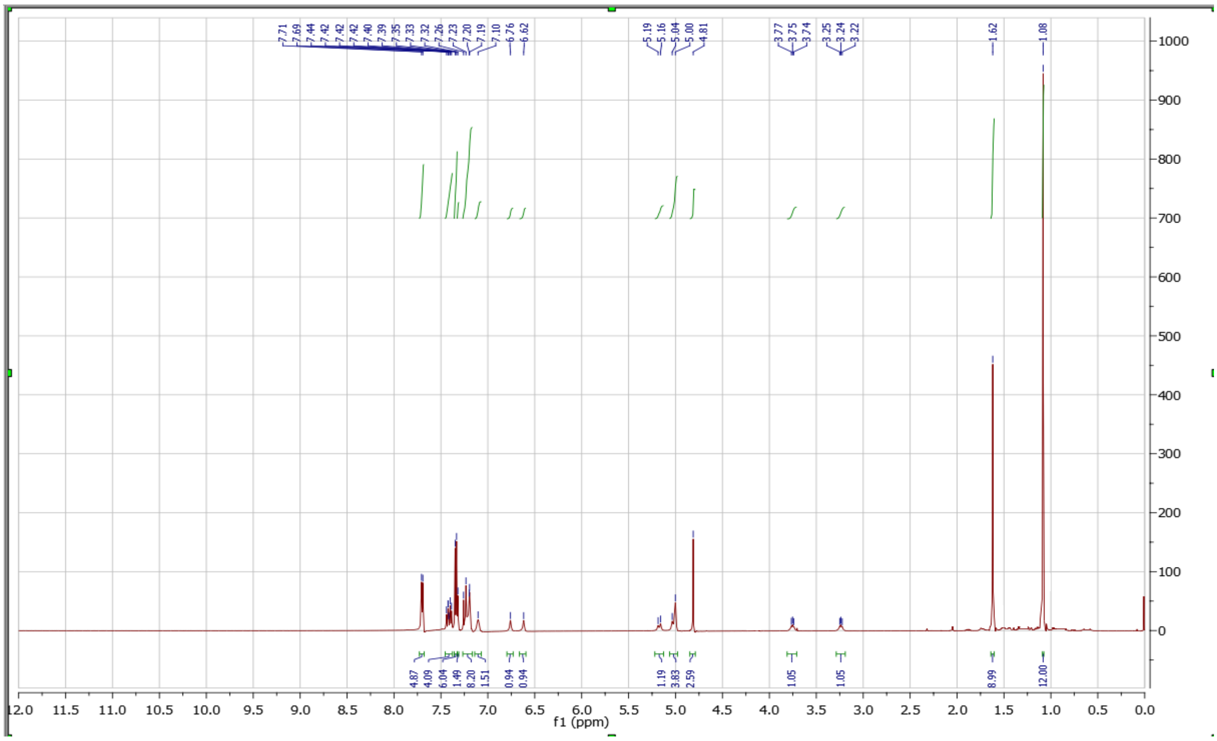

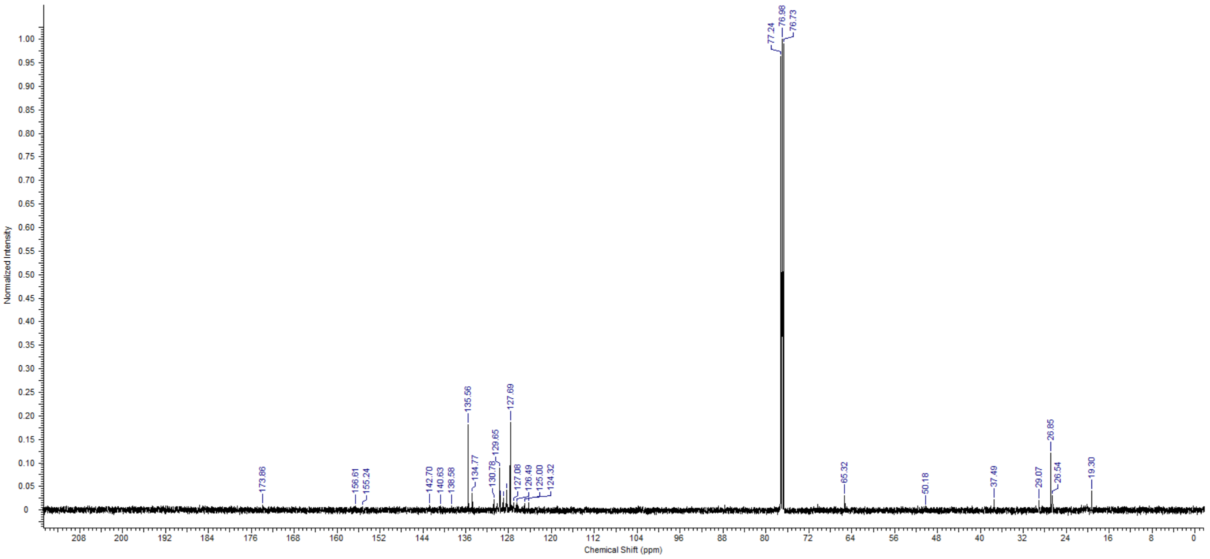

*(E)-2',6'-bis(benzyloxy)-5-(hydroxymethyl)-N,N-diisopropyl-4'-(2-methyloct-3-en-2-yl)-[1,1'-biphenyl]-2-carboxamide (**15**)*

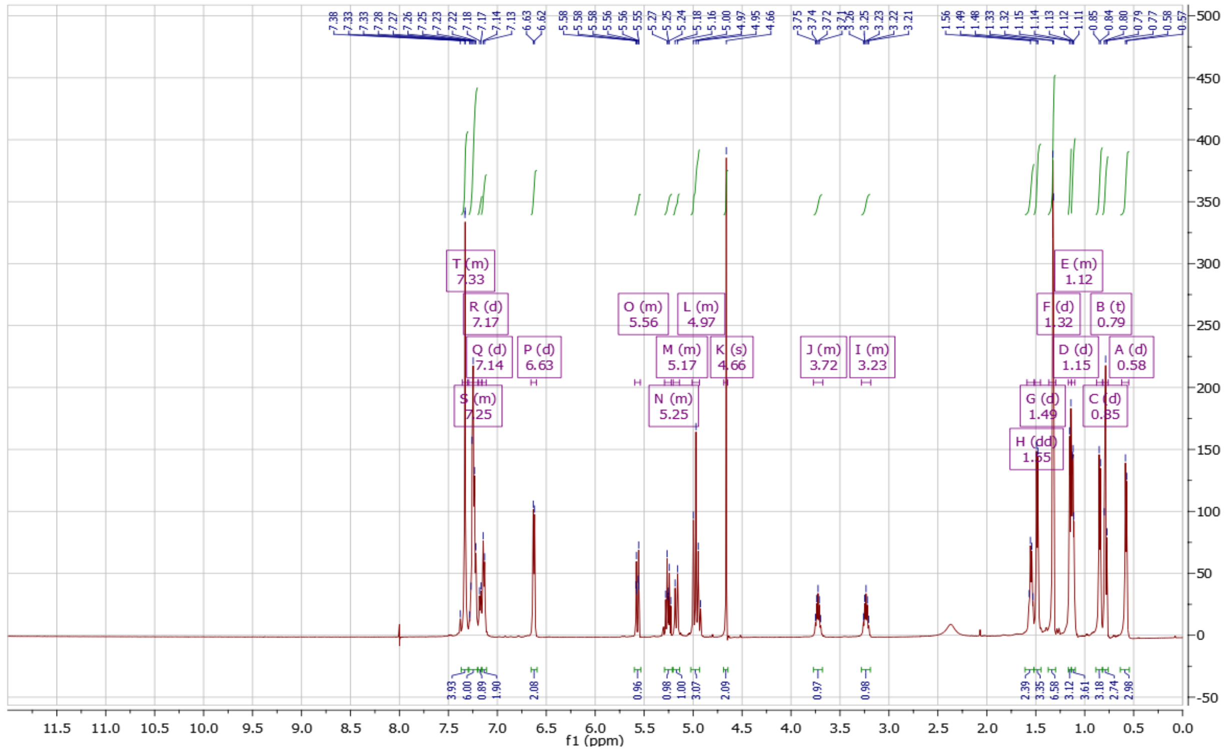

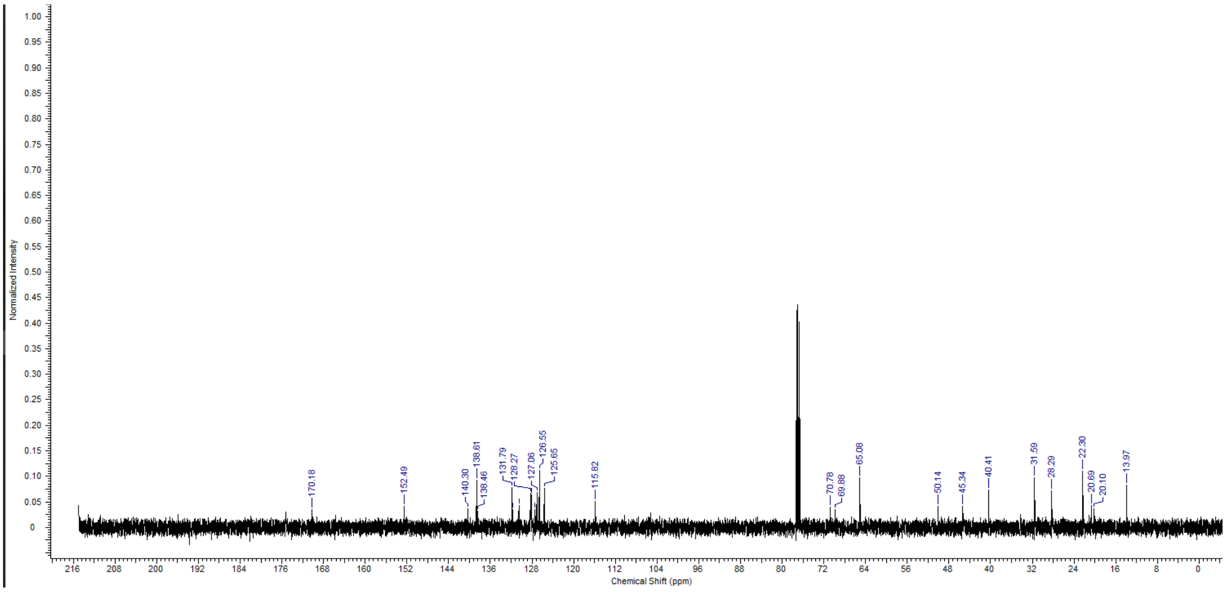

*2',6'-dihydroxy-5-(hydroxymethyl)-N,N-diisopropyl-4'-(2-methyloctan-2-yl)-[1,1'-biphenyl]-2-carboxamide (**16**)*

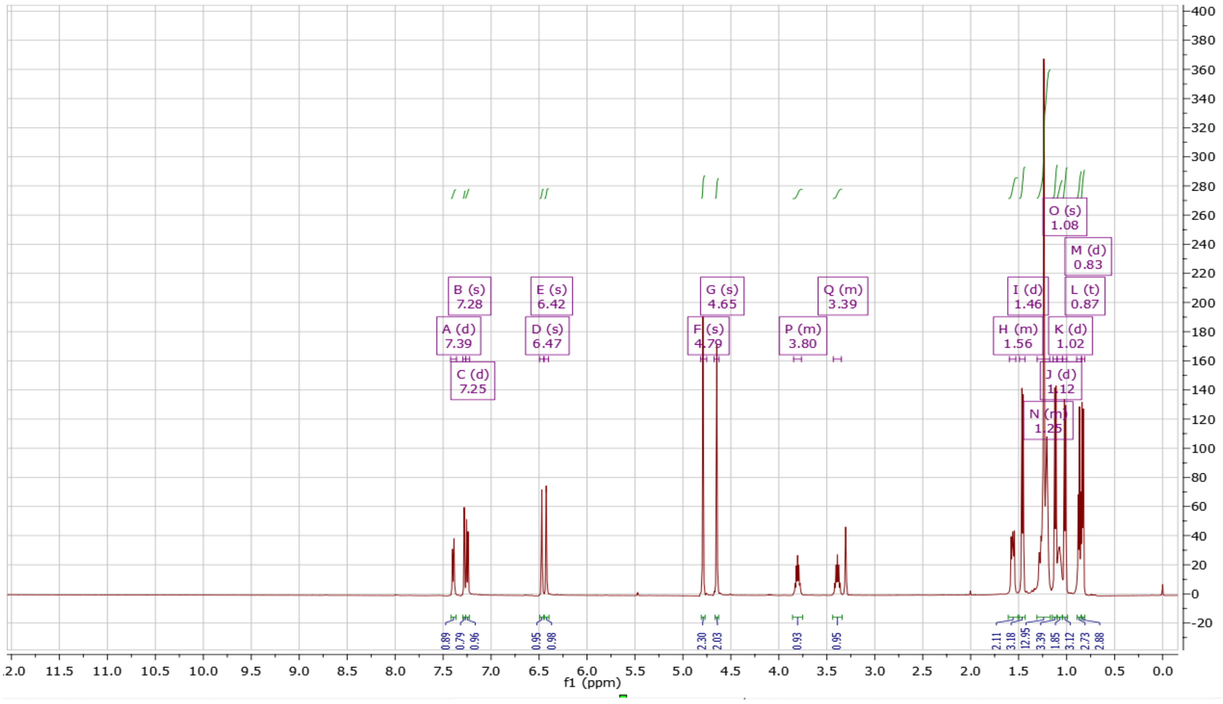

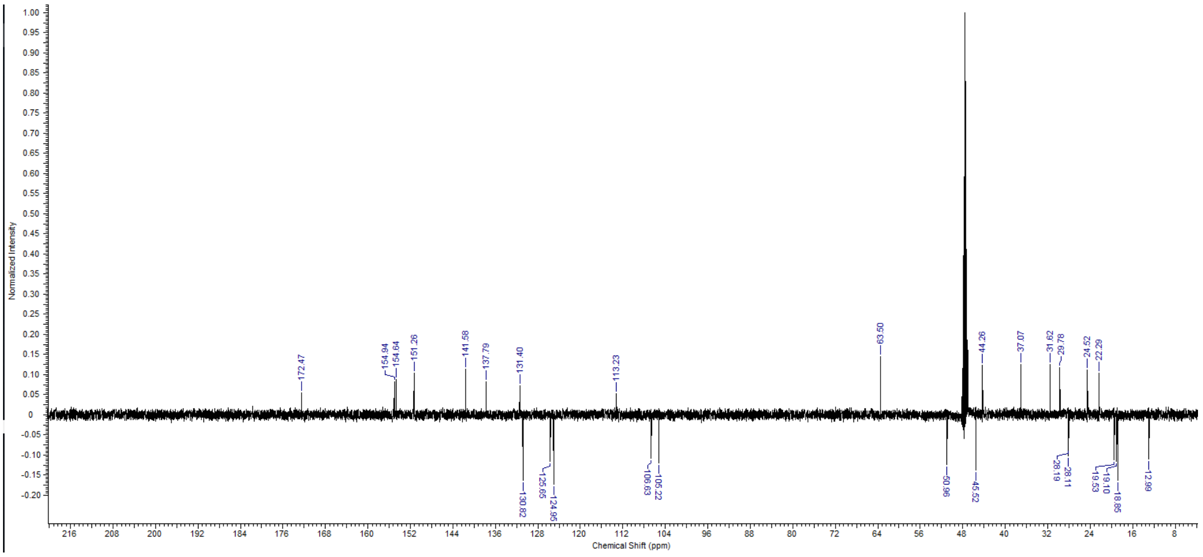

1-hydroxy-9-(hydroxymethyl)-3-(2-methyloctan-2-yl)-6H-benzo[c]chromen-6-one (***17***)

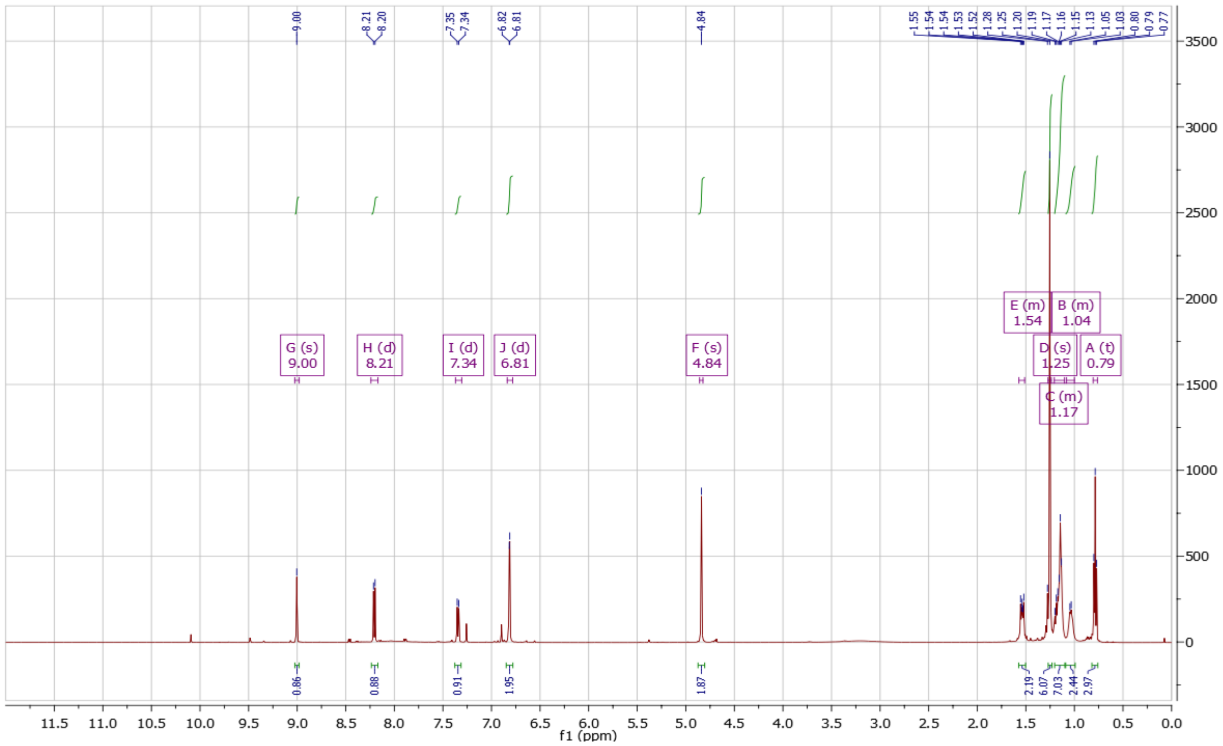

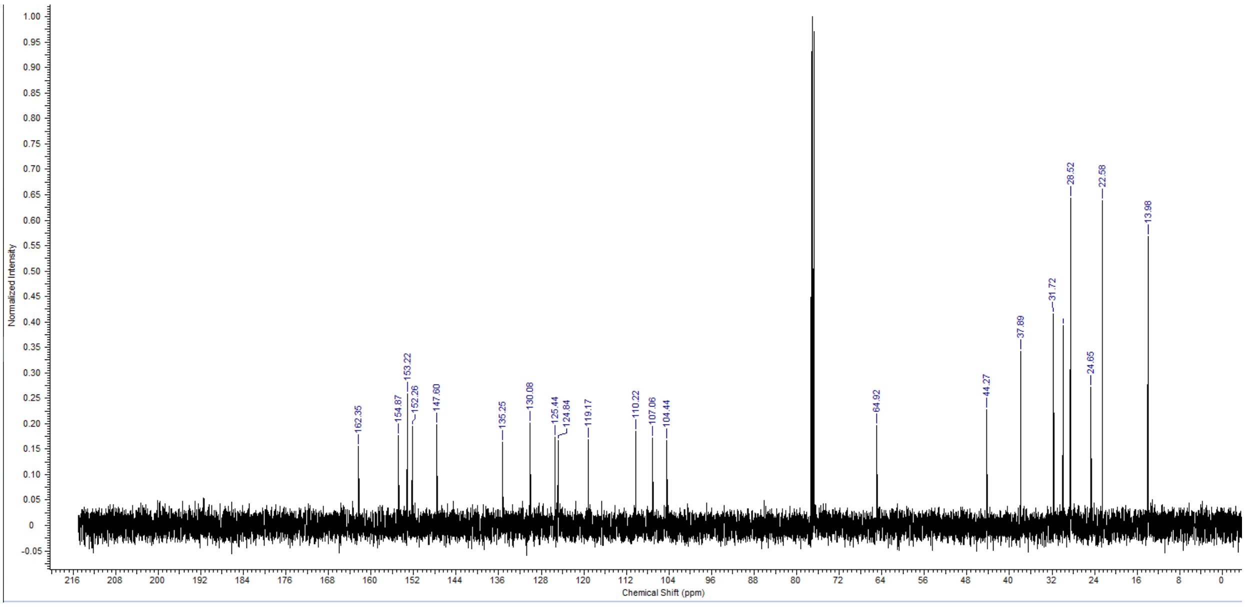

*1-(7-((6-(((1-hydroxy-6,6-dimethyl-3-(2-methyloctan-2-yl)-6H-benzo[c]chromen-9-yl)methyl)amino)hexyl)amino)-7-oxoheptyl)-3,3-dimethyl-2-((1E,3E)-5-((E)-1,3,3-trimethyl-5-sulfoindolin-2-ylidene)penta-1,3-dien-1-yl)-3H-indol-1-ium-5-sulfonate (**1**)*

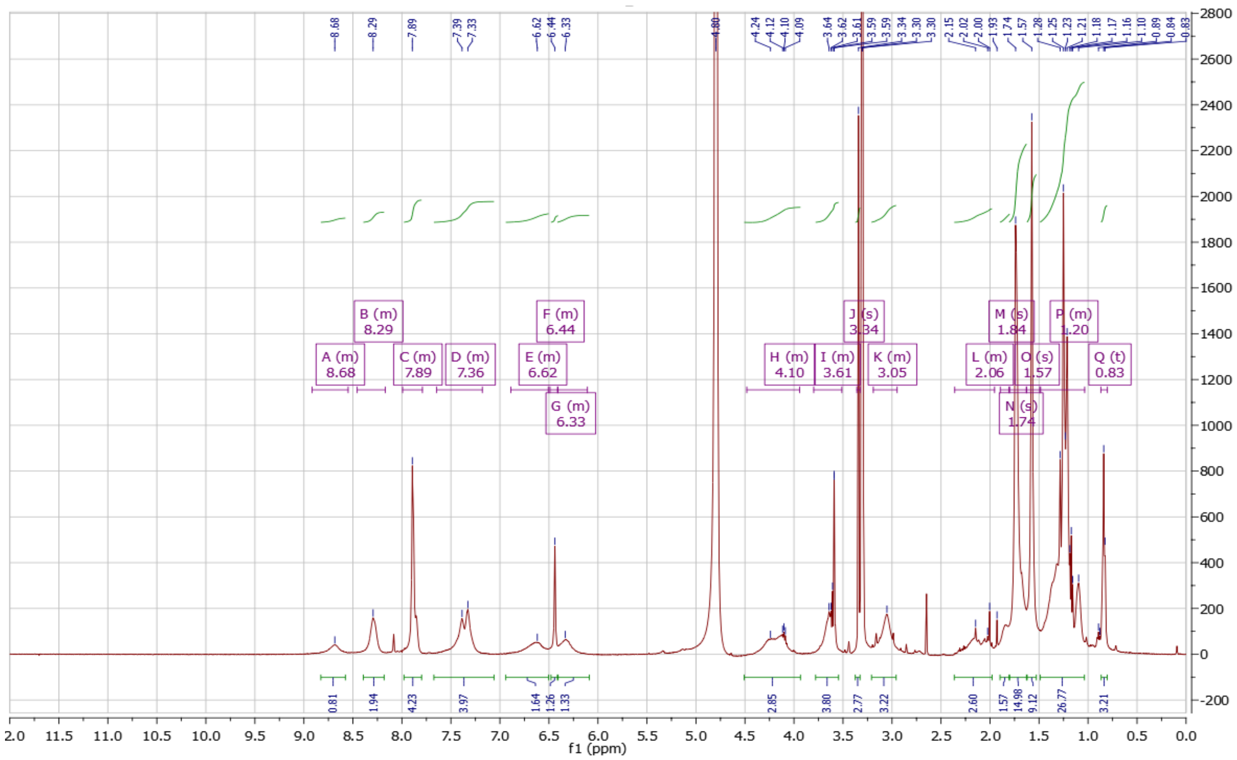

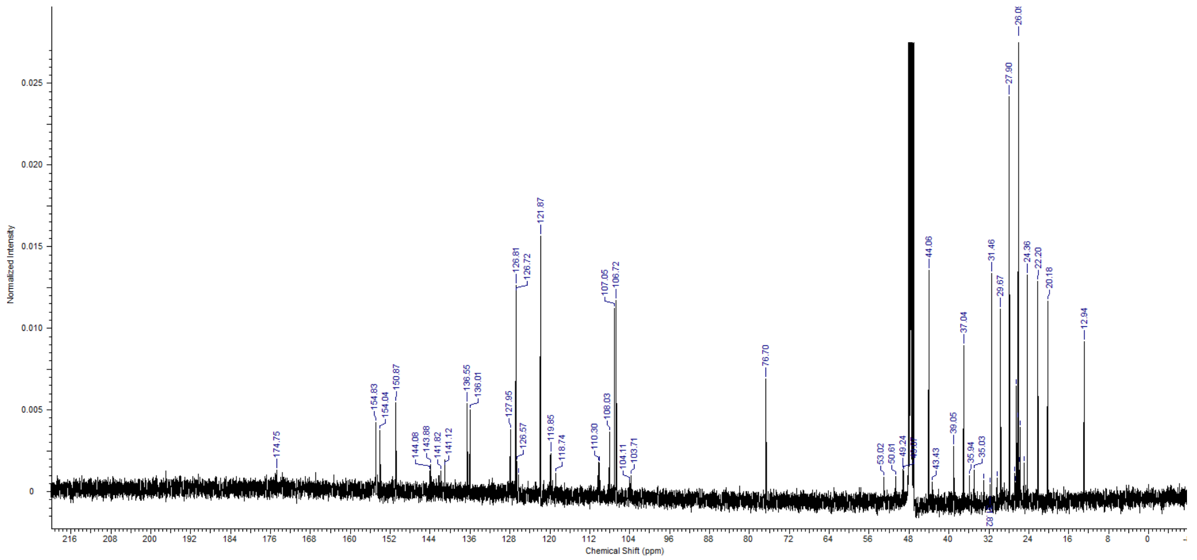

*tert-butyl N-{3-[methyl({[(1R,4R)-4-{2-[4-(2,3-dichlorophenyl)piperazin-1-yl]ethyl}cyclohexyl]carbamoyl})amino]propyl}carbamate (**23**)*

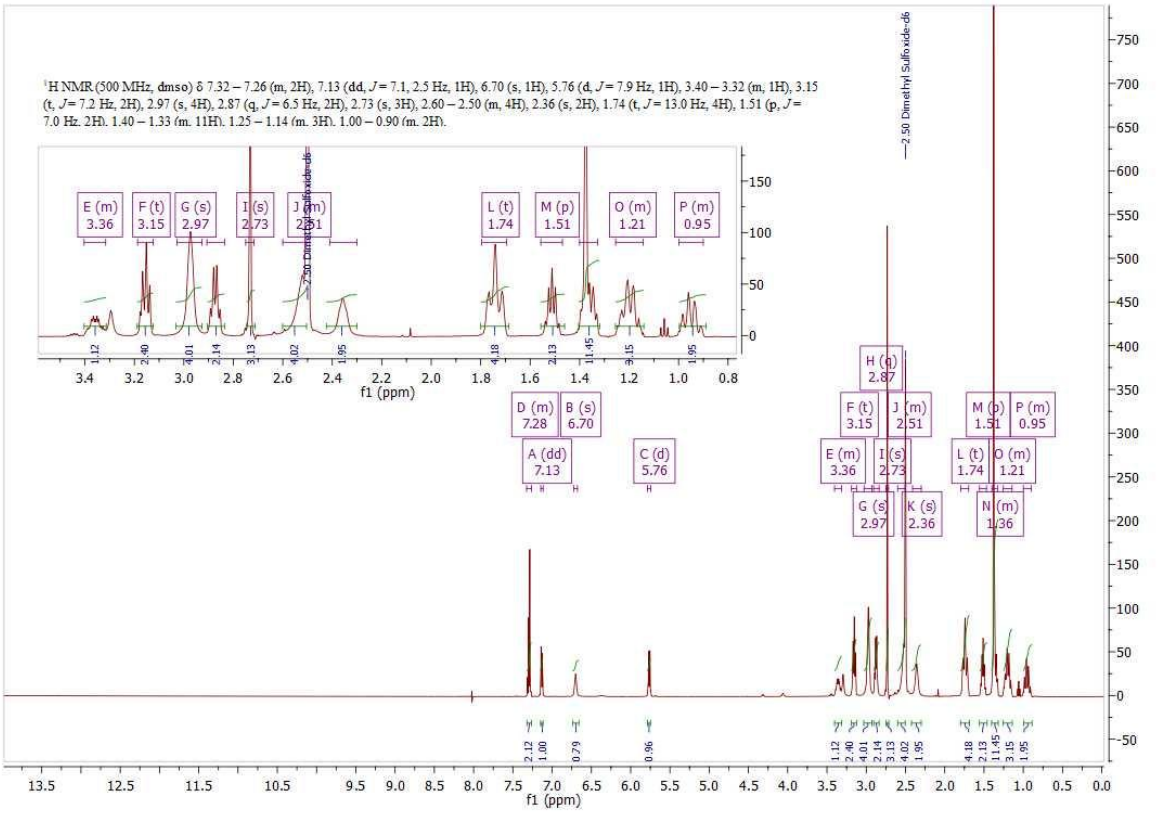

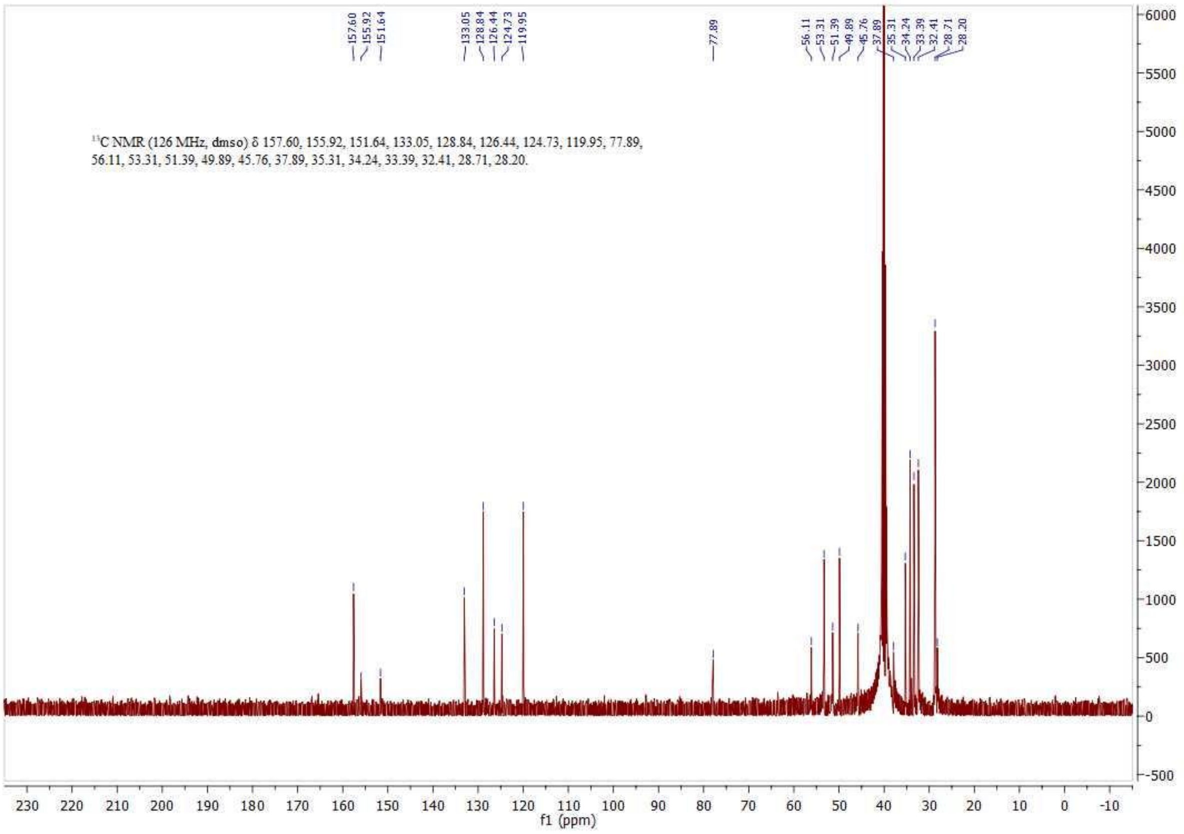

tert-butyl 3-(3-aminopropyl)-3-methyl-1-[(1R,4R)-4-{2-[4-(2,3-dichlorophenyl)piperazin-1-yl]ethyl}cyclohexyl]urea ***(20)***

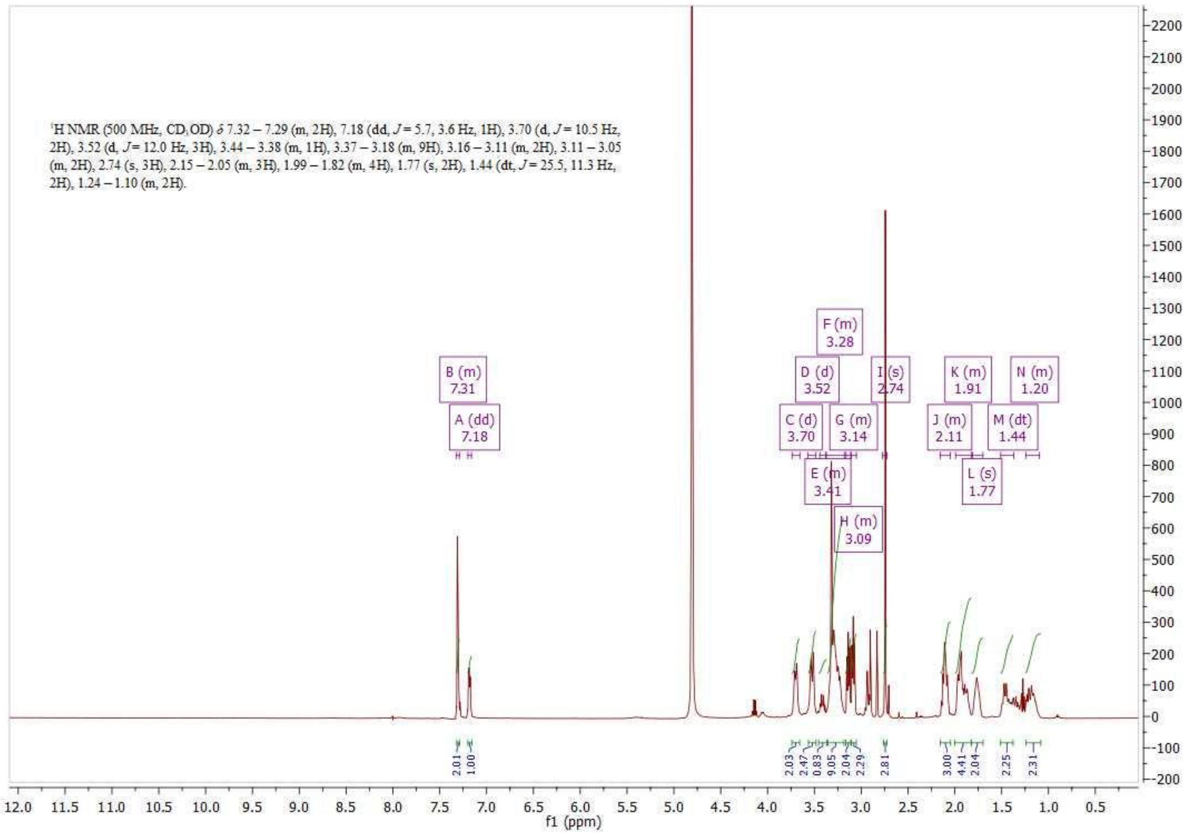

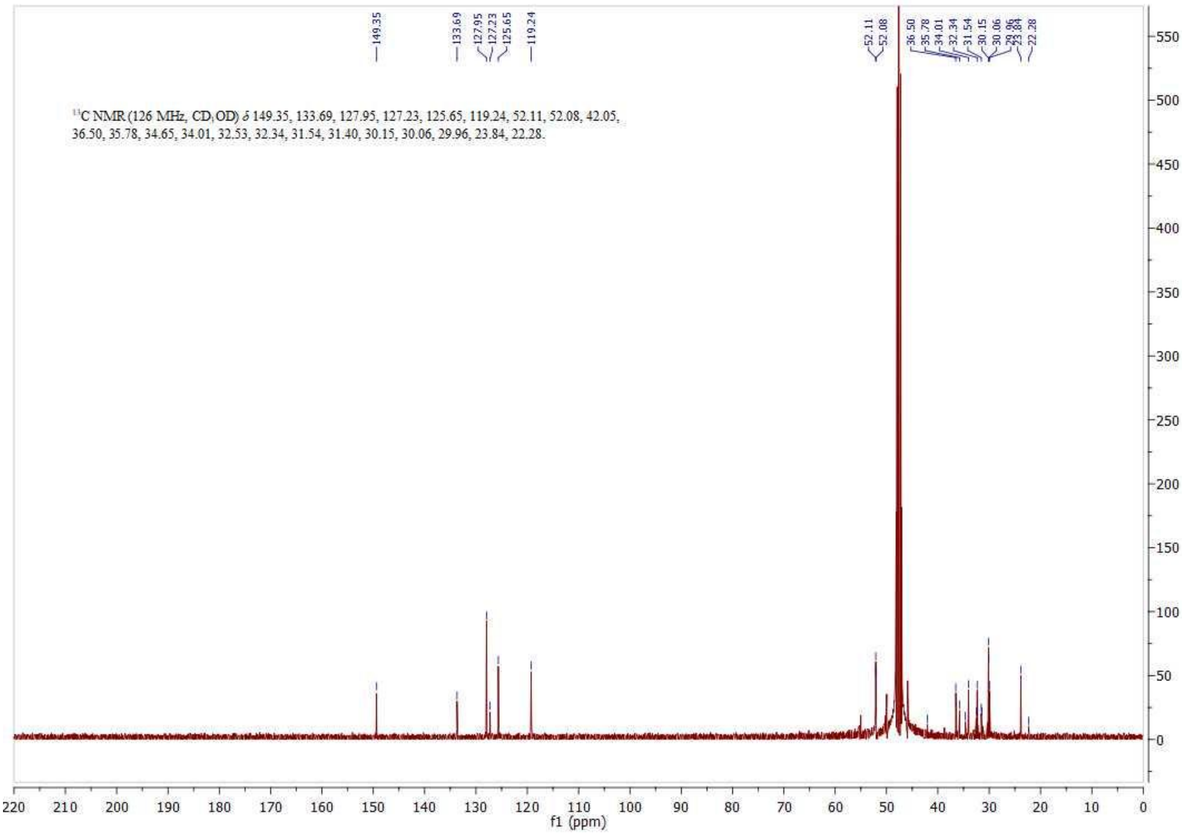

*1-(6-((3-(3-((1R,4R)-4-(2-(4-(2,3-dichlorophenyl)piperazin-1-yl)ethyl)cyclohexyl)-1-methylureido)propyl)amino)-6-oxohexyl)-3,3-dimethyl-2-((1E,3E)-5-((Z)-1,3,3-trimethyl-5-sulfoindolin-2-ylidene)penta-1,3-dien-1-yl)-3H-indol-1-ium-5-sulfonate (**18**)*

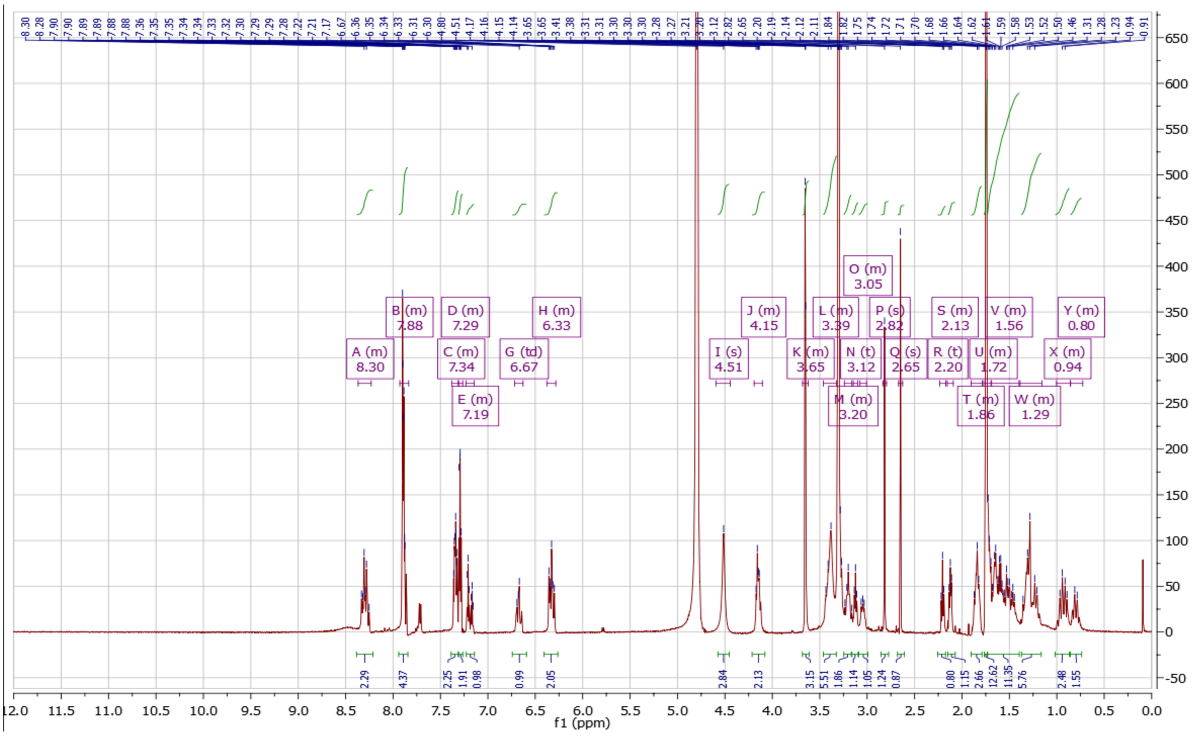

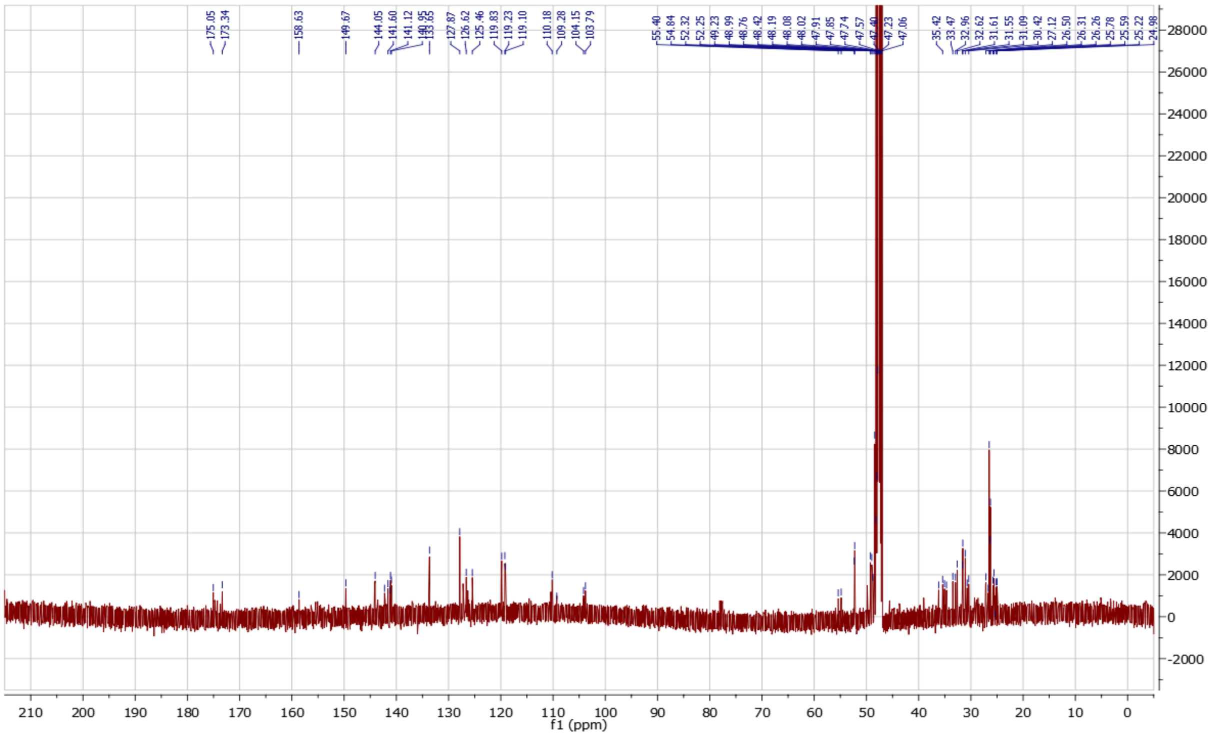

*(4-(2-Azidoethoxy)phenyl)(4-chlorophenyl)methanol (**28**)*

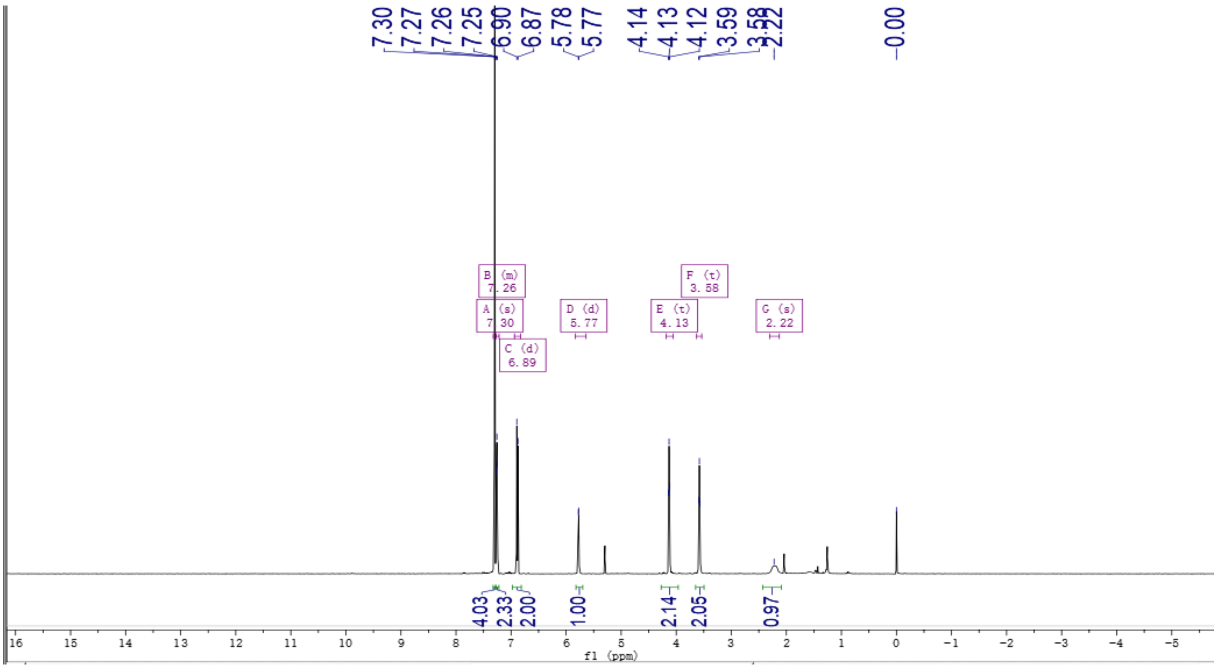

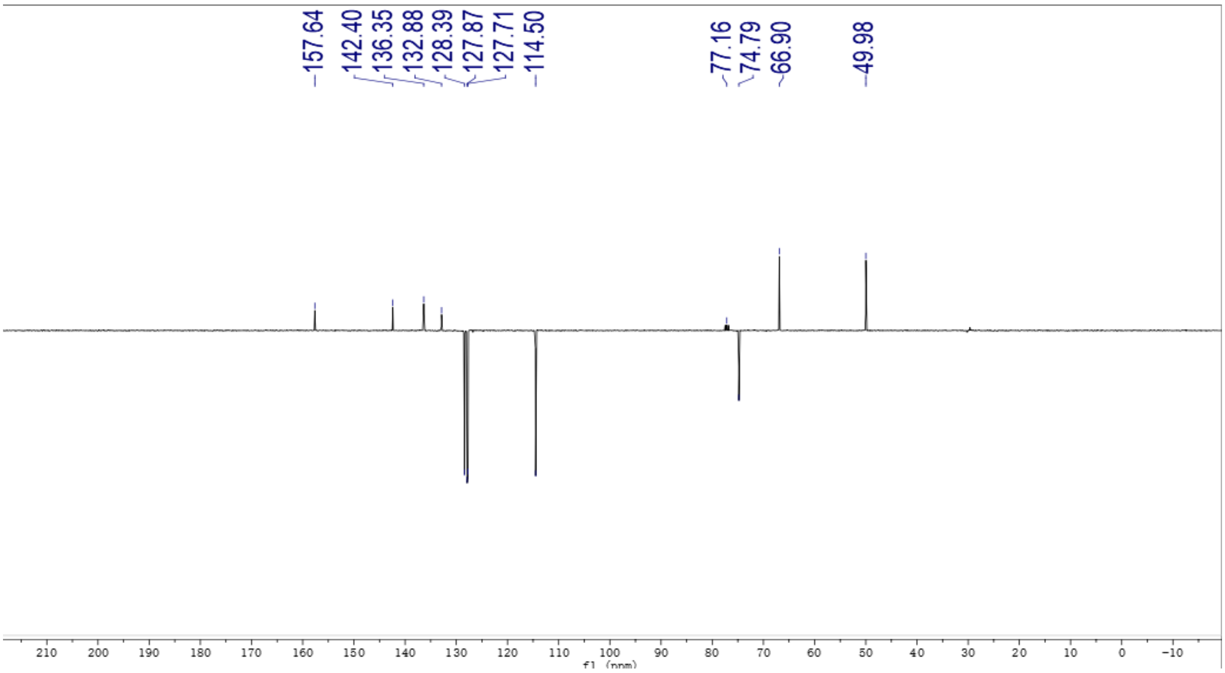

*tert-Butyl 4-((4-(2-azidoethoxy)phenyl)(4-chlorophenyl)methyl)piperazine-1-carboxylate (**30**)*

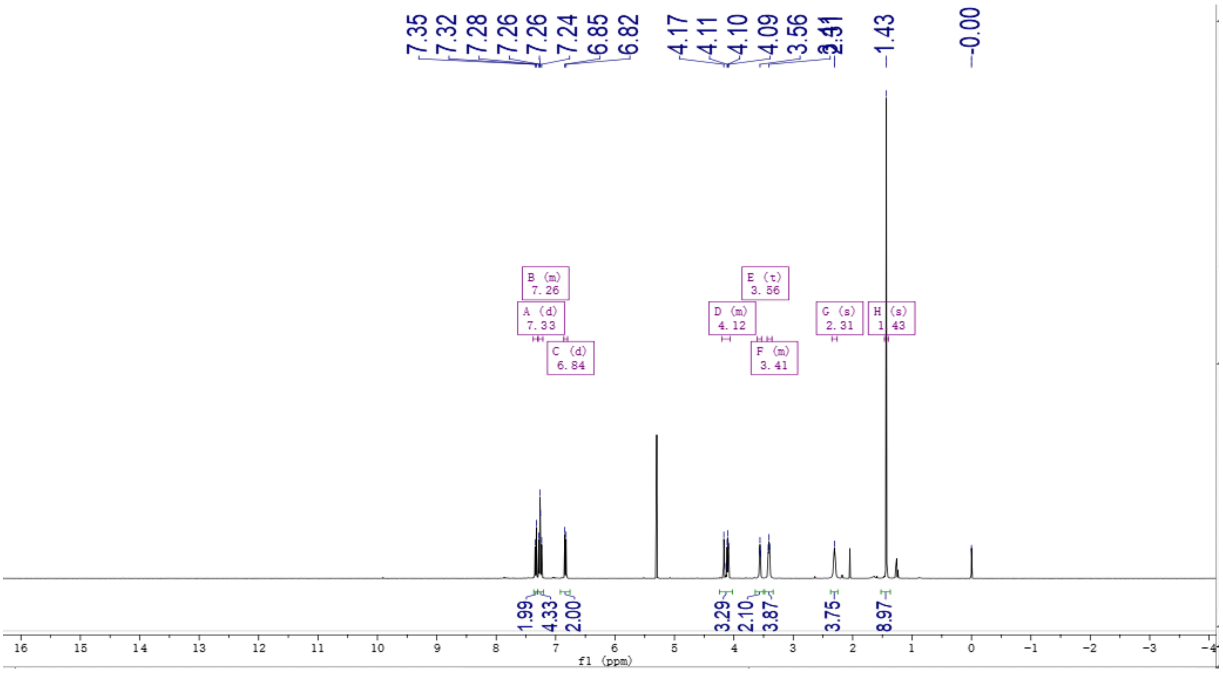

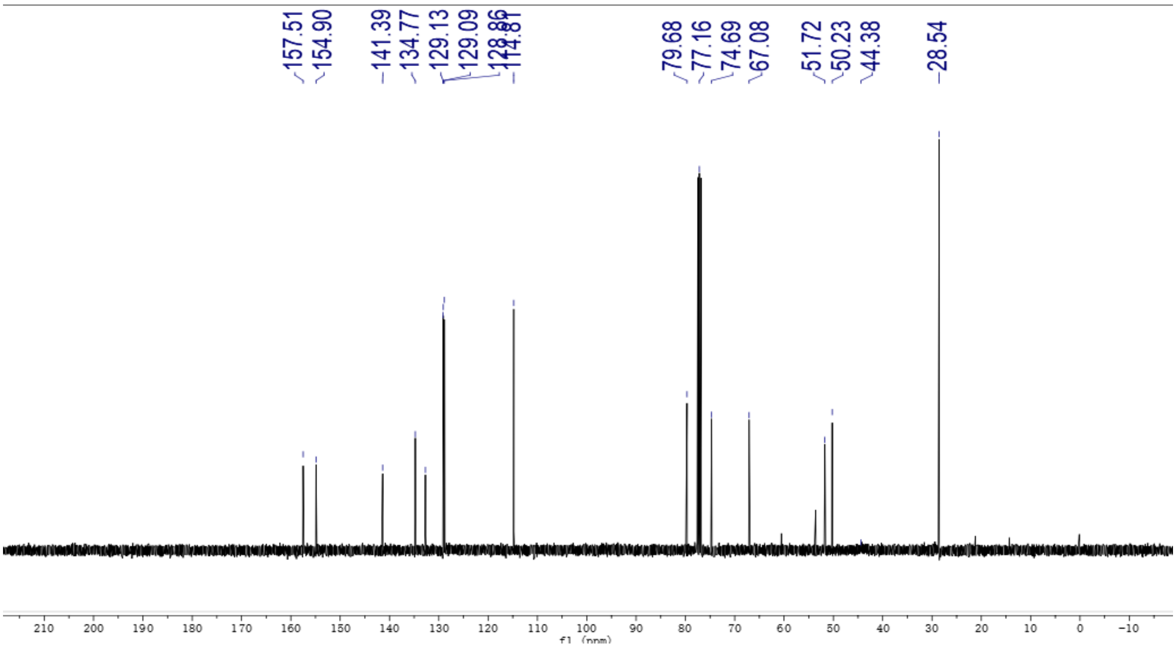

*1-((4-(2-Azidoethoxy)phenyl)(4-chlorophenyl)methyl)piperazine (**31**)*

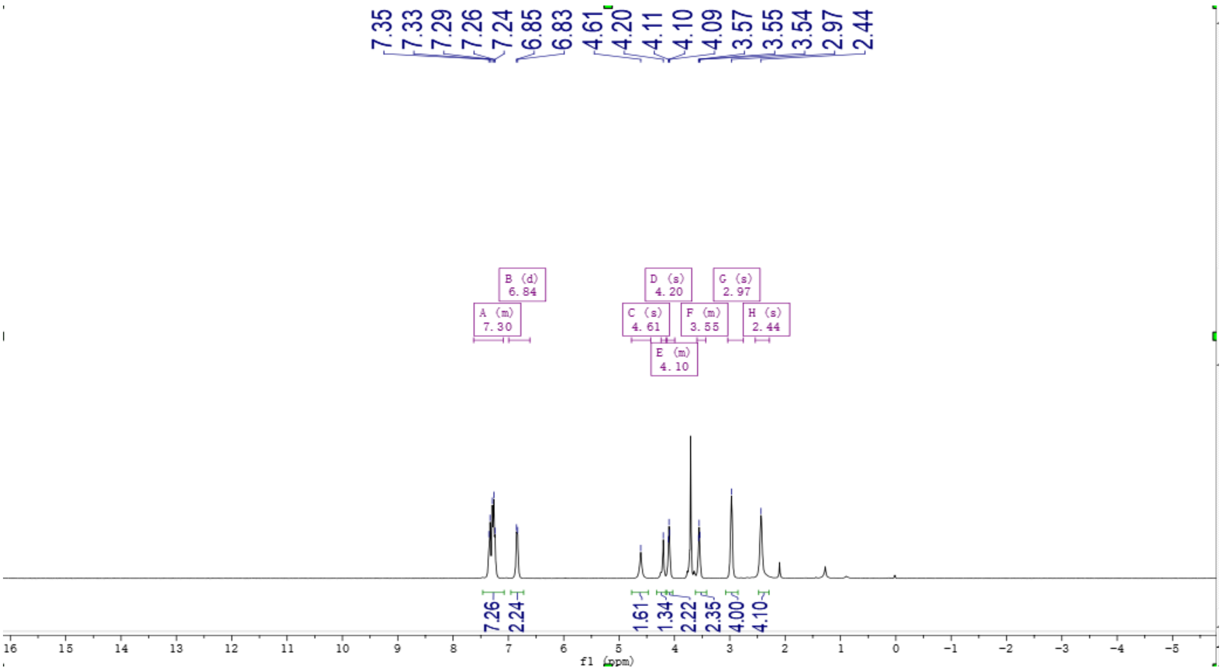

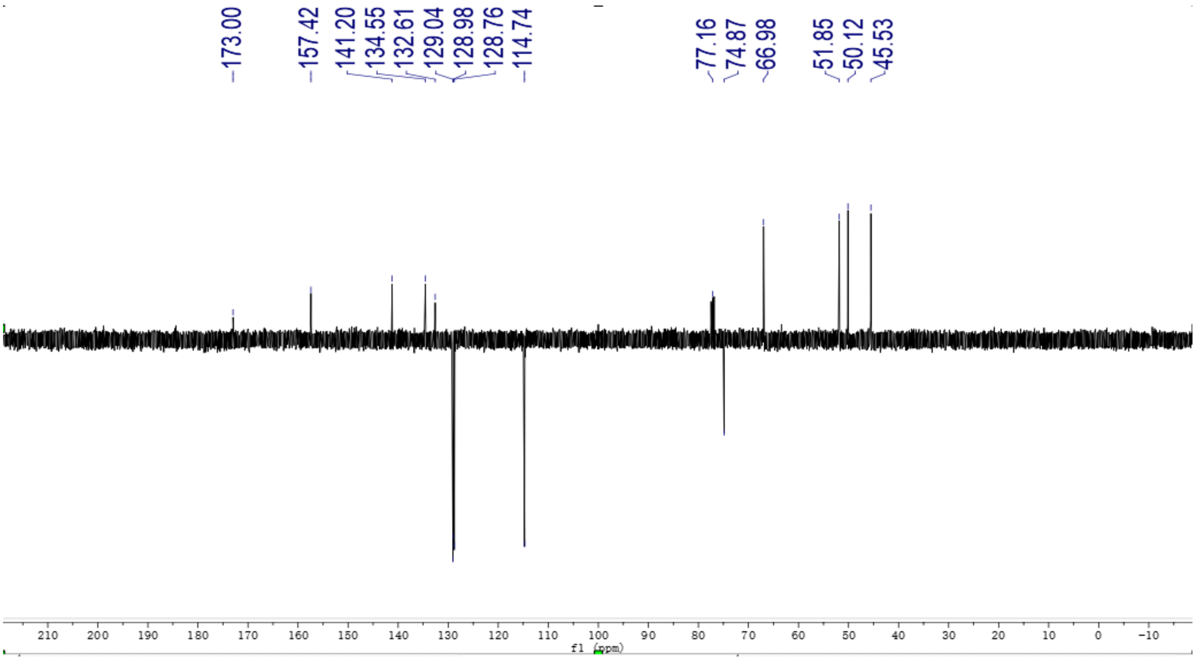

*1,1,1,3,3,3-Hexafluoropropan-2-yl 4-((4-(2-azidoethoxy)phenyl)(4-chlorophenyl)methyl) piperazine-1-carboxylate (**32**)*

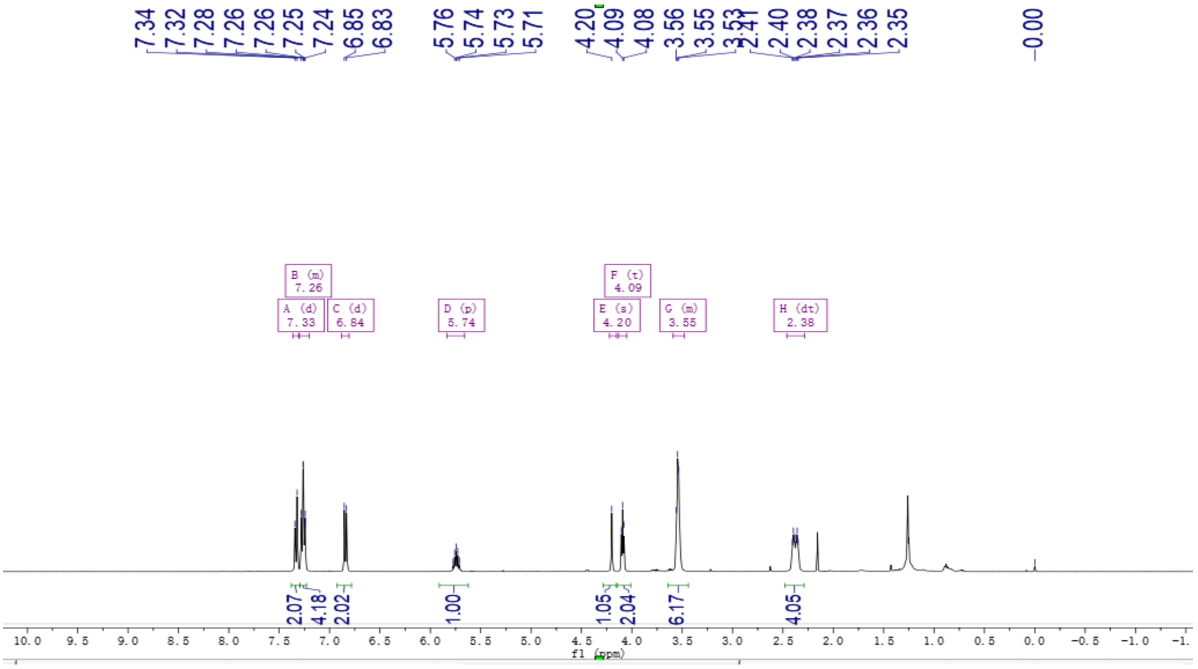

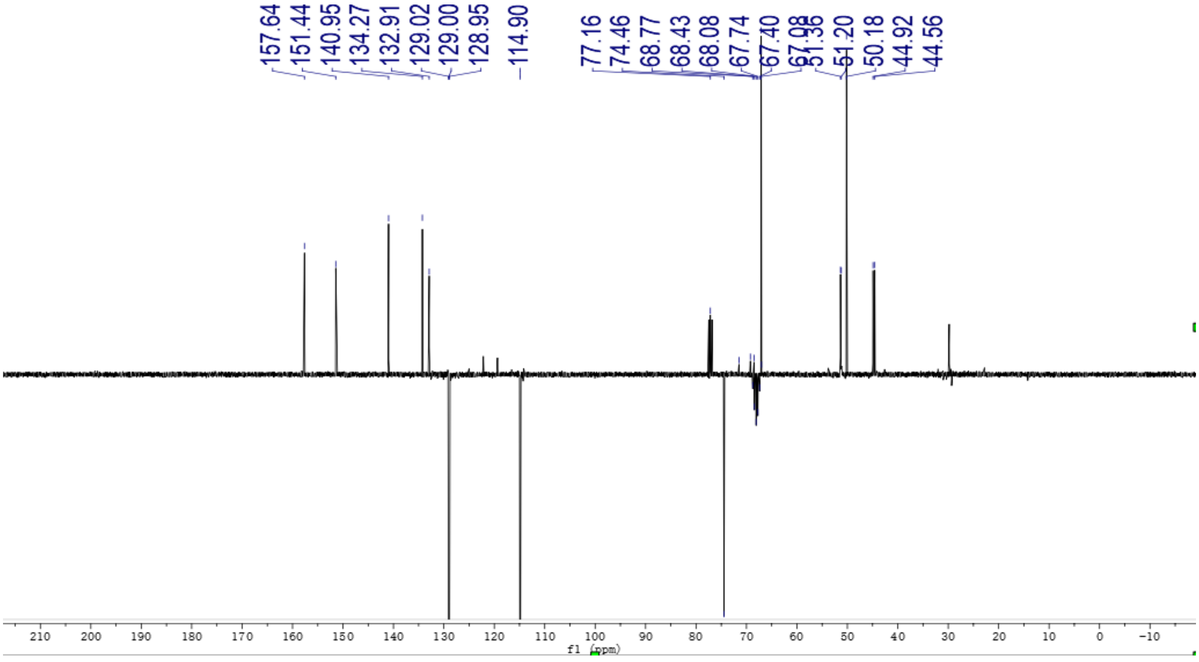

*1-(6-(((1-(2-(4-((4-Chlorophenyl)(4-(((1,1,1,3,3,3-hexafluoropropan-2-yl)oxy)carbonyl)piperazin-1-yl)methyl)phenoxy)ethyl)-1H-1,2,3-triazol-4-yl)methyl)amino)-6-oxohexyl)-3,3-dimethyl-2-((1E,3E)-5-((E)-1,3,3-trimethylindolin-2-ylidene)penta-1,3-dien-1-yl)-3H-indol-1-ium chloride (**DH-463**) (**34**)*

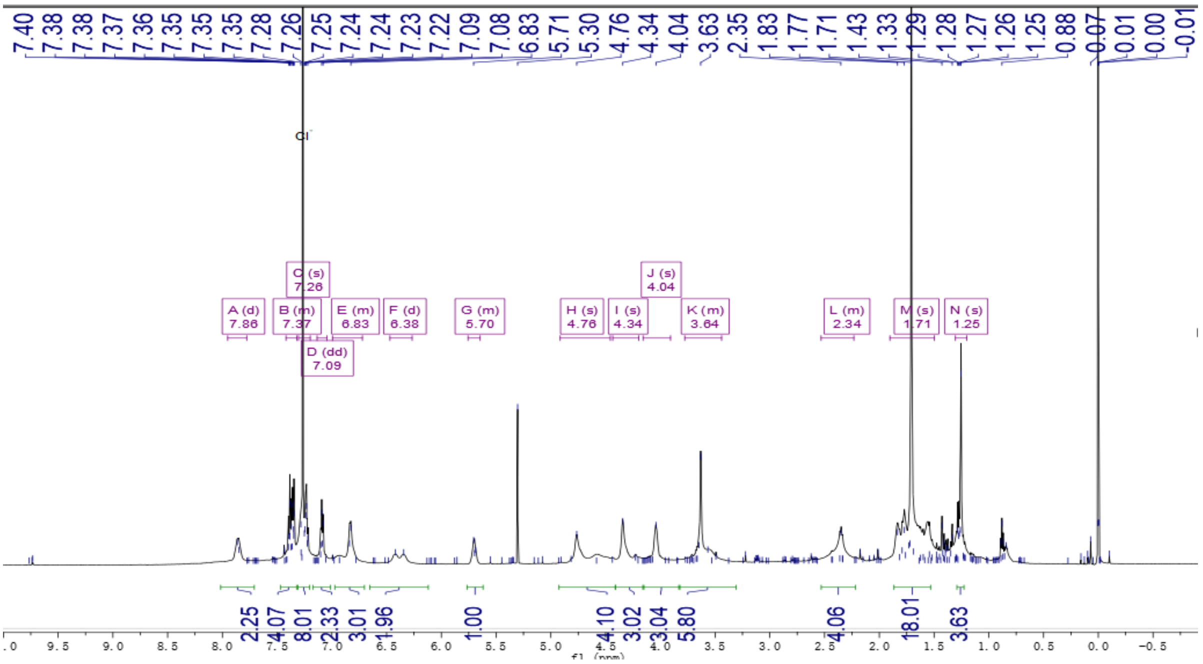

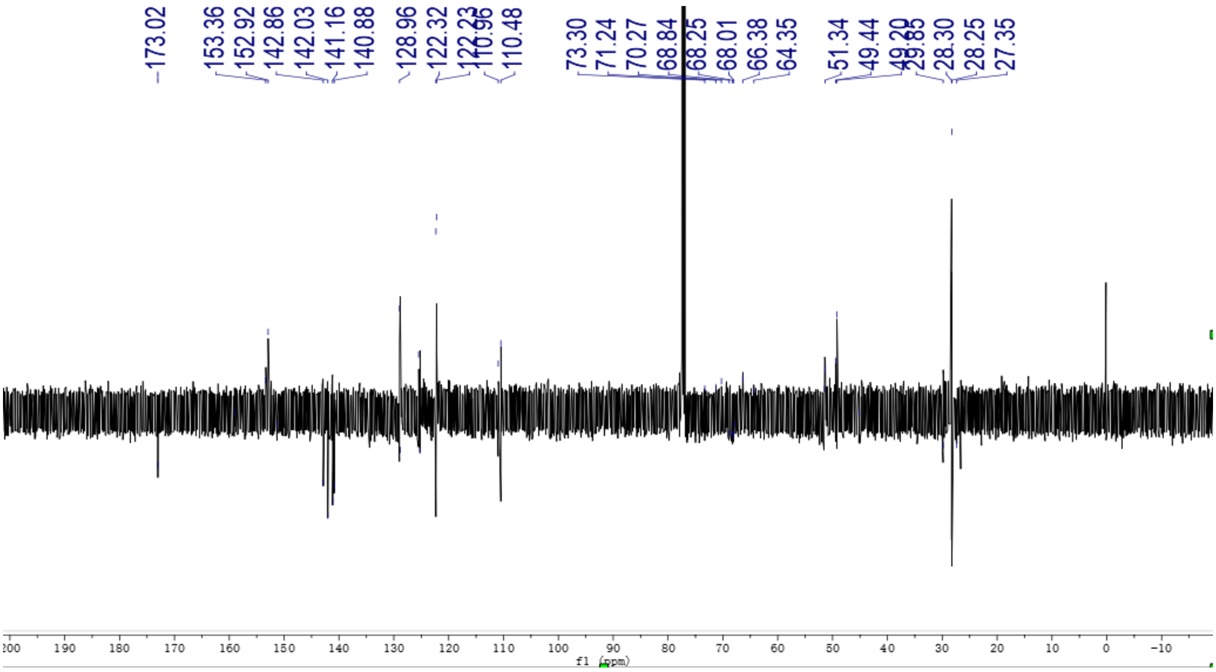

(H) Supplementary References

S1. Jun, J. V, Chenoweth, D. M. & Petersson, E. J. Rational design of small molecule fluorescent probes for biological applications. *Org. Biomol. Chem* **18**, 5747 (2020).

S2. Kobayashi, H., Ogawa, M., Alford, R., Choyke, P. L. & Urano, Y. New Strategies for Fluorescent Probe Design in Medical Diagnostic Imaging. *Chem. Rev.* **110**, 2620-40 (2010)

S3. Blagg, J. & Workman, P. Choose and Use Your Chemical Probe Wisely to Explore Cancer Biology. (2017). *Cancer Cell.* **32**, 9-25 (2017)

S4. Prevet, H. & Collins, I. Labelled chemical probes for demonstrating direct target engagement in living systems. *Future Med. Chem.* **11**, 1195–1224 (2019)

S5. Castaldi, M. P., Hendricks, J. A. & Zhang, A. X. 'Design, synthesis, and strategic use of small chemical probes toward identification of novel targets for drug development'. *Current Opinion in Chemical Biology* **56**, 91–97 (2020).

S6. Arrowsmith, C. H. *et al.* The promise and peril of chemical probes. *Nat. Chem. Biol.* **11**, 536–541 (2015).

S7. Conner, J. W., Poole, D. P., Jörg, M. & Veldhuis, N. A. New small molecule fluorescent probes for G protein-coupled receptors: valuable tools for drug discovery. *Future Med. Chem.* **13**, 63-90 (2020).

S8. Xu, J., Ma, H. & Liu, Y. Stochastic Optical Reconstruction Microscopy (STORM). *Curr. Protoc. Cytom.* **81**, 12.46.1-12.46.27 (2017).

S9. Dempsey, G. T., Vaughan, J. C., Chen, K. H., Bates, M. & Zhuang, X. Evaluation of fluorophores for optimal performance in localization-based super-resolution imaging. *Nat. Methods* **8**, 1027–1036 (2011).

S10. Fu, Y. & Finney, N. S. Small-molecule fluorescent probes and their design. *Roy Soc Chem Adv* **8**, 29051-29061 (2018).

S11. Zhang, M., Li, X., Gao, G. & Deng, C. Simultaneous Determination of Multiple Biomarkers for Breast Cancer Based on a Dual-Tagged Fluorescent Probe. *Anal. Lett.* **53**, 371–384 (2020).

S12. Zettlitz, K. A. *et al.* A dual-modality linker enables site-specific conjugation of antibody fragments for 18 F-immunoPET and fluorescence imaging. *J. Nucl. Med.* **60**, 1467-1473 (2019).

S13. Schreiber, S. L. *et al.* Advancing biological understanding and therapeutics discovery with small-molecule probes. *Cell* **161**, 1252–1265 (2015).

S14. Gleeson, M. P., Hersey, A., Montanari, D. & Overington, J. Probing the links between in vitro potency, ADMET and physicochemical parameters. *Nat. Rev. Drug Discov.* **10**, 197–208 (2011).

S15. Zhang, T., Hatcher, J. M., Teng, M., Gray, N. S. & Kostic, M. Recent Advances in Selective and Irreversible Covalent Ligand Development and Validation. *Cell Chem. Biol.* **26**, 1486-1500 (2019).

S16. Bunnage, M. E., Chekler, E. L. P. & Jones, L. H. Target validation using chemical probes. *Nature Chemical Biology* **9**, 195–199 (2013).

S17. Garbaccio, R. M. & Parmee, E. R. The Impact of Chemical Probes in Drug Discovery: A Pharmaceutical Industry Perspective. *Cell Chemical Biology* **23**, 10–17 (2016).

S18. Iliopoulos-Tsoutsouvas, C., Kulkarni, R. N., Makriyannis, A. & Nikas, S. P. Fluorescent probes for G-protein-coupled receptor drug discovery. *Expert Opinion on Drug Discovery* **13**, 933–947 (2018).

S19. Sarott, R. *et al.* Development of High-Specificity Fluorescent Probes to Enable Cannabinoid Type 2 Receptor Studies in Living Cells. *J. Am. Chem. Soc.* **142**, 16953-1616964 (2020).

S20. Azuaje, J. *et al.* Development of Fluorescent Probes that Target Serotonin 5-HT2B Receptors. *Sci. Rep.* **7**, 10765 (2017).

S21. Madhavi Sastry, G., Adzhigirey, M., Day, T., Annabhimoju, R. & Sherman, W. Protein and ligand preparation: Parameters, protocols, and influence on virtual screening enrichments. *J. Comput. Aided. Mol. Des.* **27**, 221–234 (2013).

S22. Verdonk, M. L., Cole, J. C., Hartshorn, M. J., Murray, C. W. & Taylor, R. D. Improved protein-ligand docking using GOLD. *Proteins Struct. Funct. Genet.* **52**, 609–623 (2003).

S23. Friesner, R. A. *et al.* Glide: A New Approach for Rapid, Accurate Docking and Scoring. 1. Method and Assessment of Docking Accuracy. *J. Med. Chem.* **47**, 1739–1749 (2004).

S24. Friesner, R. A. *et al.* Extra precision glide: Docking and scoring incorporating a model of hydrophobic enclosure for protein-ligand complexes. *J. Med. Chem.* **49**, 6177–6196 (2006).
S25. Harvey, M. J., Giupponi, G. & De Fabritiis, G. ACEMD: Accelerating biomolecular dynamics in the microsecond time scale. *J. Chem. Theory Comput.* **5**, 1632–1639 (2009).
S26. Lomize, M. A., Lomize, A. L., Pogozheva, I. D. & Mosberg, H. I. OPM: Orientations of proteins in membranes database. *Bioinformatics* **22**, 623–625 (2006).
S27. Jo, S., Kim, T., Iyer, V. G. & Im, W. CHARMM-GUI: A web-based graphical user interface for CHARMM. *J. Comput. Chem.* **29**, 1859–1865 (2008).
S28. Hua, T. *et al.* Crystal structures of agonist-bound human cannabinoid receptor CB 1. *Nature* **547**, 468–471 (2017).
S29. Mahadevan, A. *et al.* Novel cannabinol probes for CB1 and CB2 cannabinoid receptors. *J. Med. Chem.* **43**, 3778–3785 (2000).
S30. Schrödinger Release 2017-4: LigPrep, Schrödinger,LLC, New York, NY, **2017**
S31. Schrödinger Release 2017-4: Glide, Schrödinger, LLC, New York, NY, **2017**.
S32. Iovine, V. *et al.* Total Synthesis of (±)-Kuwanol E. *J. Nat. Prod.* **79**, 2495–2503 (2016).
S33. Nicolaou, K. C., Koide, K. & Bunnage, M. E. Total Synthesis of Balanol and Designed Analogues. *Chem. - A Eur. J.* **1**, 454–466 (1995).
S34. B. Kiss, *et al.* Cariprazine (RGH-188), a Dopamine D_3 Receptor-Preferring, D_3/D_2 Dopamine Receptor Antagonist–Partial Agonist Antipsychotic Candidate: In Vitro and Neurochemical Profile. *J. Pharmacol. Exp. Ther.* **333**, 328–340 (2010).
S35. Chien, E. Y. T. *et al.* Structure of the human dopamine D3 receptor in complex with a D2/D3 selective antagonist. *Science* **330**, 1091–5 (2010).
S36. Wang, S. et al. Structure of the D2 dopamine receptor bound to the atypical antipsychotic drug risperidone. *Nature* **555**, 269–273 (2018).
S37. Wang, S. et al. D4 dopamine receptor high-resolution structures enable the discovery of selective agonists. *Science* **358**, 381–386 (2017).
S38. Pándy-Szekeres, G. *et al.* GPCRdb in 2018: adding GPCR structure models and ligands. *Nucleic Acids Res.* **46**, D440–D446 (2018).
S39. Frank, A., Kiss, D. J., Keserű, G. M. & Stark, H. Binding kinetics of cariprazine and aripiprazole at the dopamine D3 receptor. *Sci. Rep.* **8**, 12509 (2018).
S40. García-Nafría, J., Nehmé, R., Edwards, P. C. & Tate, C. G. Cryo-EM structure of the serotonin 5-HT1B receptor coupled to heterotrimeric Go. *Nature* **558**, 620–623 (2018).
S41. Šali, A. & Blundell, T. L. Comparative Protein Modelling by Satisfaction of Spatial Restraints. *J. Mol. Biol.* **234**, 779–815 (1993).
S42. Jacobson, M. P. *et al.* A hierarchical approach to all-atom protein loop prediction. *Proteins Struct. Funct. Bioinforma.* **55**, 351–367 (2004).
S43. Czibula, L. *et al.* Piperazine salt and a process for the preparation thereof. WO2010/70369, A1 (2010).
S44. Liu, K., Kokubo, H. Exploring the Stability of Ligand Binding Modes to Proteins by Molecular Dynamics Simulations: A Cross-docking Study. *J. Chem. Inf. Model.* **57**, 2514–2522 (2017).
S45. Solecka, J. *et al.* New Derivatives of 3,4-Dihydroisoquinoline-3-carboxylic Acid with Free-Radical Scavenging, D-Amino Acid Oxidase, Acetylcholinesterase and Butyrylcholinesterase Inhibitory Activity. *Molecules* **19**, 15866–15890 (2014).
S46. Lai, X.-Z., Makriyannis, A. & Lu, D. Keto Cannabinoids with therapeutic indications. WO2004/17922, A2 (2004).
