# Supplementary material for: PharmacoSTORM nanoscale pharmacology reveals cariprazine binding on Islands of Calleja granule cells
Source: Nat Commun. 2021 Nov 11;12:6505. doi: 10.1038/s41467-021-26757-z (PMC8586358; doi:10.1038/s41467-021-26757-z)
Supplement: Supplementary file 3 — Reporting Summary [file 41467_2021_26757_MOESM3_ESM.pdf]

nature research

Corresponding author(s): Istvan Katona
Last updated by author(s): Sept 17, 2021

Reporting Summary

Nature Research wishes to improve the reproducibility of the work that we publish. This form provides structure for consistency and transparency in reporting. For further information on Nature Research policies, see our Editorial Policies and the Editorial Policy Checklist.

Statistics

For all statistical analyses, confirm that the following items are present in the figure legend, table legend, main text, or Methods section.

n/a	Confirmed	
☐	☒	The exact sample size (n) for each experimental group/condition, given as a discrete number and unit of measurement
☐	☒	A statement on whether measurements were taken from distinct samples or whether the same sample was measured repeatedly
☐	☒	The statistical test(s) used AND whether they are one- or two-sided<br>*Only common tests should be described solely by name; describe more complex techniques in the Methods section.*
☒	☐	A description of all covariates tested
☐	☒	A description of any assumptions or corrections, such as tests of normality and adjustment for multiple comparisons
☐	☒	A full description of the statistical parameters including central tendency (e.g. means) or other basic estimates (e.g. regression coefficient) AND variation (e.g. standard deviation) or associated estimates of uncertainty (e.g. confidence intervals)
☐	☒	For null hypothesis testing, the test statistic (e.g. F, t, r) with confidence intervals, effect sizes, degrees of freedom and P value noted<br>*Give P values as exact values whenever suitable.*
☒	☐	For Bayesian analysis, information on the choice of priors and Markov chain Monte Carlo settings
☒	☐	For hierarchical and complex designs, identification of the appropriate level for tests and full reporting of outcomes
☒	☐	Estimates of effect sizes (e.g. Cohen's d, Pearson's r), indicating how they were calculated

Our web collection on statistics for biologists contains articles on many of the points above.

Software and code

Policy information about availability of computer code

Data collection	Microscopic imaging: Nikon A1R confocal laser-scanning system built on a Ti-E inverted microscope operated by NIS-Elements AR software 4.50.00 (Nikon); 3DHISTECH Panoramic MIDI II slide scanner; Ti-E inverted microscope equipped with an N-STORM system, a Nikon C2 confocal scan head, and an Andor iXon Ultra 897 EMCCD camera operated by NIS-Elements AR software 4.51 (Nikon).<br>Other: ChemiDoc MP system; Thermo Scientific Varioskan Flash multimode plate reader; Thermo Scientific Luminoskan Ascent microplate luminometer; MultiClamp 700B amplifier; Digidata 1440A.
Data analysis	The following softwares and codes were used in data analysis and data presentation:<br>Image Lab 4.1; 3D Slicer 4.10.2; Blender 2.80; NIS Elements AR 5.21.01 (Nikon); Visual Molecular Dynamics (VMD) 1.9.3; Neurolucida version 2020.2.2; GraphPad Prism 5 and 9; VividSTORM 1.3; Clampfit 10; Python 2.7- 3.8; Schrödinger Release 2017-4: LigPrep, Schrödinger,LLC, New York, NY, 2017; Schrödinger Release 2017-4: Glide, Schrödinger, LLC, New York, NY, 2017; Custom Python-based scripts were written for easier and quicker handling of files and to analyse STORM images indicated in the Methods section.

For manuscripts utilizing custom algorithms or software that are central to the research but not yet described in published literature, software must be made available to editors and reviewers. We strongly encourage code deposition in a community repository (e.g. GitHub). See the Nature Research guidelines for submitting code & software for further information.

Data

Policy information about availability of data

All manuscripts must include a data availability statement. This statement should provide the following information, where applicable:

- Accession codes, unique identifiers, or web links for publicly available datasets
- A list of figures that have associated raw data
- A description of any restrictions on data availability

Source data are provided for the paper, which include all statistical results for the following figure panels: Fig.1a,d,g,j; Fig.2b,c,f,g,i; Fig.3i,j;k Fig.4d,f; Fig.5d,e; Fig.7g,j; Suppl. Fig.S2b,d,f,h,j,l; Suppl. Fig.S3b,c,d,e; Suppl. Fig.S5b,d,f,g,i,k; Suppl. Fig.S6b,d; Suppl. Fig.S7c,e; Suppl. Fig.S8a,b,e,f; Suppl. Fig.S9c; Suppl. Fig.S11c; Suppl. Fig.S10c,e,f,g; Suppl. Fig.S11c; Suppl. Fig.S12c. NMR spectra for all synthesized compounds are provided in the Supplementary Information. Further data that support the findings of this study are available from the corresponding author upon reasonable request.

Field-specific reporting

Please select the one below that is the best fit for your research. If you are not sure, read the appropriate sections before making your selection.

☒ Life sciences ☐ Behavioural & social sciences ☐ Ecological, evolutionary & environmental sciences

For a reference copy of the document with all sections, see nature.com/documents/nr-reporting-summary-flat.pdf

Life sciences study design

All studies must disclose on these points even when the disclosure is negative.

Sample size	We used sample sizes that are widely accepted in the field and in identical cellular and animal models in our laboratory (Dudok et al., Nature Neuroscience 18, 75-86 (2015); Prokop et al., Cell Signal 36, 98–107 (2017)).
Data exclusions	All data obtained from cell culture samples or acute mouse brain slices were included in the analysis unless technical issues with the samples (cell viability problems, weak transfection efficiency, lack of healthy neurons) would have prohibited rationale scientific conclusions. It was decided objectively by expert researchers of the field and not based on pre-established criteria.
Replication	All experiments in this study were replicated succesfully at least three times (three independent cell-cultures or animals.) All attempts at replication were succesful. Source data or figure legends contain the exact number of samples and animals used.
Randomization	All animals, or cells in cultures for imaging were selected randomly.
Blinding	The investigators were not blinded during data collection. Blinding was not relevant as the same treatment and analysis parameters were used across the compared samples. In slice preparation, the striking biological differences between the genotypes (WT and KO) also rendered blind analysis impossible.

Reporting for specific materials, systems and methods

We require information from authors about some types of materials, experimental systems and methods used in many studies. Here, indicate whether each material, system or method listed is relevant to your study. If you are not sure if a list item applies to your research, read the appropriate section before selecting a response.

Materials & experimental systems

n/a	Involved in the study	
☐	☒	Antibodies
☐	☒	Eukaryotic cell lines
☒	☐	Palaeontology and archaeology
☐	☒	Animals and other organisms
☒	☐	Human research participants
☒	☐	Clinical data
☒	☐	Dual use research of concern

Methods

n/a	Involved in the study	
☒	☐	ChIP-seq
☒	☐	Flow cytometry
☒	☐	MRI-based neuroimaging

Antibodies

Antibodies used	Primary antibodies:<br>anti-DARPP-32 (Abcam, #ab220808, Lot: GR3175773-1)<br>anti-DAT (Frontier Institute, #DAT-GP-Af660)<br>anti-GFP (Abcam, #ab5450 Lot: GR3251233-1, GR3215617-1)<br>anti-HA (SIGMA #H3663, clone HA-7, Lot:038M4810V)

anti-MAP2 (Chemicon/ Millipore, #AB5622, Lot:2795016)
anti-nNOS (Abcam, #ab1376, Lot: GR3195323-9, GR3195323-11)
anti-TH (Immunostar, #22941, Lot:1552001, 1814001)

Secondary antibodies:
Alexa Fluor® 488 AffiniPure Donkey Anti-Rabbit IgG (Jackson ImmunoResearch, 711-545-152)
CF®568-Donkey Anti-Mouse IgG (Biotium, 20105)
CF®568-Donkey Anti-Goat IgG (Biotium , 20106)
Alexa Fluor® 488 AffiniPure Donkey Anti-Goat IgG (Jackson ImmunoResearch, 705-545-147)
Cy™5 AffiniPure Donkey Anti-Mouse IgG (Jackson ImmunoResearch, 715-175-151)
Alexa Fluor® 647 AffiniPure Donkey Anti-Guinea Pig IgG (H+L) (Jackson ImmunoResearch, 706-605-148)
Alexa Fluor® 647 AffiniPure Donkey Anti-Mouse IgG (H+L) (Jackson ImmunoResearch, 715-605-150)

Validation

Validation information and previously published applications are described on the manufacturer`s websites. The distribution of immunolabeled cells and subcellular profiles observed in the present study corroborated previously published results in case of all antibodies.

Eukaryotic cell lines

Policy information about cell lines

Cell line source(s)

HEK 293 cells were a kind gift from Balázs Gereben (Institute of Experimental Medicine, Budapest and were originally obtained fom American Type Culture Collection (ATCC CRL-1573). HEK 293T cells were obtained from American Type Culture Collection (ATCC CRL-3216).

Authentication

The cell lines were not authenticated in the authors laboratory.

Mycoplasma contamination

PCR-based tests were performed on a monthly basis to check for Mycoplasma contamination, and gave negative results.

Commonly misidentified lines
(See ICLAC register)

No misidentified lines were used in the study.

Animals and other organisms

Policy information about studies involving animals; ARRIVE guidelines recommended for reporting animal research

Laboratory animals

C57BL/6 mice were obtained from Charles River Laboratories. The mouse line bearing a targeted mutation in the Drd3 gene has been validated in earlier studies (Accili, D. et al. Proc. Natl. Acad. Sci. U. S. A. 93, 1945–1949 (1996)). Mice were kept under specific-pathogen-free conditions at the Medical Genetics Unit of IEM. 25-57 days old male and female mice were used.

Wild animals

The study did not involve wild animals.

Field-collected samples

The study did not involve samples collected from the field.

Ethics oversight

All experiments were approved by the Hungarian Committee of the Scientific Ethics of Animal Research (license number: 2018/1 internal license number and PE_EA_49-5_2020), and were carried out according to the Hungarian Act of Animal Care and Experimentation (1998, XXVIII, Section 243/1998), in accordance with the European Communities Council Directive of 24 November 1986 (86-609-EEC; Section 243/1998).

Note that full information on the approval of the study protocol must also be provided in the manuscript.
